# Charge Resonance Interaction in Aromatic Trimer Radical Cations Revealed by IR Spectroscopy: The Case of Pyrrole Homo‐ and Heterotrimers

**DOI:** 10.1002/chem.71324

**Published:** 2026-07-01

**Authors:** Dashjargal Arildii, Otto Dopfer

**Affiliations:** ^1^ Institut für Physik und Astronomie Technische Universität Berlin Berlin Germany

**Keywords:** charge density, chemistry, delocalized electron, dimer, hydrogen bond, infrared spectroscopy, intermolecular force, ionic bonding, radical ion, trimer

## Abstract

Intermolecular interactions of aromatic molecules are crucial for many biological and functional materials. One of the strongest intermolecular forces is the charge resonance in an aromatic dimer radical cation (CR, ∼100 kJ mol^−1^), in which the positive charge is delocalized over both molecules. It is of interest how a third unit influences the CR and binding motif of a dimer cation, including the degree of charge delocalization. Herein, we investigate the binding motifs, energies, charge distribution, and origin of the intermolecular forces of the pyrrole trimer cation (Py_3_^+^). The NH stretch frequencies (*ν*_NH_) recorded by infrared spectroscopy provide insight into the preferred binding motifs and interaction strengths by comparison to density functional theory calculations. We extend the study to Py_2_^+^X by substituting one Py in Py_3_^+^ with toluene or benzene. In both homo‐ and heterotrimers, the third unit solvates the CR‐stabilized Py_2_^+^ by an NH…π ionic hydrogen bond (H‐bond) rather than by CR or π‐stacking. This NH…π H‐bond has only a minor effect on the CR of the Py_2_^+^ core, because the positive charge is retained in the dimer core. Minor charge transfer to the third unit causes an uneven charge distribution in the Py_2_^+^ core, thereby reducing its symmetry.

## Graphical abstract

We demonstrate the power of high‐resolution infrared spectroscopy and complementary quantum chemical calculations to unravel the evolution of the aromatic charge resonance interaction (CR) in cold trimer radical cations for the prototypical pyrrole clusters. The third ligand acts essentially as a hydrogen‐bonded solvent of the CR‐bonded dimer cation, on which the positive charge remains localized.

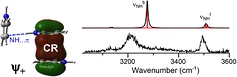

## 1 Introduction

Noncovalent intermolecular interactions involving aromatic π electrons, such as π‐stacking and π‐hydrogen bonds (π H‐bonds), play a crucial role in determining the structures and properties of a vast number of biological and functional materials. Such aromatic interactions are vital for many chemical and biological processes, including protein folding, molecular recognition, molecular assembly, and crystal growth in both liquid and solid phases [1, 2, 3, 4, 5, 6, 7, 8, 9, 10, 11, 12, 13, 14, 15, 16, 17]. In addition to π‐stacking and π H‐bonds, the charge‐resonance (CR) interaction is one of the strongest forces in charged aromatic dimers, with binding energies of the order of ∼100 kJ mol^−1^ [18, 19, 20, 21]. Thus, aromatic CR interactions significantly contribute to the structure and energetics of intermolecular and intramolecular stacking interactions as well as charge transport in conjugated biological and organic semiconductor materials [22, 23, 24, 25, 26, 27, 28, 29, 30].

Upon removal of a single electron in aromatic homo‐ or heterodimer cations (A_2_^+^, AB^+^), the CR is formed in a stacked configuration by sharing the positive charge between the two monomer units, resulting in a splitting between two electronic states into a stabilized ground state (ψ_+_) and a destabilized and repulsive excited state (ψ_−_). For A_2_^+^, their wavefunctions are described as [18, 20, 31, 32, 33]

$$\;\psi_{\pm }=\psi \left(A^{+}\right)\psi \left(A\right)\;\pm \;\psi \left(A\right)\psi \left(A^{+}\right).$$

The intense electronic CR transition (ψ_−_ ← ψ_+_) has been measured for various aromatic A_2_^+^ and AB^+^ dimer cations using optical spectroscopy. It typically occurs around 1 µm and is found to be broad even in the gas phase at low temperature because it involves excitation into the repulsive ψ_−_ state [32, 33, 34, 35, 36, 37, 38]. Hence, the optical CR bands usually provide only limited information about both the structures and the charge distributions of the dimer cations. The same is true for mass spectrometric experiments, which only yield binding energies of the CR interaction [21, 39, 40, 41].

To overcome the inherent limitations of electronic spectroscopy, we recently introduced high‐resolution infrared photodissociation (IRPD) spectroscopy as a versatile tool for analyzing the charge distributions and geometric changes in isolated aromatic dimer cations by exciting vibrational transitions in the stable ground state (ψ_+_) of the CR dimer rather than exciting into the repulsive ψ_−_ state [20, 42, 43]. In these studies, the pyrrole dimer cation (Py_2_^+^) serves as a suitable prototype to characterize the aromatic CR using IR spectroscopy because Py has a single isolated, uncoupled, and strongly IR‐active NH stretch oscillator, whose frequency depends sensitively on its charge state (e.g., *ν*_NH_ = 3531, 3480, 3447 cm^−1^ for *q*_Py_/*e* = 0, 0.5, 1) [20, 44, 45]. In contrast, the CH stretch modes (*ν*_CH_) of benzene (Bz) and other bare polycyclic aromatic hydrocarbons are less sensitive to their charge state [32, 36, 46, 47, 48, 49]. Indeed, *ν*_NH_ of Py and *q*_Py_ exhibit a nearly linear correlation expressed as *ν*_NH_(*q*_Py_)/cm^−1^ = −83.8 *q*_Py_/*e* + 3527.8 [20], which allowed us to further characterize the evolution of the CR of Py_2_^+^ by either solvation (with Ar, N_2_, H_2_O) or substitution of functional groups (NH → NCH_3_) [20, 42, 43, 50]. In general, the more positive the partial charge on the Py unit (*q*_Py_), the lower its free NH stretch frequency (*ν*_NH_).

Py_2_^+^ forms the CR‐stabilized π–π sandwich structure with antiparallel and parallel configurations, Py_2_^+^(a/p), of similar binding energies (*D*_0_ = ∼100 kJ mol^−1^) rather than a T‐shaped NH…π H‐bonded structure observed for its Py_2_ neutral counterpart (*D*_0_ = ∼20 kJ mol^−1^) [42, 51, 52], revealing the drastic influence of the CR on both the dimer binding motif and the interaction strength. Furthermore, under our experimental conditions, Py_2_^+^(a) predominates the Py_2_^+^ population over Py_2_^+^(p) because it is more stable by Δ*E*_0_ = 7.5 kJ mol^−1^. In Py_2_^+^(a), the CR is stronger as each Py unit carries +0.5 *e* owing to its higher symmetry compared to Py_2_^+^(p) (*C*_2h_ vs. *C*_1_, Figure S1) [42]. In solvated Py_2_^+^L*_n_* clusters with L_*n*_ = Ar_1–5_, N_2_, or (H_2_O)_1–3_, the aromatic binding motif is also governed by the CR interaction, with the ligands L*_n_* solvating the CR‐stabilized stacked Py_2_^+^ core. The excess charge in Py_2_^+^L*_n_* remains mainly localized on the Py_2_^+^ core even for relatively strong perturbations caused by these noncovalent interactions with the ligands (e.g., *D*_0_ (Py_2_^+^H_2_O) = 46 kJ mol^−1^), highlighting the robustness of the CR interaction in the dimer cations [42, 43]. Therefore, it is particularly interesting to explore the evolution of the CR in the pyrrole trimer radical cation (Py_3_^+^), including the effect of the third Py unit on the CR interaction, binding motif, charge distribution, and degree of charge delocalization.

In previous work, the CR interactions in the benzene and naphthalene trimer cations (Bz_3_^+^ and Np_3_^+^) were studied in the gas phase using mass spectrometry and electronic photodissociation spectroscopy in the ultraviolet to near‐IR range [32, 53, 54, 55, 56, 57, 58, 59, 60]. The broad absorption bands reveal that the positive charge remains largely localized in the dimer cation, while the third unit simply solvates the CR‐stabilized dimer cation. However, acidic Py clusters differ notably from both Bz and Np clusters because the NH group of Py enables an additional NH…π H‐bonding motif. For example, neutral Py_3_ forms a symmetric NH…π H‐bonded triangular structure (*C*_3h_) rather than a π‐stacked structure [51, 52, 61]. Upon ionization, Py_3_^+^ is predicted to adopt a binding motif, in which the Py_2_^+^(a) core is solvated by the third Py unit via an NH…π H‐bond [62]. However, a recent study that measured an IRPD spectrum of Py_3_^+^ clusters generated by vacuum ultraviolet (VUV) ionization using 118 nm reported that the third Py unit mainly solvates isomeric structures of Py_2_^+^ denoted cc1 and cn1 with newly formed covalent C─C and C─N bonds between two Py units [63]. These were argued to arise from complex intracluster ion‐molecule reactions triggered by VUV ionization of the neutral precursor. Only a minor contribution was actually assigned to unreacted Py_3_^+^ clusters, in which the third Py solvates the Py_2_^+^(p) core via an NH…π H‐bond [63].

To shed further light on the possible binding motifs of Py_3_^+^ and study the influence of the CR interaction on the trimer configurations, we investigate herein Py_3_^+^ and its colder Ar‐tagged cluster generated in an electron ionization (EI) source using IRPD spectroscopy of mass‐selected clusters in the structure‐sensitive NH stretch range (*ν*_NH_). Furthermore, we extend our study to heterotrimers, Py_2_^+^X with X = Bz and toluene (Tol), for comparison. Complementary dispersion‐corrected density functional theory (DFT) calculations are employed to assign the measured IRPD spectra and to analyze the observed intermolecular interactions and charge distributions. We aim to elucidate the relationship between the CR interaction and the binding motifs of Py_3_^+^ and its Py_2_^+^X derivatives. Furthermore, we explore the influence of the third aromatic unit on the formation of the Py_2_^+^(a/p) configurations and their associated charge distributions. Significantly, high‐resolution IRPD is utilized herein for the first time to characterize the CR interaction in trimer cations in great detail, which goes significantly beyond the previous low‐resolution studies of Bz_*n*_^+^ clusters [54, 55, 56, 57, 58, 59].

## 2 Experimental and Computational Details

IRPD spectra of mass‐selected Py_3_^+^, Py_3_^+^Ar, and Py_2_^+^X (X = Bz and Tol) cluster cations are obtained in a tandem quadrupole mass spectrometer coupled to an EI source and an octupole ion guide [9, 64]. The cluster ions are produced in a pulsed supersonic plasma expansion of Py (Sigma–Aldrich, >98%) and Bz (Fluka, ≥99.5%) or Tol (Sigma–Aldrich, ≥99.5%), seeded in Ar carrier gas (7 bar, nozzle temperature 50°C). Ionic clusters are formed by electron and/or chemical ionization and subsequent three‐body aggregation reactions. The cluster cations of interest are selected by the first quadrupole mass filter and irradiated in the adjacent octupole with a tunable IR laser pulse (*ν*_IR_) emitted from an optical parametric oscillator and amplifier pumped by a Q‐switched nanosecond Nd:YAG laser. The IR radiation is characterized by a pulse energy of 2–5 mJ in the considered 2700–3600 cm^−1^ range, a bandwidth of <2 cm^−1^, and a repetition rate of 10 Hz. The IR laser frequency is calibrated to better than 1 cm^−1^ using a wavemeter. Resonant vibrational excitation induces predominantly the loss of the most weakly bonded ligand (i.e., Ar, Py, Bz, or Tol). The generated photofragment ions are then selected by the second quadrupole mass filter and monitored by a Daly detector as a function of *ν*_IR_ to obtain the IRPD action spectrum of the parent cluster. The ion source is triggered at twice the laser frequency to subtract the metastable decay background from the total fragment ion signal for extracting the laser‐induced dissociation signal. All IRPD spectra are linearly normalized for frequency‐dependent variations in the IR photon flux measured by a pyroelectric detector. The peak widths of the vibrational transitions observed in the IRPD spectra are mainly caused by unresolved rotational structure, sequence hot bands involving inter‐ and intramolecular modes, and unresolved contributions of possible isomers. In addition to laser‐induced dissociation (LID and IRPD), low‐energy collision‐induced dissociation (CID) experiments are performed in the octupole ion guide to confirm the composition of the mass‐selected parent clusters. For recording CID mass spectra, the octupole is filled with 10^−5^ mbar air, resulting in ion‐neutral collisions with 10 eV energy in the laboratory frame. Representative LID spectra of Py_3_^+^, Py_3_^+^Ar, and Py_2_^+^Bz are shown in Figure S2.

Quantum chemical calculations are carried out at the dispersion‐corrected B3LYP‐D3/aug‐cc‐pVTZ level to determine the structural, energetic, vibrational, and electronic properties of the investigated cluster cations using the GAUSSIAN16 package [65, 66, 67, 68, 69]. Energies of the optimized structures obtained using the CAM‐B3LYP‐D3 [69], CBS‐QB3 [70, 71], and single‐point DLPNO‐CCSD(T) [72, 73] approaches are employed to evaluate the reliability of the computational results at the B3LYP‐D3 level. Harmonic vibrational frequencies computed at the B3LYP‐D3/aug‐cc‐pVTZ level are scaled by a factor of 0.9619 for NH and CH stretch vibrations to optimize the agreement with the measured frequency of Py (*ν*_NH_ = 3531 cm^−1^) [44]. Intermolecular interaction energies (*D*_0_) and relative energies (*E*_0_) are corrected for harmonic zero‐point vibrational energy. Relative free energies (*G*_0_) are calculated for room temperature (298 K). The charge distributions on each molecular component are obtained employing the natural bond orbital (NBO) analysis at the B3LYP‐D3 and CAM‐B3LYP‐D3 levels [74]. Potential energy surfaces for intracluster isomerization are computed at the B3LYP‐D3/aug‐cc‐pVTZ level using the nudged elastic band method implemented in the ORCA package [75, 76]. The intermolecular interactions are analyzed using the noncovalent interaction (NCI) approach [77, 78, 79], and details are given in the Supporting Information. The dispersion contributions to the total dissociation energies are estimated by performing the geometry optimizations with and without the empirical dispersion correction (D3) at the corresponding DFT levels [80]. Cartesian coordinates of all considered isomers and their corresponding energies are provided in the Supporting Information.

## 3 Results and Discussion

The IRPD spectra of Py_3_^+^, Py_3_^+^Ar, and Py_2_^+^X (X = Tol or Bz) shown in Figure 1 are recorded in the 2700–3600 cm^−1^ range to cover the informative free and bound (i.e., H‐bonded) NH stretch modes, *ν*_NH_^f^ (E) and *ν*_NH_^b^ (F), as well as the less structure‐sensitive aromatic CH stretch modes, *ν*_CH_ (G). For comparison, the positions of *ν*_NH_^f^ of Py^+^ (3447 cm^−1^ [44]), the CR‐stabilized Py_2_^+^ dimer (3480 cm^−1^ [20]), and Py (3531 cm^−1^ [51]) are indicated by gray dashed lines. The positions and widths of the transitions observed are compiled in Tables 1 and S1, along with their suggested vibrational and isomer assignments.

**FIGURE 1:**
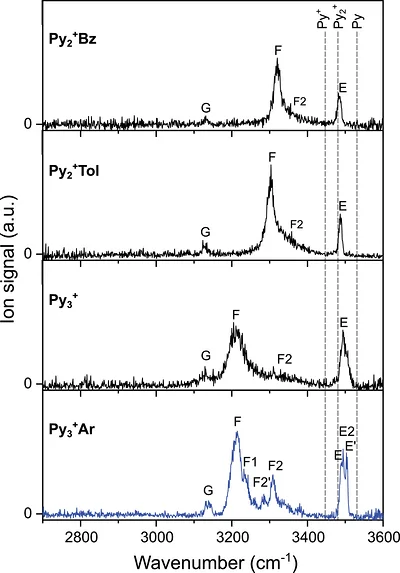
IRPD spectra of Py_3_^+^(Ar), Py_2_^+^Tol, and Py_2_^+^Bz recorded in the CH and NH stretch range. The positions, widths, and assignments of the transitions observed are compiled in Tables 1 and S1. For comparison, the *ν*_NH_^f^ frequency of Py^+^ (3447 cm^−1^), Py_2_^+^(a) (3480 cm^−1^), and Py (3531 cm^−1^) are indicated by a gray dashed line.

**TABLE 1:** Positions (in cm^−1^) and suggested vibrational assignments of the NH stretch transitions observed in the IRPD Spectra of Py_3_^+^(Ar), Py_2_^+^Tol, and Py_2_^+^Bz compared to frequencies of the most stable isomers calculated at the B3LYP‐D3/aug‐cc‐pVTZ level.^a^

	Exp^b^	Isomer	Calc^c^	Mode
Py_3_^+^	F 3214 (54)	Py_3_^+^(a)(I) Py_3_^+^(a)(II)	3276 (1026) 3262 (1002)	ν_NH_ ^b^
	F2 3328 (nr)	Py_3_^+^(p)(I)	3315 (270), 3349 (1050)	ν_NH_ ^b^
	E 3496 (19)	Py_3_^+^(a)(I) Py_3_^+^(a)(II) Py_3_^+^(p)(I)	3506 (142), 3512 (100) 3506 (144), 3512 (70) 3507 (98)	ν_NH_^f^
Py_3_^+^Ar	F 3214 (27)	Py_3_^+^(a)(II)Ar(I)	3264 (1023)	ν_NH_ ^b^
	F1 3234 (15)	Py_3_^+^(a)(I)Ar(I)	3282 (1048)	ν_NH_ ^b^
	F2′ 3286 (8)	Py_3_^+^(p)(I)Ar(I)	3315 (330)	ν_NH_ ^b^
	F2 3309 (19)	Py_3_^+^(p)(I)Ar(I)	3351 (947)	ν_NH_ ^b^
	E 3489 (9)	Py_3_^+^(a)(I)Ar(I) Py_3_^+^(a)(I)Ar(II)	3505 (176) 3504 (171)	ν_NH_^f^
	E2 3494 (3)	Py_3_^+^(p)(I)Ar(I)	3508 (100)	ν_NH_^f^
	E′ 3504 (7)	Py_3_^+^(a)(I)Ar(I) Py_3_^+^(a)(I)Ar(II)	3512 (100) 3512 (69)	ν_NH_^f^
Py_2_^+^Tol	F 3304 (29)	Py_2_^+^(a)Tol(I)	3379 (711)	ν_NH_ ^b^
	E 3487 (9)	Py_2_^+^(a)Tol(I)	3505 (147)	ν_NH_^f^
Py_2_^+^Bz	F 3321 (23)	Py_2_^+^(a)Bz(I)	3386 (651)	ν_NH_ ^b^
	E 3484 (10)	Py_2_^+^(a)Bz(I)	3505 (146)	ν_NH_^f^

^a^ See Table S1 for the complete list.

^b^ Peak positions and widths (in parentheses) are obtained from deconvolution. nr = not resolved, broad.

^c^ IR intensities in parentheses are given in km mol^−1^.

### 3.1 Py_3_^+^

The IRPD spectrum of Py_3_^+^ recorded in the Py_2_^+^ fragment channel exhibits three major peaks at 3496 (E), 3214 (F), and 3130 (G) cm^−1^ with widths of 19–54 cm^−1^, and one broad feature at 3328 cm^−1^ (F2). Overall, the positions of these four bands (and their relative intensities) are consistent with those observed in the IRPD spectrum reported previously for Py_3_^+^ ions generated by VUV ionization of neutral clusters (3489, 3224, 3132, 3316 cm^−1^) [63], and a comparison of both spectra is presented in Figure S3. However, there are also significant differences between the two spectra with respect to both appearance and interpretation. First, our EI‐IRPD spectrum is much better resolved with sharper transitions, indicating that the Py_3_^+^ ions generated in our EI plasma expansion are significantly colder than those prepared by VUV ionization. VUV ionization of neutral clusters at 118 nm (around 10.5 eV) in the collision‐free region of the expansion introduces quite some excess energy into the cluster (the ionization energy of Py_3_ is computed to be around 7.59 eV [62]), resulting in vibrationally hot clusters and clusters undergoing intracluster chemical reactions [63]. When considering the ionization energy of Py_3_ up to 2.9 eV excess energy are available upon VUV ionization for vibrational heating, dissociation, and overcoming barriers for chemical reactions. Additionally, the observed Py_3_^+^ clusters may also be produced from the fragmentation of larger clusters [63]. In contrast, Py_3_^+^ ions are generated in our EI source by EI of Py followed by sequential addition of two further Py units accompanied by collisional cooling. Thus, the EI source produces much colder and (almost) exclusively unreacted Py_3_^+^ ions. Second, peaks observed at 2806 and 2959 cm^−1^ in the VUV‐IRPD spectrum [63], which have been assigned to isomers with chemically bonded structures, are completely absent in our EI‐IRPD spectrum of Py_3_^+^, confirming that such reacted isomers do indeed not form in our EI source. Third, the interpretation of peak F at 3214 cm^−1^ in the EI‐IRPD spectrum of Py_3_^+^ differs from the interpretation for the corresponding 3224 cm^−1^ band in the VUV‐IRPD spectrum (assigned to a band of chemically reacted cc1/cn1‐Py isomers) [63]. Peak F in the EI‐IRPD spectrum exhibits a strongly blueshaded band contour and a significant red shift of −266 cm^−1^ from *ν*_NH_^f^ of Py_2_^+^, suggesting an assignment to *ν*_NH_^b^ of an unreacted Py_3_^+^ isomer with a NH…π H‐bond. Similarly, the band of the less strongly redshifted peak F2 (−152 cm^−1^) may arise from another H‐bonded Py_3_^+^ isomer with a somewhat weaker NH…π H‐bond. The central maximum of the unresolved free *ν*_NH_^f^ band E is substantially blueshifted (+49 cm^−1^) from that observed for the Py^+^ monomer cation (3496 vs. 3447 cm^−1^), indicating that the positive charge within Py_3_^+^ is largely delocalized within the trimer and not concentrated on a single Py unit. On the other hand, the modest *ν*_NH_^f^ blueshift of +16 cm^−1^ of Py_3_^+^ from that of the CR‐stabilized Py_2_^+^ dimer (Figure 1) suggests that attachment of the third Py molecule has only a minor effect on the CR in Py_2_^+^, similar to the previous observation for Py_2_^+^H_2_O [43]. In addition, the asymmetric and unresolved broad profile of band E implies the presence of multiple free NH stretch bands. To confirm the structural and vibrational assignments, the IR spectra computed for the lowest‐energy structures of Py_3_^+^ (Figures 2 and S4) are compared with the measured IRPD spectrum in Figures 3 and S5 (Tables 1 and S1, S2).

**FIGURE 2:**
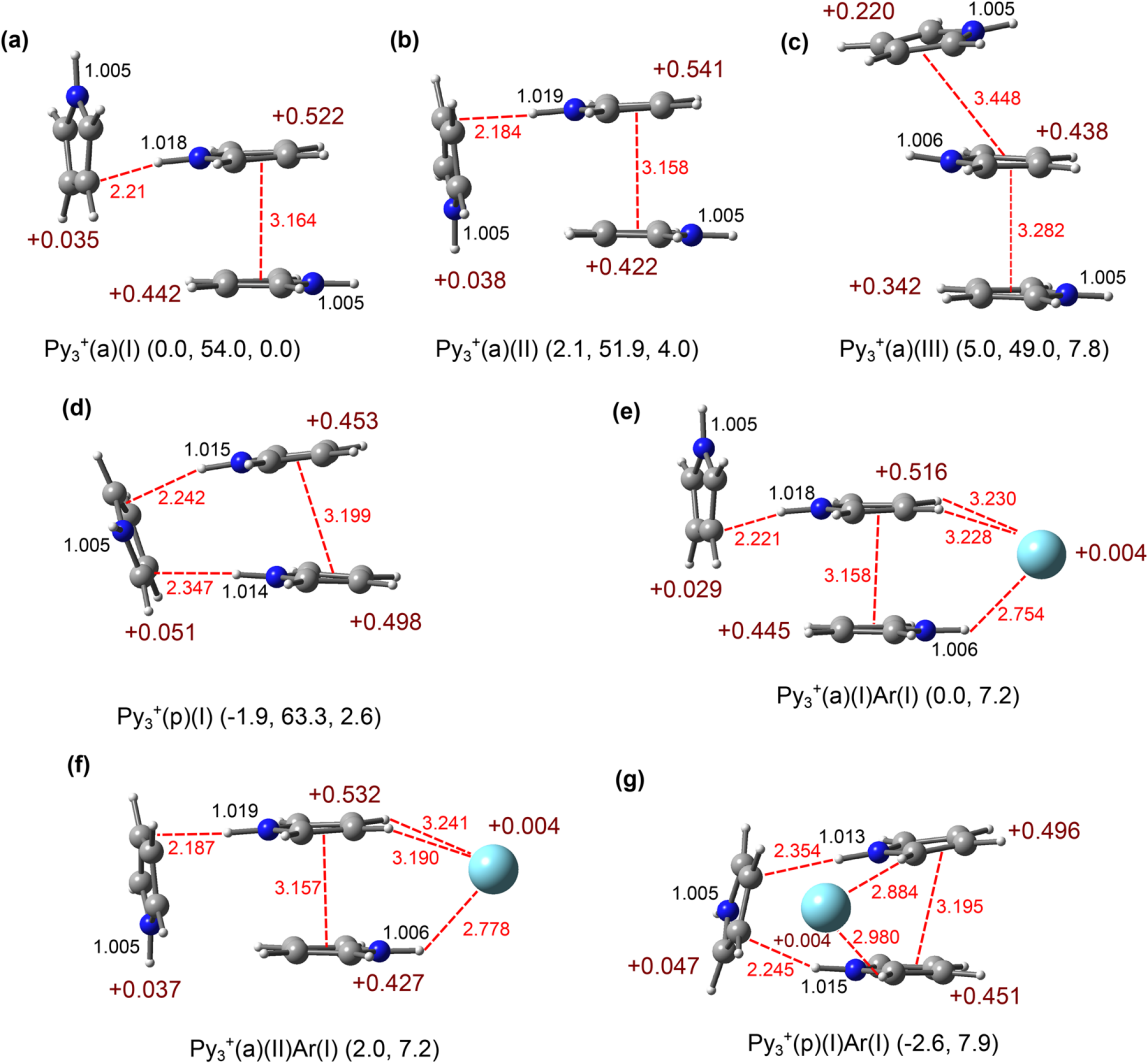
Structures of (a–c) Py_3_^+^(a)(I‐III), (d) Py_3_^+^(p)(I), (e‐f) Py_3_^+^(a)(I‐II)Ar(I), and (g) Py_3_^+^(p)(I)Ar(I) obtained at the B3LYP‐D3/aug‐cc‐pVTZ level. Selected intramolecular and intermolecular bond lengths (in Å) are given in black and red, respectively. Values in dark red indicate NBO charges (in units of *e*). Energies in parentheses are relative energy referenced to Py_3_^+^(a)(I) for (a–d) and Py_3_^+^(a)(I)Ar(I) for (e–g), dissociation energy of the most weakly bonded ligand, and relative Gibbs free energy at 298 K (*E*_0_, *D*_0_, *G*_0_ in kJ mol^−1^). *D*_0_ accounts for the loss of Py for (a–d) and Ar for (e–g), respectively.

**FIGURE 3:**
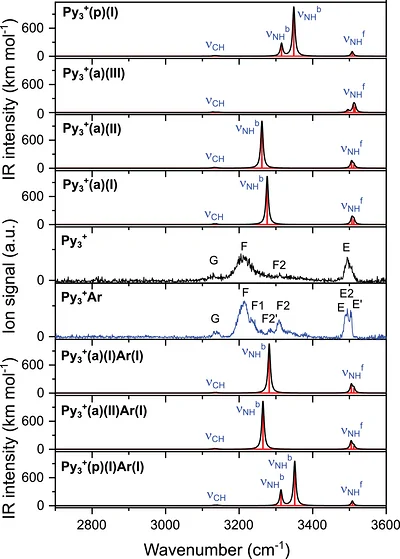
IRPD spectra of Py_3_^+^(Ar) recorded in the CH and NH stretch range compared to linear IR absorption spectra calculated for various isomers (Figure 2) at the B3LYP‐D3/aug‐cc‐pVTZ level (Tables 1 and S1).

The conformational search for Py_3_^+^ isomers is conducted based on the CR‐stabilized Py_2_^+^(a/p) minima (Figure S1), and the obtained structures are denoted as Py_3_^+^(a) and Py_3_^+^(p), respectively (Figures 2 and S4). In Py_3_^+^(a)(I), the third Py unit interacts with the NH group of one of the Py units of Py_2_^+^(a) via an NH…π H‐bond with *D*_0_ = 54.0 kJ mol^−1^. The N─H donor bond of Py_2_^+^(a) elongates by 12 mÅ (1.018 vs. 1.006 Å), which leads to a computed redshift of −224 cm^−1^ for *ν*_NH_^b^ (3276 cm^−1^), in line with the experimental shift of −266 cm^−1^. This slight discrepancy between predicted and observed shift is primarily due to the deficiency of the harmonic scaling approach to fully account for the highly anharmonic intermolecular interaction potential of this mode. The free N─H bond of Py_2_^+^(a) contracts slightly due to the reduced partial charge (0.442 vs. 0.5 *e*), resulting in a small blueshift of +6 cm^−1^ (3506 cm^−1^). The newly formed NH…π H‐bond causes an uneven charge distribution (0.522 vs. 0.442 *e*) in the Py_2_^+^(a) moiety. A similar trend in the charge distribution of Py_2_^+^(a) is observed for the related NH…O H‐bonded Py_2_^+^(a)H_2_O trimer [43]. A small charge transfer (0.035 *e*) to the neutral Py unit via the NH…π H‐bond elongates its N─H bond by 3 mÅ, leading to a modest computed redshift for *ν*_NH_^f^ of Py (3512 vs. 3531 cm^−1^) [43, 51]. In the only slightly less stable Py_3_^+^(a)(II) isomer (*D*_0_ = 51.9 kJ mol^−1^, *E*_0_ = 2.1 kJ mol^−1^), the third Py solvates Py_2_^+^(a) also through an NH…π H‐bond but differs in the orientation (essentially obtained by 180° internal rotation compared to isomer I). As a result, the charge transfer to neutral Py increases to 0.038 *e*, leading to a slightly greater perturbation of the CR in Py_2_^+^(a). Therefore, the H‐bond donor N─H bond elongates more than in Py_3_^+^(a)(I) by 1 mÅ, resulting in a slightly larger redshift of* ν*_NH_^b^ (−238 cm^−1^). The *ν*_NH_^f^ modes of Py_3_^+^(a)(II) are predicted at the same frequency as for Py_3_^+^(a)(I) at 3506 and 3512 cm^−1^. In Py_3_^+^(a)(III), the third Py binds through π–π interaction to Py_2_^+^(a) with an antiparallel orientation because of the favorable alignment of the electric dipole moments of the Py units. However, the electron‐rich π‐ring of the third Py tilts toward the H‐bond donor NH group of Py_2_^+^(a), causing poor π–π overlap between the Py_2_^+^(a) core and the third Py. As a result, Py_3_^+^(a)(III) is slightly less stable than Py_3_^+^(a)(I) by 5.0 kJ mol^−1^ (*D*_0_ = 49.0 kJ mol^−1^). Because the CR is not efficiently distributed over the three Py units, the third Py carries only a smaller fraction of the total charge (*q*_Py_ = 0.220 *e*), while most of the positive charge remains localized on the Py_2_^+^(a) core. Nonetheless, the third Py causes a significant reduction of the partial charge distribution in the Py_2_^+^(a) unit (0.438 and 0.342 *e*). The N─H bond of the bottom Py unit slightly contracts to 1.005 Å and shifts *ν*_NH_^f^ by +12 cm^−1^ to the blue (*ν*_NH_^f^ = 3512 cm^−1^). In contrast, the N─H bond elongates to 1.006 Å for the top Py of Py_2_^+^(a) because of the additional interaction with the π‐cloud of the third Py, leading to a redshift of −4 cm^−1^ for *ν*_NH_^f^ (*ν*_NH_^f^ = 3496 cm^−1^). The increased partial charge transfer to the third Py elongates its N─H bond by 3 mÅ, resulting in a computed redshift of −16 cm^−1^ (*ν*_NH_^f^ = 3515 cm^−1^, Table S2). The relative energy ordering of these isomers remains consistent across various computational levels (Table S3). However, the energy gap between isomers II and III becomes much larger, mainly because the obtained geometry of the π‐stacked local minimum differs from that computed at the B3LYP‐D3 level (Figures 2c and S13b). For completeness, further higher‐energy isomers of Py_3_^+^(a) and their computed IR spectra are shown in Figures S4, S5.

Interestingly, the lowest‐energy Py_3_^+^ trimer with a parallel Py_2_^+^(p) core, Py_3_^+^(p)(I), is predicted to be slightly more stable than Py_3_^+^(a)(I) by 1.9 kJ mol^−1^ (Figure 2), although for the dimer case Py_2_^+^(a) is more stable than Py_2_^+^(p) by 7.5 kJ mol^−1^ (Figure S1) [42]. However, computations at various levels (Table S3) reveal that Py_3_^+^(p)(I) is essentially isoenergetic with Py_3_^+^(a)(I). In Py_3_^+^(p)(I), the Py_2_^+^(p) core forms two nonequivalent NH…π H‐bonds to the π‐cloud of the third Py unit, which offers two equivalent binding sites (C═C bonds) to the two NH groups of Py_2_^+^(p). Therefore, the total H‐bond strength is larger than for the single H‐bond in Py_3_^+^(a)(I) (63.3 vs. 54.0 kJ mol^−1^). However, Py_3_^+^(p)(I) becomes slightly less stable than Py_3_^+^(a)(I) at higher temperatures, due to entropy‐driven destabilization arising from the strain imposed by the H‐bond network, Δ*G*(p–a) = 2.6 kJ mol^−1^. A similar phenomenon was previously observed for the dihydrated Py_2_^+^ cluster [43]. The charge transfer to the third Py unit is larger than for Py_3_^+^(a)(I) due to the double H‐bonds (0.051 vs. 0.035 *e*), resulting in a stronger elongation of the N─H bond of the third Py and thus a larger redshift for *ν*_NH_^f^ (3507 cm^−1^, Table 1). The two H‐bond donors of the Py_2_^+^(p) core both elongate by 9 (8) mÅ for the stronger (weaker) H‐bonded unit, resulting in corresponding redshifts of *ν*_NH_^b^ to 3315 and 3349 cm^−1^, respectively. Because the strengths of both individual H‐bonds in Py_3_^+^(p)(I) are weaker than that in Py_3_^+^(a)(I), the corresponding Δ*ν*_NH_^b^ shifts are smaller (Δ*ν*_NH_^b^ = −195/−150 vs. −224 cm^−1^). A further higher‐energy Py_3_^+^(p)(II) isomer, which differs only by internal rotation of the Py acceptor unit, and its computed IR spectrum are shown in Figures S4, S5.

The IRPD spectrum of Py_3_^+^ is compared in Figure 3 to the IR spectra computed for the four stable isomers shown in Figure 2. Peaks F and G are readily assigned to *ν*_NH_^b^ and *ν*_CH_ of Py_3_^+^(a)(I), considering the largely redshifted *ν*_NH_^b^ band resulting from its NH…π H‐bond. Peak E is attributed to the two overlapping *ν*_NH_^f^ modes of this isomer. The predicted *ν*_NH_^f^ splitting (Δ*ν*_NH_^f^ = 6 cm^−1^) is well within the width of band E (19 cm^−1^). A contribution of Py_3_^+^(a)(II) to the IRPD spectrum cannot be ruled out because its predicted *ν*_NH_^b^ is only 14 cm^−1^ lower than that of Py_3_^+^(a)(I) and thus cannot be resolved within the broad peak F (FWHM = 54 cm^−1^). In addition, the *ν*_NH_^f^ frequencies of Py_3_^+^(a)(II) and Py_3_^+^(a)(I) differ by less than 1 cm^−1^ and thus also overlap. The weak and broad feature F2 appearing near 3328 cm^−1^ could be attributed to the two unresolved *ν*_NH_^b^ modes of Py_3_^+^(p)(I), because the weaker H‐bonds between Py_2_^+^(p) and Py are associated with smaller predicted redshifts (Δ*ν*_NH_ = −150 and −195 cm^−1^) from *ν*_NH_^f^ of Py_2_^+^(p), consistent with the experimental redshift of −152 cm^−1^. Additionally, *ν*_NH_^f^ of the third Py of Py_3_^+^(p)(I) is predicted to be only 5 cm^−1^ lower than those of Py_3_^+^(a)(I‐II), which can also not be resolved within the broad peak E. On the other hand, the presence of Py_3_^+^(p)(II) may be excluded because its *ν*_NH_^b^ bands are predicted at even higher frequencies (Figures S4, S5). The population of Py_3_^+^(p)(I) is estimated to be less than 15% from the integrated area ratios of peaks F and F2 and the computed IR oscillator strengths. Notably, the measured integrated area ratio of peaks F/E (4.5:1) is somewhat lower than the predicted intensity ratio of *ν*_NH_^f^/*ν*_NH_^b^ of Py_3_^+^(a)(I) (5.6:1), which may suggest some minor contribution (<20%) of stacked Py_3_^+^(a)(III). In summary, Py_3_^+^(a)(I‐II) are observed to be the predominant isomers under our experimental conditions. In these clusters, the third Py solvates the CR‐stabilized Py_2_^+^(a) core via an NH…π H‐bond as predicted previously [62], rather than by π‐stacking that is observed for Bz_3_^+^ [53, 54, 55, 56, 57, 58, 59]. Our assignment for the peak at 3328 cm^−1^ is the same as given for the previously reported VUV‐IRPD spectrum of Py_3_^+^ [63]. In contrast, the given interpretation of the peaks at 3224 and 3489 cm^−1^ differs from our assignment.

The IRPD spectrum of Ar‐tagged Py_3_^+^ is better resolved than that for bare Py_3_^+^ and exhibits three distinct *ν*_NH_^f^ bands (E′, E2, E at 3504, 3494, 3489 cm^−1^), four *ν*_NH_^b^ bands (F2, F2′, F1, F at 3309, 3286, 3234, 3214 cm^−1^), and one *ν*_CH_ band (G at 3137 cm^−1^), with widths of 3–27 cm^−1^. The major peak positions and the integrated F/E peak ratio remain similar to those of Py_3_^+^, indicating that Ar‐tagging has only a minor effect on the observed structures of the Py_3_^+^ isomers, while providing a colder and better‐resolved spectrum for Py_3_^+^.

According to the calculations, Ar attaches to Py_3_^+^(a)(I‐II) in the same manner, by interacting with the N─H bond of the free Py unit of the Py_2_^+^(a) core with *D*_0_ = 7.2 kJ mol^−1^ (Figures 2e–f). In both cases, *r*_NH_ and *ν*_NH_^f^ of the H‐bonded Py solvent remain unaffected (*r*_NH_ = 1.005 Å, *ν*_NH_^f^ = 3512 cm^−1^). The *ν*_NH_^f^ modes of the Py_2_^+^(a) core associated with the weak and nonlinear H‐bonds with Ar have negligible redshifts of −1 and −2 cm^−1^ in Py_3_^+^(a)(I,II)Ar(I). Their corresponding *ν*_NH_^b^ bands predicted at 3282 and 3264 cm^−1^ exhibit small blueshifts of +6 and +2 cm^−1^ upon Ar attachment, respectively. These shifts mostly arise from slight reductions in the positive partial charges on the corresponding Py units of the Py_2_^+^ core (0.516 vs. 0.522 *e* and 0.532 vs. 0.541 *e*). Overall, Ar attachment has a minimal effect on the geometries of Py_3_^+^(a)(I‐II) and their predicted *ν*_NH_ frequencies. Therefore, higher‐energy Ar‐tagged isomers are not discussed in detail here because their Ar binding site is less certain. For completeness, their geometries and computed IR spectra are provided in Figures S6, S8. In Py_3_^+^(p)(I)Ar(I), Ar binds to the CH groups of the two Py units of the Py_2_^+^(p) core with *D*_0_ = 7.9 kJ mol^−1^ (Figure 2g). As Ar attachment has a very minor effect on the charge distribution in Py_3_^+^(p)(I) (Δ*q* ≤ 4 m*e*) and its N─H bond lengths (Δ*r*_NH_ ≤ 1 mÅ), the free and bound *ν*_NH_ band positions are barely affected (Δ*ν*_NH_ ≤ 2 cm^−1^). Further higher‐energy isomers of Py_3_^+^(p)(I)Ar and their computed IR spectra are available in Figures S7, S8.

In the IRPD spectrum of Py_3_^+^Ar, peaks F, F2, and E are better resolved compared to the spectrum of bare Py_3_^+^ due to the significantly reduced internal energy achieved by Ar‐tagging. The IRPD spectrum of Py_3_^+^Ar suggests that peak F at 3214 cm^−1^ corresponds to *ν*_NH_^b^ of Py_3_^+^(a)(II)Ar(I), while the newly resolved shoulder F1 at 3234 cm^−1^ is currently attributed to *ν*_NH_^b^ of the lower‐energy isomer, Py_3_^+^(a)(I)Ar(I). For both Py_3_^+^(a)(I‐II)Ar(I), peak E at 3489 cm^−1^ corresponds to *ν*_NH_^f^ of the Ar‐bound Py and peak E′ at 3504 cm^−1^ to *ν*_NH_^f^ of the neutral Py. Ar‐tagging resolves the shoulder F2′ at 3286 cm^−1^, indicating the presence of the Py_3_^+^(p)Ar(I) isomer by assigning F2′ and F2 to its two *ν*_NH_^b^ bands. Additionally, *ν*_NH_^f^ of this isomer is predicted midway between the two *ν*_NH_^f^ bands of Py_3_^+^(a)(I‐II)Ar(I), which corresponds well with peak E2 at 3494 cm^−1^. For the given assignment, the isomer abundances of Py_3_^+^(a)(II)(Ar)(I), Py_3_^+^(a)(I)(Ar)(I), and Py_3_^+^(p)(I)(Ar)(I) are estimated as 70%, 10%, and 20%, respectively, when considering the computed and experimental *ν*_NH_^b^ band intensities. Hence, for the trimer (*n* = 3), clusters with a parallel Py_2_^+^(p) core (20%) are observed to be more abundant compared to the dimer cluster (<15%) [42], due to the stabilizing effect of the third Py unit arising from the formation of a second NH…π H‐bond. Nonetheless, Py_3_^+^ isomers with an antiparallel Py_2_^+^(a) core clearly dominate the population in our EI plasma expansion for both bare and Ar‐tagged clusters (65 and 80%). Significantly, this assignment is in clear contrast to the recent interpretation of the IRPD spectrum of VUV‐generated Py_3_^+^ (Figure S3), in which bands F (and F1) have not been attributed to Py_3_^+^(a) clusters but to chemically bonded species [63]. Our results also suggest that the previous assignment may not account for the most abundant unreacted Py_3_^+^ isomer.

The minimum energy path on the potential energy surface for converting Py_3_^+^(a)(I) into Py_3_^+^(p)(I) by hindered internal rotation is determined using the nudged elastic band method (Figure 4a). The relative energies of *E*_e_ = 0 and −4.3 kJ mol^−1^ for Py_3_^+^(a)(I) and Py_3_^+^(p)(I) are comparable to the zero‐point corrected *E*_0_ values of 0 and −1.9 kJ mol^−1^, respectively. According to these energies, the relative energies of the antiparallel and parallel orientation of the Py_2_^+^ CR core change their order in Py_3_^+^ by attachment of the third Py (*E*_e_ = −4.3 kJ mol^−1^ for Py_3_^+^(p) vs. +8.6 kJ mol^−1^ for Py_2_^+^(p)) [42], because Py_3_^+^(p) is substantially stabilized by the additional H‐bond. The internal rotation barriers are computed as *V*_b_(a→p) = 43.8 and *V*_b_(p→a) = 48.1 kJ mol^−1^ for the forward and backward reactions, respectively, which are somewhat higher compared to those of bare Py_2_^+^ (37.7 and 29.1 kJ mol^−1^), revealing that the additional NH…π H‐bond increases the barrier height for internal rotation of Py_2_^+^ in Py_3_^+^. Overall, the additional NH…π H‐bond stabilizes the parallel dimer core configuration.

**FIGURE 4:**
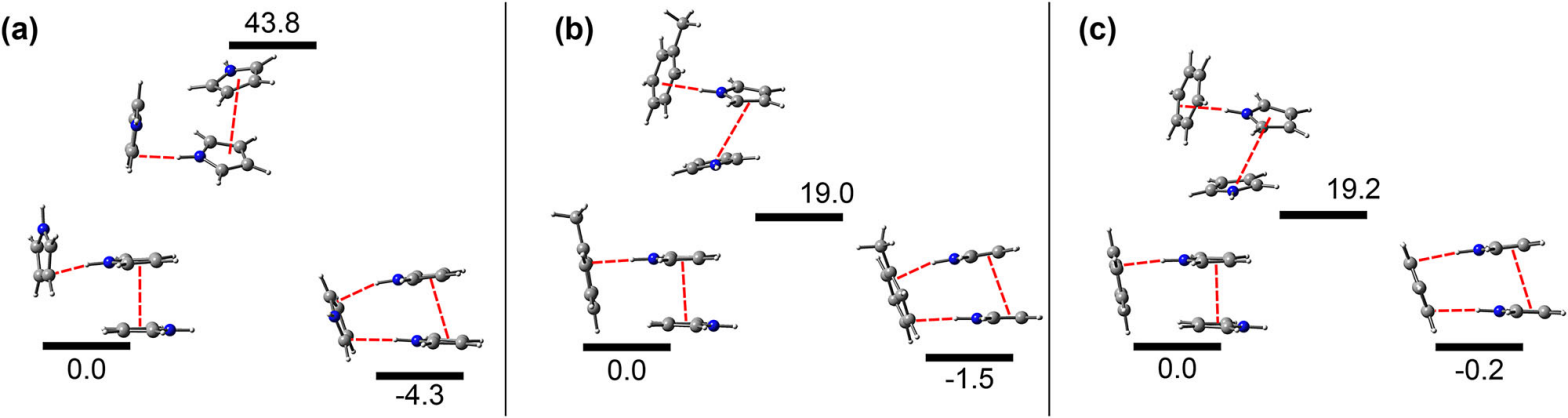
Potential energy surface (uncorrected for zero‐point vibrational energy) for the isomerization pathways (a) Py_3_^+^(a)(I) ↔ Py_3_^+^(p)(I), (b) Py_2_^+^(a)Tol(I) ↔ Py_2_^+^(p)Tol(I), and (c) Py_2_^+^(a)Bz(I) ↔ Py_2_^+^(p)Bz(I) in kJ mol^−1^ using the nudged elastic band method (B3LYP‐D3/aug‐cc‐pVTZ).

### 3.2 Py_2_^+^X

Analogous to Py_3_^+^, the IRPD spectra of both Py_2_^+^Tol and Py_2_^+^Bz recorded in the Py_2_^+^ fragment channel exhibit three major peaks E, F, and G attributed to *ν*_NH_^f^, *ν*_NH_^b^, and *ν*_CH_ bands, respectively (Figure 1). This observation suggests that Tol and Bz solvate the CR‐stabilized Py_2_^+^ core in a similar way as Py. The *ν*_NH_^b^ bands (F) of Py_2_^+^Tol and Py_2_^+^Bz observed at 3304 and 3321 cm^−1^ have smaller redshifts compared to that of Py_3_^+^ (−176 and −159 vs. −266 cm^−1^), indicating the formation of weaker NH…π H‐bonds. The presence of the intense *ν*_NH_^f^ band E suggests that Tol and Bz predominantly solvate a Py_2_^+^(a) rather than a Py_2_^+^(p) core. A nearly imperceptible shoulder F2 indicates a very small population of isomers with a Py_2_^+^(p) core. The corresponding blueshifts of the *ν*_NH_^f^ band of the Py_2_^+^(a) core in Py_2_^+^Tol and Py_2_^+^Bz (+7 and +4 cm^−1^) are smaller than that for Py_3_^+^ (+16 cm^−1^), confirming the formation of weaker H‐bonds. The *ν*_CH_ bands for Py_2_^+^Tol (3128 cm^−1^) and Py_2_^+^Bz (3131 cm^−1^) match that of Py_3_^+^ (3130 cm^−1^).

According to the calculations, the Py_2_^+^(a) core is solvated by Tol or Bz via an NH…π H‐bond, with *D*_0_ = 52.3 and 47.3 kJ mol^−1^ in Py_2_^+^(a)Tol(I) and Py_2_^+^(a)Bz(I), respectively (Figure 5). The N─H donor bonds are less elongated than in Py_3_^+^(a) (7 and 6 vs. 12 mÅ), resulting in smaller computed redshifts of *ν*_NH_^b^ (−121 and −114 vs. −224 cm^−1^), in line with the experimental spectra. This result indicates that the NH…π H‐bonds between Py_2_^+^(a) and Tol and Bz are indeed weaker compared to that to Py in Py_3_^+^(a)(I), whereby the H‐bond to Bz is weaker than to Tol. In the homotrimer, the H‐bond between Py_2_^+^(a) and Py is governed by charge‐dipole interaction, while it arises mainly from the charge‐quadrupole interaction in the heterotrimers because of the significantly smaller or vanishing dipole moment of Tol or Bz (*μ* = 1.84, 0.36, 0 D for Py, Tol, Bz) [81]. The NCI analysis reproduces this trend of the strength of the H‐bonds for Tol and Py (ρ* = −0.014 vs. −0.020 a.u.), while it appears to be comparable for both Bz and Tol (Figure S9). However, *D*_0_ of Py_2_^+^(a)Tol(I) is larger than that of Py_2_^+^(a)Bz(I) due to the larger polarizability of Tol (11.861 vs. 9.996 Å^3^) [82]. Tol and Bz also cause a smaller perturbation of the CR of the solvated Py_2_^+^(a) core than Py because of their weaker H‐bonds. Indeed, the free Py unit of Py_2_^+^(a)Tol/Bz(I) carries more positive charge compared to Py_3_^+^(a)(I) and less compared to unperturbed Py_2_^+^(a) (0.442 < 0.455/0.455 < 0.5 *e*), leading to a smaller blueshift of +5 cm^−1^ for *ν*_NH_^f^ of the Py_2_^+^(a) core. For completeness, further higher‐energy Py_2_^+^(a)Tol isomers and their computed IR spectra are shown in Figures S10, S11.

**FIGURE 5:**
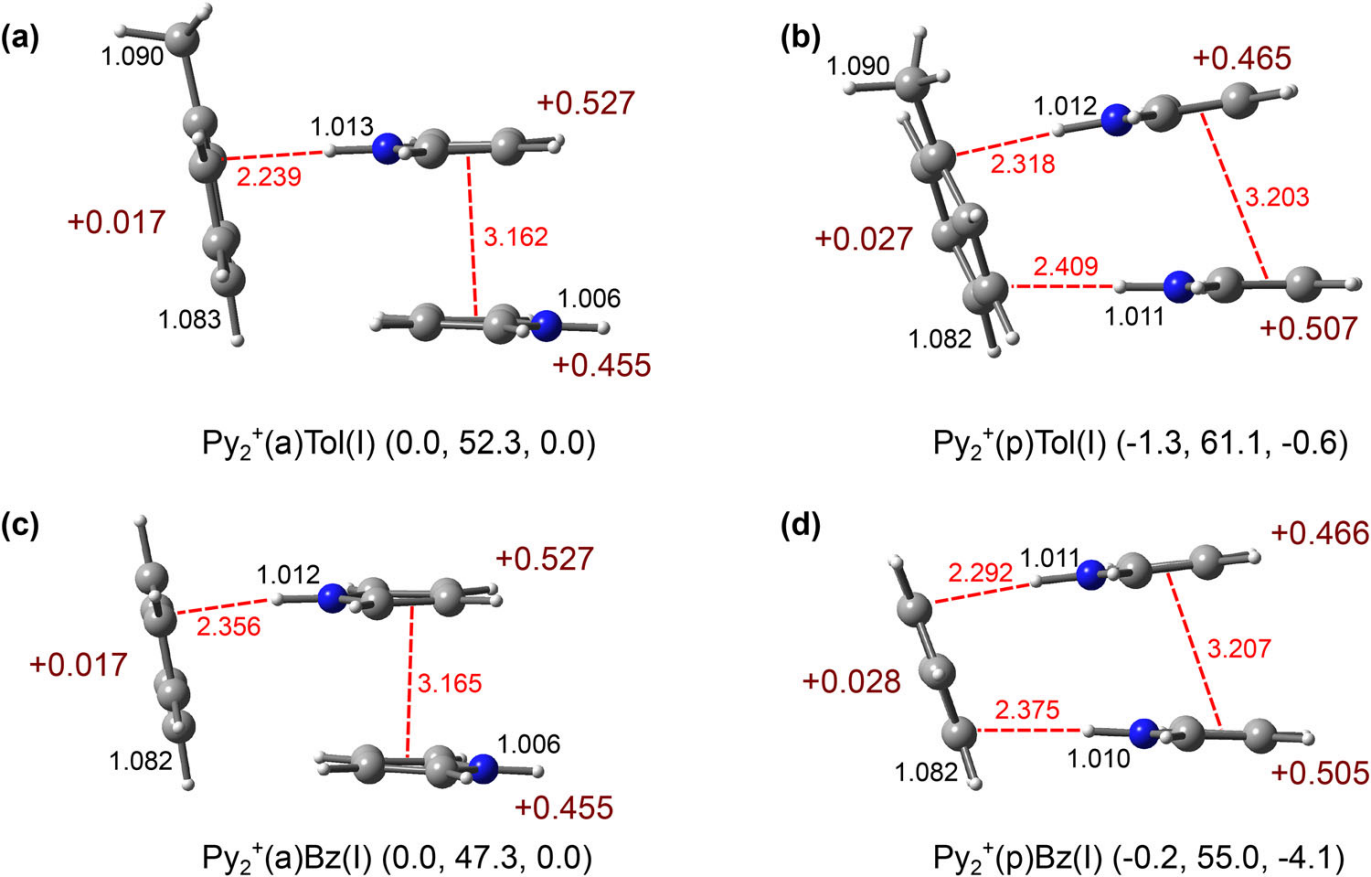
Structures of (a) Py_2_^+^(a)Tol(I), (b) Py_2_^+^(p)Tol(I), (c) Py_2_^+^(a)Bz(I), and (d) Py_2_^+^(p)Bz(I) obtained at the B3LYP‐D3/aug‐cc‐pVTZ level. Selected intramolecular and intermolecular bond lengths (in Å) are given in black and red, respectively. Values in dark red indicate NBO charges (in units of *e*). Energies in parentheses are relative energy referenced to Py_2_^+^(a)Tol(I) for (a,b) and Py_2_^+^(a)Bz(I) for (c,d), dissociation energy of the most weakly bonded ligand, and relative Gibbs free energy at 298 K (*E*_0_, *D*_0_, *G*_0_ in kJ mol^−1^). *D*_0_ accounts for the loss of Tol or Bz.

Tol and Bz solvate the parallel Py_2_^+^(p) core via two NH…π bonds (*D*_0_ = 61.1 and 55.0 kJ/mol) with a binding motif similar to Py_3_^+^(p)(I) (Figure 5). Because the individual intermolecular H‐bonds in Py_2_^+^(p)Tol(I) and Py_2_^+^(p)Bz(I) are weaker than in Py_3_^+^(p)(I) (*R*_NH…π  _= 2.318/2.409 and 2.292/2.375 vs. 2.242/2.347 Å), the intramolecular N─H donor bonds of the Py_2_^+^(p) core are less elongated. Moreover, the weaker H‐bonds induce a smaller perturbation of the CR in Py_2_^+^(p) and thus a reduced elongation of the N─H bonds. As a result, the *ν*_NH_^b^ redshifts are predicted to be smaller (Δ*ν*_NH_^b^ = −128/−106 and −112/−95 vs. −195/−150 cm^−1^). The *E*_0_ values of the antiparallel and parallel isomers for Py_2_^+^Tol and Py_2_^+^Bz are predicted to be almost isoenergetic, while the *D*_0_ values clearly remain in favor of the parallel isomers across various computational levels (Table S3).

The IRPD spectrum of Py_2_^+^Tol in Figure 6 displays three major peaks E, F, and G at 3487, 3304, and 3128 cm^−1^ with widths of 9–29 cm^−1^. Peak E is readily assigned to *ν*_NH_^f^ of Py_2_^+^(a)Tol(I). The measured blueshift caused by the perturbation of the CR is well reproduced by our computational results (Δ*ν*_NH_^f^ = +7 vs. +5 cm^−1^). Peak F is attributed to the corresponding *ν*_NH_^b^ of Py_2_^+^(a)Tol(I), which is less redshifted compared to the IRPD spectrum of Py_3_^+^, again consistent with our computational results. Peak G is assigned to *ν*_CH_ of Py_2_^+^(a)Tol(I). Unlike in the IRPD spectrum of Py_3_^+^, the contributions of Py_2_^+^(p)Tol with a parallel Py_2_^+^(p) dimer core are less evident, because the splitting between its two *ν*_NH_^b^ modes lies close to the width of peak F (33 vs. 29 cm^−1^). Moreover, one of them is predicted at the same position as *ν*_NH_^b^ of Py_2_^+^(a)Tol(I). In any case, the population of Py_2_^+^(p)Tol(I) is estimated to be less than 5% from the signal‐to‐noise ratio of peak F, which is significantly less compared to that for Py_3_^+^(p)Ar (20%). The area ratio of peaks F and E is estimated to be similar to the IR oscillator strength ratio of *ν*_NH_^b^ and *ν*_NH_^f^ of Py_2_^+^(a)Tol(I) (5.4:1 vs. 4.8:1), indicating that the major contribution to the IRPD spectrum arises indeed from the NH…π H‐bonded Py_2_^+^(a)Tol(I) isomer, while ruling out a significant contribution of substantially less stable π‐stacked trimers shown in Figures S10, S11. Interestingly, the IRPD spectrum of Py_2_^+^Tol recorded in the Py^+^Tol channel (Figure S12) reveals the formation of other isomers, in which Py solvates the NH…π H‐bonded Py^+^Tol dimer core as revealed from the strongly redshifted *ν*_NH_^b^ band [50]. The absence of *ν*_NH_^f^ in the IRPD spectrum of these isomers suggests the lack of the CR interaction. Therefore, they are not considered in detail further here.

**FIGURE 6:**
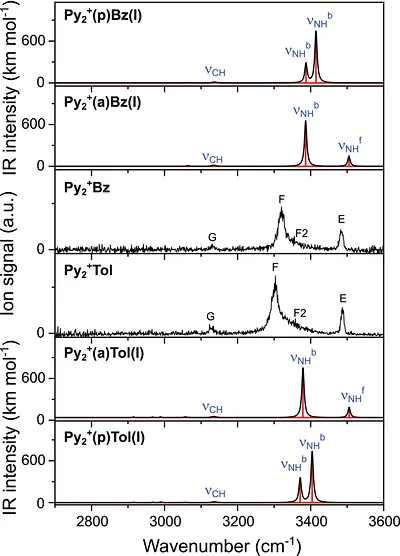
IRPD spectra of Py_2_^+^Tol and Py_2_^+^Bz recorded in the CH and NH stretch range (in the Py_2_^+^ fragment channel) compared to linear IR absorption spectra calculated for Py_2_^+^(a/p)Tol(I) and Py_2_^+^(a/p)Bz(I) at the B3LYP‐D3/aug‐cc‐pVTZ level (Tables 1 and S1).

Similar to Py_2_^+^Tol, the IRPD spectrum of Py_2_^+^Bz in Figure 6 also exhibits three major peaks E, F, and G at 3484, 3321, and 3131 cm^−1^ with widths of 10–23 cm^−1^. They are assigned to *ν*_NH_^f^, *ν*_NH_^b^, and *ν*_CH_ of Py_2_^+^(a)Bz(I), respectively. Peak F is blueshifted by +17 cm^−1^ compared to that of Py_2_^+^Tol, in line with our prediction of Δ*ν*_NH_^b^ = +7 cm^−1^. In addition, peak E is blueshifted by +4 cm^−1^ compared to *ν*_NH_^f^ of Py_2_^+^ (3484 vs. 3480 cm^−1^) and redshifted by −3 and −12 cm^−1^ from *ν*_NH_^f^ of Py_2_^+^Tol and Py_3_^+^ (3484 vs. 3487 vs. 3496 cm^−1^). This trend is in line with the perturbation of the charge distribution of Py_2_^+^(a) by the aromatic ligands with different H‐bond strengths. Similar to Py_2_^+^Tol, the contribution of Py_2_^+^(p)Bz with a parallel Py_2_^+^(p) dimer core is not clear. The population of Py_2_^+^(p)Bz(I) is estimated to be less than 10% from the signal‐to‐noise ratio of peak F.

The minimum energy paths on the potential energy surfaces for converting Py_2_^+^(a)X(I) into Py_2_^+^(p)X(I) for X = Tol/Bz by hindered internal rotation are shown in Figure 4b,c. Due to the weaker H‐bonds, the stabilization of the trimer with a parallel core by the two H‐bonds to the solvent molecule is reduced to Δ*E*_e_ = −1.5 and −0.2 kJ mol^−1^ for X = Tol and Bz. The internal rotation barriers are also reduced compared to Py_3_^+^ and predicted as *V*_b_(a→p) = 19.0 and *V*_b_(p→a) = 20.5 kJ mol^−1^ for Py_2_^+^Tol and *V*_b_(a→p) = 19.2 and *V*_b_(p→a) = 19.4 kJ mol^−1^ for Py_2_^+^Bz for the forward and backward pathways, respectively. The hindered rotational barriers are similar for Py_2_^+^Tol and Py_2_^+^Bz due to the similar NH…π H‐bond strengths, while these barriers are significantly lowered compared to those of Py_3_^+^ and bare Py_2_^+^ because of overall weaker interactions.

### 3.3 The CR in Trimers

In this section, we consider the properties of the CR in the Py_3_^+^ and Py_2_^+^X homo‐ and heterotrimers in more detail. In Py_3_^+^, the third Py solvates the CR‐stabilized Py_2_^+^(a) core via an NH…π H‐bond rather than forming a stacked structure and sharing the positive charge throughout the three rings (Figure 2). Py_3_^+^(a) and Py_3_^+^(p) isomers are nearly isoenergetic concerning their *E*_0_ values, while Py_3_^+^(p) is clearly more stable than Py_3_^+^(a) regarding the *D*_0_ values due to the formation of two H‐bonds. However, experimentally, Py_3_^+^(a) isomers with a single H‐bond are predominantly observed. This apparent discrepancy may be explained by the production mechanism for Py_3_^+^ in our EI plasma expansion via a bottom‐up cluster growth. Because Py_2_^+^(a) has the strongest CR and contributes to at least 80% of the Py_2_^+^ population under our experimental conditions [42], Py_3_^+^(a) is generated with high abundance as well. Moreover, when including entropy, the more flexible Py_3_^+^(a) structure becomes more stable than the more rigid Py_3_^+^(p) isomer. The fully π‐stacked Py_3_^+^(a)(III) isomer is predicted to be less stable than H‐bonded Py_3_^+^(a) by 5.0 kJ mol^−1^ because of poorer molecular orbital overlap caused by the third Py being tilted away from the π–π axis of Py_2_^+^(a). In fact, the Py_3_^+^(a)(III) isomer obtained at the CAM‐B3LYP‐D3 level (Figure S13b) has two equivalent Py ligands coordinated below and above the central Py^+^ unit at a somewhat larger distance (*R* = 3.244 Å), exhibiting a fully π‐stacked structure mostly bonded by long‐range van der Waals forces (electrostatic, induction, dispersion) rather than by a chemical CR force. Although this stacked configuration would in principle allow for efficient molecular orbital overlap (at short range), this stacked isomer is predicted to be significantly less stable (*E*_0_ = 19.9 kJ mol^−1^) compared to H‐bonded Py_3_^+^(a)(I) (Figure S13). This reduced stability arises from the weakening or removal of the CR when the positive charge is delocalized over three rings (+0.21, +0.58, +0.21 *e*) instead of two. Thus, the solvated dimer core, in which the CR is the strongest, emerges as the most stable motif for Py_3_^+^ rather than the stacked motif. In H‐bonded Py_3_^+^(a), the third Py ligand has only a small effect on the CR of the Py_2_^+^ core, similar to the case of microhydration in NH…O H‐bonded Py_2_^+^(H_2_O)_*n*_ clusters [43]. From the experimental results, the π‐stacked Py_3_^+^(a)(III) isomer provides at most a minor contribution to the IRPD spectra of Py_3_^+^(Ar). However, its presence cannot be completely ruled out from the measured IRPD spectrum of Py_3_^+^ due to its broad and unresolved band E, which prevents an unambiguous assignment of the isomer contributions in this spectral range. In the colder and thus better resolved spectrum of Py_3_^+^Ar, it becomes clearer that the H‐bonded isomers are dominant. Its IRPD spectrum reveals that the increased abundance of Py_3_^+^(p) compared to Py_2_^+^(p) observed in the IRPD spectrum of Py_2_^+^ is due to the enhanced stabilization of the parallel dimer core by the two H‐bonds in Py_3_^+^(p). Additionally, the NH…π H‐bond increases the hindered rotation barrier (*V*_b_), providing a favorable condition for kinetic trapping of Py_3_^+^(p) in its potential well. The estimated relative abundance of Py_3_^+^(p)Ar (20%) is comparable to the abundance of Py_2_^+^(p) (<15%) [42], suggesting that Py_3_^+^(p) is directly grown from Py_2_^+^(p).

The Py_2_^+^Tol and Py_2_^+^Bz heterotrimers have essentially the same H‐bonded structural motifs as the Py_3_^+^ homotrimer, and thus Tol or Bz solvate the CR‐stabilized Py_2_^+^(a) core. However, the NH…π H‐bond is weaker for Py_2_^+^Tol/Bz compared to that of Py_3_^+^ because the NH…π interaction mostly arises from charge‐quadrupole interaction in Py_2_^+^Tol/Bz, whereas it is dominated by charge‐dipole interaction in Py_3_^+^. Thus, solvation of Py_2_^+^(a) by Tol and Bz causes an even smaller perturbation of its CR.

Our previous studies have established a linear correlation between *ν*_NH_^f^ of Py_*n*_^+^ (*n* = 0‐2) and the partial charge on the free Py unit (*q*_Py_), indicated as a dashed line in Figure 7a [20, 42]. Moreover, solvation of Py_2_^+^ by nonpolar and polar ligands L affects the CR by breaking the symmetry and thus modifies its positive charge distribution in Py_2_^+^L_*n*_ (L_*n*_ = Ar, N_2_, H_2_O, (H_2_O)_2_) [42, 43]. The *ν*_NH_^f^ frequencies of Py_2_^+^L_*n*_ show small but systematic shifts depending on *q*_Py_ that correspond well with the established linear *ν*_NH_^f^(*q*_Py_) correlation [20]. Figure 7a shows that this correlation also holds for the measured *ν*_NH_^f^ frequencies and the NBO charges of the free Py unit of the Py_2_^+^X and Py_3_^+^ trimers, along with Py_2_^+^(H_2_O)_*n* = 1‐2_, computed at the CAM‐B3LYP‐D3/aug‐cc‐pVTZ level. A similar *ν*_NH_^f^(*q*_Py_) correlation is obtained at the B3LYP‐D3/aug‐cc‐pVTZ level (Figure S14). The *ν*_NH_^f^ frequency of the Py_2_^+^ core is systematically blueshifted to 3489, 3487, and 3484 cm^−1^ for Py_3_^+^, Py_2_^+^Tol, and Py_2_^+^Bz, while *q*_Py_ is computed as 0.413, 0.427, and 0.429 *e*, respectively. The positive charge distribution in the CR‐stabilized Py_2_^+^ core and the observed blueshifts in *ν*_NH_^f^ change with the strength of the NH…π H‐bond. Figures 7b,c (CAM‐B3LYP‐D3) and S14b,c (B3LYP‐D3) display the correlations between *D*_e_ and both the observed blueshift in *ν*_NH_^f^ and *q*_Py_. Here, dispersion contributions to *D*_e_ are represented by a dotted pattern and the electrostatic (and induction) contributions are highlighted in dark red. The changes in *q*_Py_ and *ν*_NH_^f^ of Py_2_^+^X mostly arise from the electrostatic contributions to the interaction rather than the total binding energy. Given the comparable electrostatic (and induction) interaction of the NH…π H‐bonds, Tol and Bz are predicted to have comparable effects on *q*_Py_, whereas the impact is larger in Py_3_^+^ and Py_2_^+^H_2_O due to the stronger charge‐dipole forces.

**FIGURE 7:**
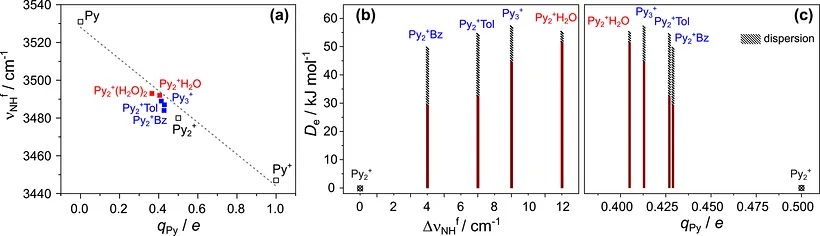
(a) Observed *ν*_NH_^f^ values for Py, Py^+^, and Py_2_^+^ (open symbols) and Py_2_^+^(H_2_O)_1,2_, Py_3_^+^, Py_2_^+^Tol, and Py_2_^+^Bz (filled symbols) plotted as a function of the NBO charge on the free Py unit (*q*_Py_) computed at the CAM‐B3LYP‐D3/aug‐cc‐pVTZ level. The dashed line represents a linear fit based on data for Py, Py^+^, and Py_2_^+^. (b,c) Computed dissociation energies (*D*_e_) of the corresponding H‐bonds (uncorrected for zero‐point vibrational energy) with and without dispersion contributions as a function of (b) the observed Δ*ν*_NH_^f^ blueshifts and (c) the computed NBO charges on the free Py unit for Py_2_^+^Bz, Py_2_^+^Tol, Py_3_^+^, and Py_2_^+^H_2_O trimers (Table S6).

### 3.4 Correlation of *ν*_NH_^b^ With *D*_e_ of H‐Bond

In H‐bonded or proton‐bound clusters, the proton donor stretch frequency (*ν*_NH_^b^) experiences a redshift which correlates with the strength of the H‐bond (*D*_0_) [83, 84, 85]. Previously, we demonstrated a linear correlation between the computed *D*_e_ values and the redshifts Δ*ν*_NH_^b^ observed for various NH…π H‐bonded dimers, such as Py^+^Bz and Py^+^Tol [50]. This relationship is extended here to Py_3_^+^, Py_2_^+^Tol, and Py_2_^+^Bz, where the NH…π H‐bonds are formed between the Py_2_^+^(a) core and the Py, Tol, and Bz ligands, with the positive charge delocalized over Py_2_^+^(a) (Figure 8). The Δ*ν*_NH_^b^ shifts of the Py^+^Tol, Py^+^Bz, and Py^+^H_2_O dimers are referenced to *ν*_NH_^f^ of Py^+^ (3447 cm^−1^) [44], while the Δ*ν*_NH_^b^ shifts of Py_3_^+^, Py_2_^+^Tol, Py_2_^+^Bz, and Py_2_^+^H_2_O are referenced to *ν*_NH_^f^ of Py_2_^+^ (3480 cm^−1^) [20, 42]. The extended *D*_e_(*ν*_NH_^b^) correlation exhibits two distinct interaction trends. The trend with the larger slope (solid line) includes Py_2_^+^Bz, Py_2_^+^Tol, Py^+^Bz, and Py^+^Tol, for which Δ*ν*_NH_^b^ (−159, −176, −264, −297 cm^−1^) are relatively small despite their comparably large *D*_e_ (49.9, 54.7, 62.4, 67.9 kJ mol^−1^). In fact, these clusters follow the same trend, suggesting a similar interaction origin arising primarily from charge‐quadrupole, induction, and dispersion contributions. In contrast, the trend with a smaller slope (dotted line) comprises the Py_2_^+^H_2_O, Py_3_^+^, and Py^+^H_2_O clusters, in which charge‐dipole interactions are significant, and which exhibit larger Δ*ν*_NH_^b^ shifts (−252, −266, −502 cm^−1^) for comparable *D*_e_ (55.5, 57.4, 74.3 kJ mol^−1^). In conclusion, this correlation highlights that the nature of electrostatic and induction contributions strongly influences the *ν*_NH_^b^ shift, whereas *D*_e_ reflects all forces, including dispersion.

**FIGURE 8:**
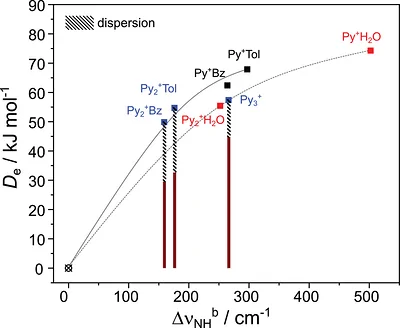
Correlation of observed Δ*ν*_NH_^b^ frequency shifts (absolute values) of Py_3_^+^, Py_2_^+^Tol, and Py_2_^+^Bz with computed dissociation energies (*D*_e_) of the corresponding H‐bonds (uncorrected for zero‐point vibrational energy) in comparison to that of Py^+^Bz, Py^+^Tol, and Py^+^H_2_O computed at the CAM‐B3LYP‐D3/aug‐cc‐pVTZ level. The gray lines serve to guide the eye.

## 4 Conclusions

In summary, we investigate herein the influence of the CR interaction on the binding motifs of aromatic homo‐ and heterotrimer radical cations utilizing IR spectroscopy of mass‐selected Py_3_^+^, Py_2_^+^Tol, and Py_2_^+^Bz clusters. We also demonstrate that the high‐pressure EI plasma expansion source, combined with Ar‐tagging, is clearly superior to VUV ionization of neutral clusters for generating cold and unreacted Py_3_^+^ clusters and its heterodimers, without triggering intracluster chemical reactions and producing contaminating isobaric ions with chemically bonded species. Systematic shifts observed in the *ν*_NH_ stretch band of the Py units provide detailed information about structures, binding motifs, and strengths of the corresponding intermolecular interactions present in the observed isomers. The analysis is supported by the complementary DFT calculations and provides detailed insight into the CR interaction, in particular the charge distribution and molecular orbital overlap in the trimer cations. In the most stable Py_3_^+^ homotrimer, the positive charge is mostly delocalized within the Py_2_^+^ core rather than being distributed over all three rings. The third Py ligand simply solvates this CR‐stabilized Py_2_^+^ unit via an NH…π H‐bond, arising from the acidic NH group of Py. Overall, this binding motif differs drastically from that observed for related bare aromatic trimer cations, such of the Bz_3_^+^, in which the third molecule solvates the CR dimer core by π‐stacking. An analogous binding motif as deduced for Py_3_^+^ is retained in the most stable Py_2_^+^Tol and Py_2_^+^Bz heterotrimers. The positive charge is again localized on the Py_2_^+^ core due to the higher ionization energies of Tol and Bz when compared to Py (8.828 and 9.244 vs. 8.207 eV). Despite their relatively high binding energies (∼0.5 eV), the NH…π H‐bonds formed between the neutral aromatic ligand and the Py_2_^+^ core have only a minor effect on the CR interaction, highlighting the strong intrinsic stability of this interaction type. The new experimental and computational data obtained for the homo‐ and heterotrimers allow us to further confirm the linear *q*_Py_‐*ν*_NH_^f^ correlation derived in our earlier study [20]. Furthermore, the obtained correlation between the *ν*_NH_^b^ redshifts of Py_*n*_^+^ (*n* = 1 or 2) and the computed binding energies of the NH…π interactions reflects the predominantly electrostatic origin of the corresponding H‐bonds. Significantly, we characterize herein the CR interaction in aromatic trimer cations in detail for the first time, utilizing high‐resolution IR spectroscopy, demonstrating that the applied IRPD approach is a powerful tool for exploring the evolution of the CR interaction in aromatic clusters larger than dimers in their ground electronic state. We are currently expanding this study to even larger Py*_n_* ^+^ clusters to explore the evolution of the aromatic CR interaction as a function of cluster size.

## Supporting Information

LID spectra, structures, measured and computed harmonic IR spectra, energies, NCI plots of isomers of Py, Py_2_^+^, Py_3_^+^(Ar), Py_2_^+^Tol, and Py_2_^+^Bz, and comparison of EI‐IRPD and VUV‐IRPD spectra of Py_3_^+^.

## Conflicts of Interest

The authors declare no conflicts of interest.

## Supporting information




**Supporting File**: chem71324‐sup‐0001‐SuppMat.pdf

## Data Availability

The data that support the findings of this study are available from the corresponding author upon reasonable request.
