# Supplementary material for: Charge Resonance Interaction in Aromatic Trimer Radical Cations Revealed by IR Spectroscopy: The Case of Pyrrole Homo‐ and Heterotrimers
Source: Chemistry. 2026 Jul 1;32(38):e71324. doi: 10.1002/chem.71324 (PMC13660490; doi:10.1002/chem.71324)
Supplement: Supplementary file 1 [file CHEM-32-e71324-s001.pdf]

Supporting Information

Charge Resonance Interaction in Aromatic Trimer Radical Cations Revealed by IR Spectroscopy: The Case of Pyrrole Homo- and Heterotrimers

Dashjargal Arildii and Otto Dopfer*

Institut für Physik und Astronomie, Technische Universität Berlin, Hardenbergstrasse 36, 10623 Berlin, Germany

* Corresponding author: dopfer@physik.tu-berlin.de

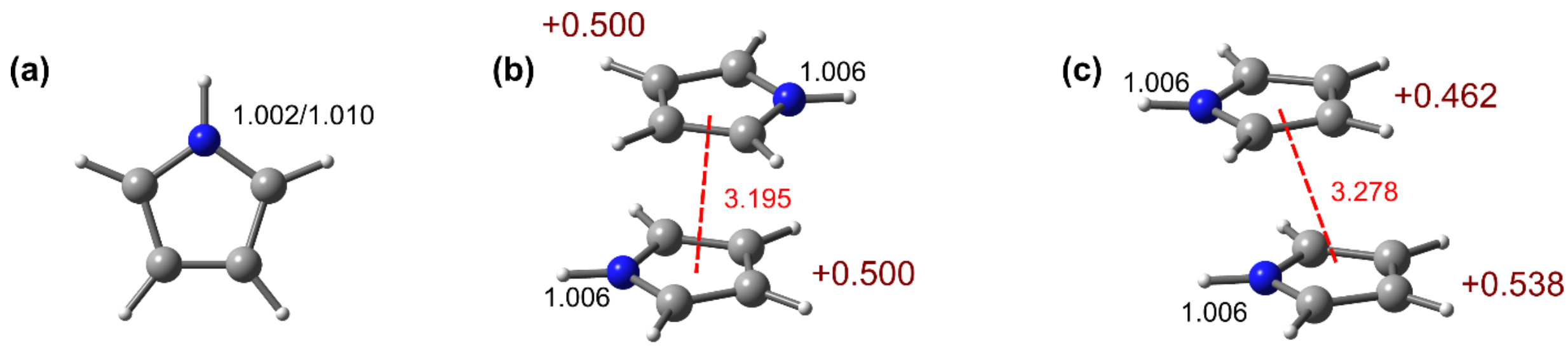

Py (C_{2v}) / Py$^+$(C_{2v}) Py$_2^+$(a) (C_{2h}) (0, 107.4) Py$_2^+$(p) (C_s) (7.5, 99.9)

Figure S1. Structures of (a) Py$^{(+)}$, (b) Py$_2^+$(a), and (c) Py$_2^+$(p) obtained at the B3LYP-D3/aug-cc-pVTZ level. Selected intra- and intermolecular bond lengths (in Å) are given in black and red, respectively. Values in dark red indicate NBO charges (in units of *e*). The relative and dissociation energies of Py$_2^+$(a) and Py$_2^+$(p) (E_0 and D_0, in parentheses) are given in kJ mol^{-1}.

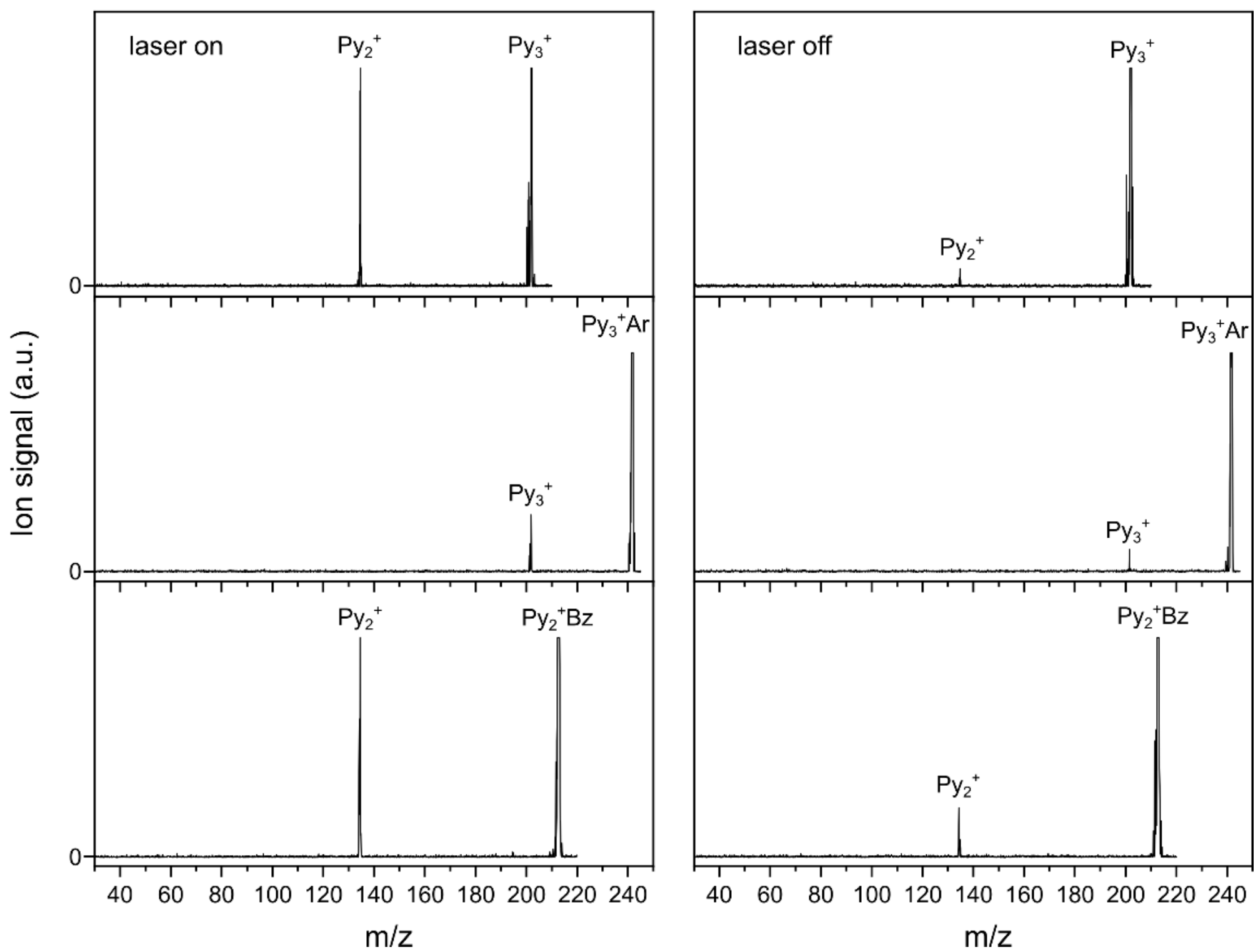

Figure S2. Laser-induced dissociation spectra of Py_3^+(Ar) and Py_2^+Bz are measured at 3217 cm^{-1} and 3342 cm^{-1}, respectively.

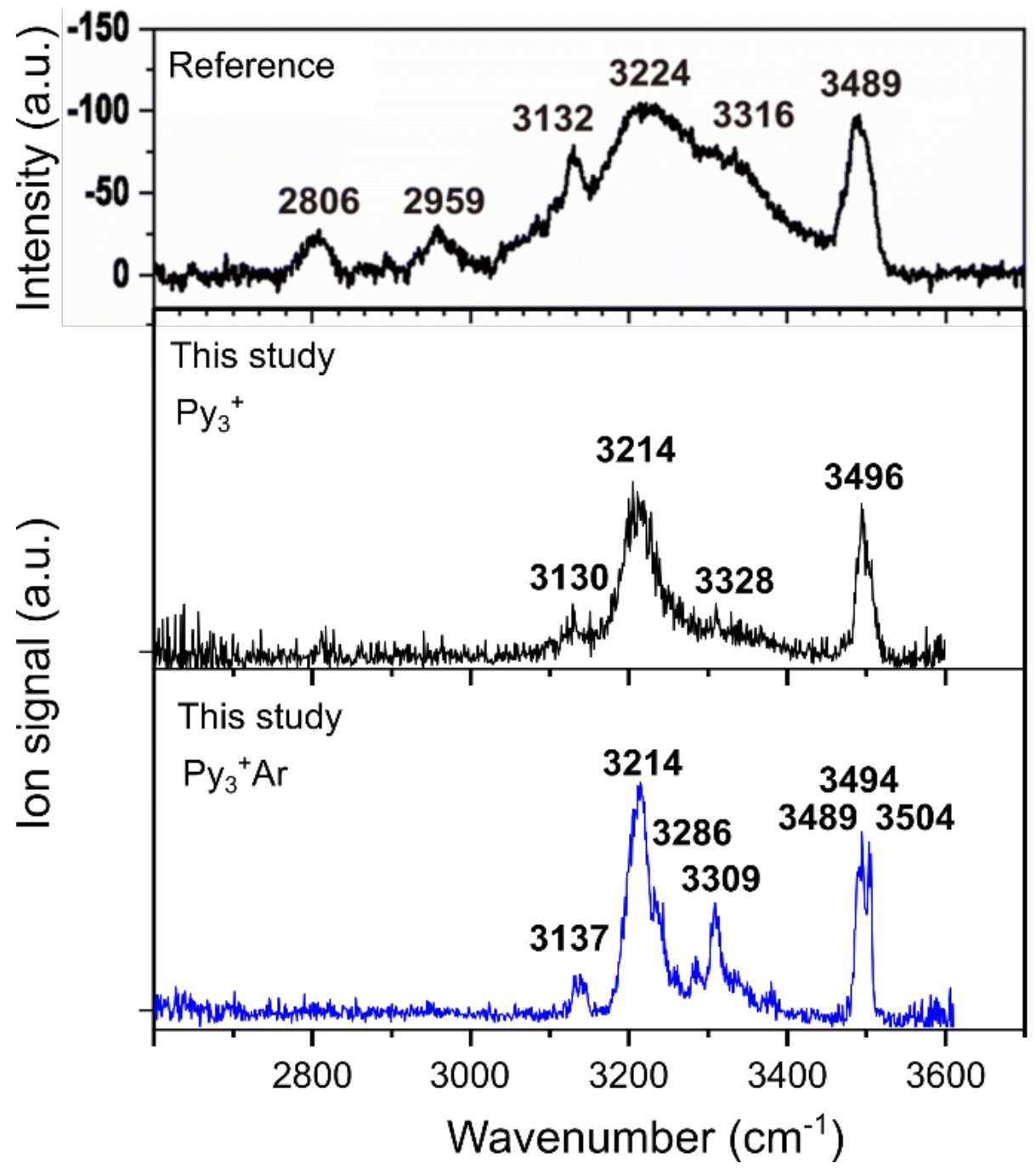

Figure S3. IRPD spectra of Py_3^+(Ar) reported in this study are compared with the previously reported IR spectrum of VUV-ionized Py_3^+.[1]

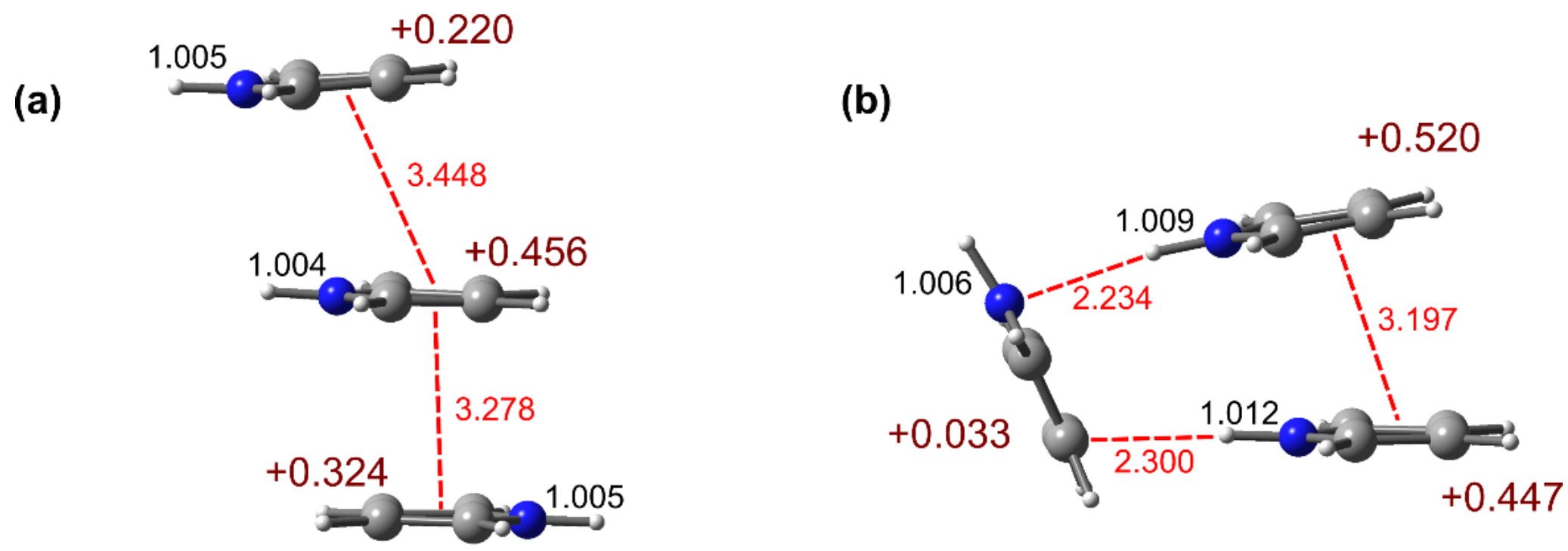

Py_3^+(a)(IV) (9.6, 44.4, 12.7) Py_3^+(p)(II) (0.7, 60.8, 2.9)

Figure S4. Structures of higher energy Py_3^+(a) and Py_3^+(p) obtained at the B3LYP-D3/aug-cc-pVTZ level. Selected intra- and intermolecular bond lengths (in Å) are given in black and red, respectively. Values in dark red indicate NBO charges (in units of *e*). Energies in parentheses are the relative energy, the dissociation energy of the most weakly bonded ligand, and Gibbs free energy at 298 K (E_0, D_0, and G_0 in kJ mol^{-1}).

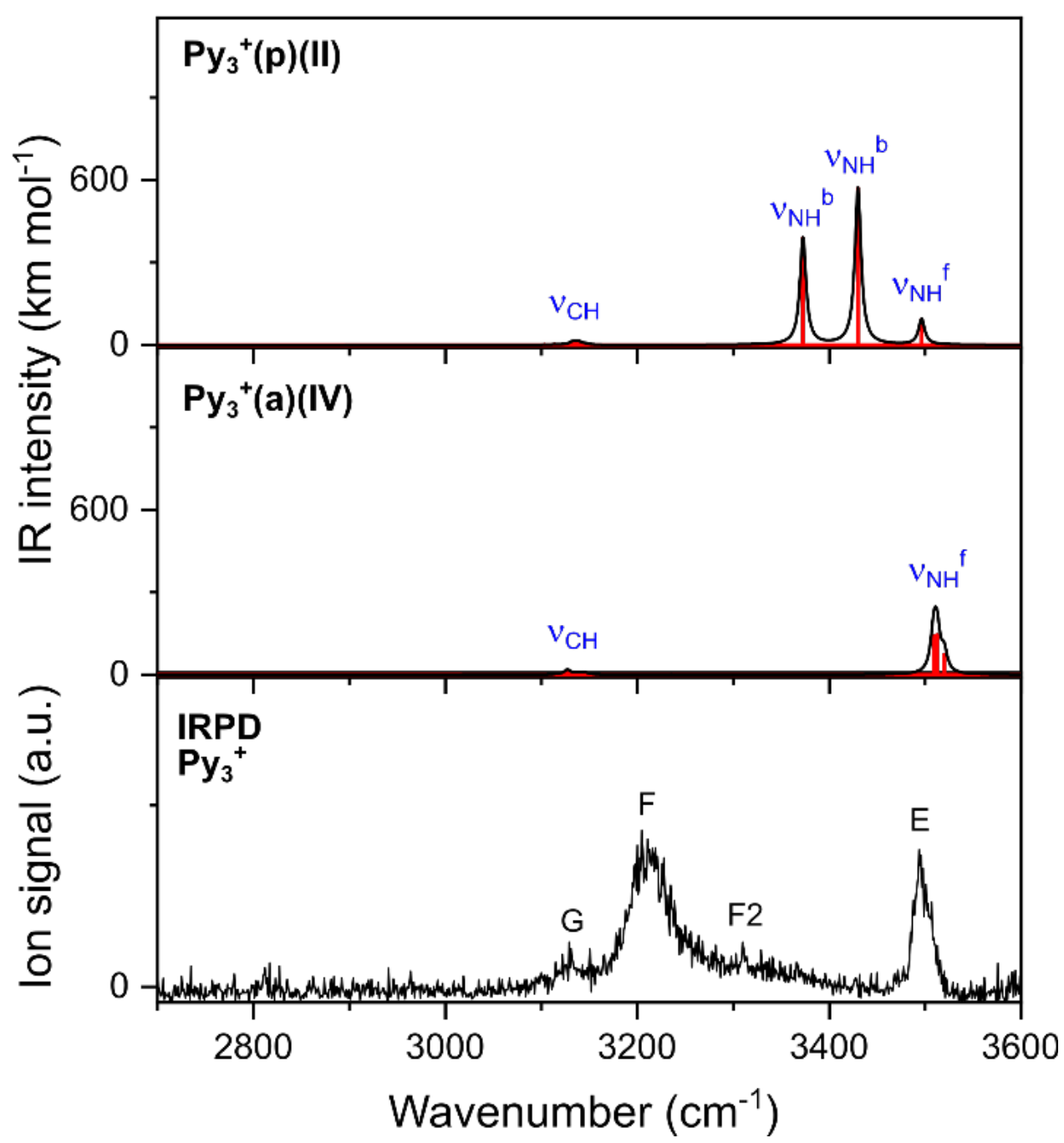

Figure S5. IRPD spectrum of Py_3^+ recorded in the CH and NH stretch range compared to linear IR absorption spectra calculated for Py_3^+(a)(IV) and Py_3^+(p)(II) at the B3LYP-D3/aug-cc-pVTZ level (Table S2).

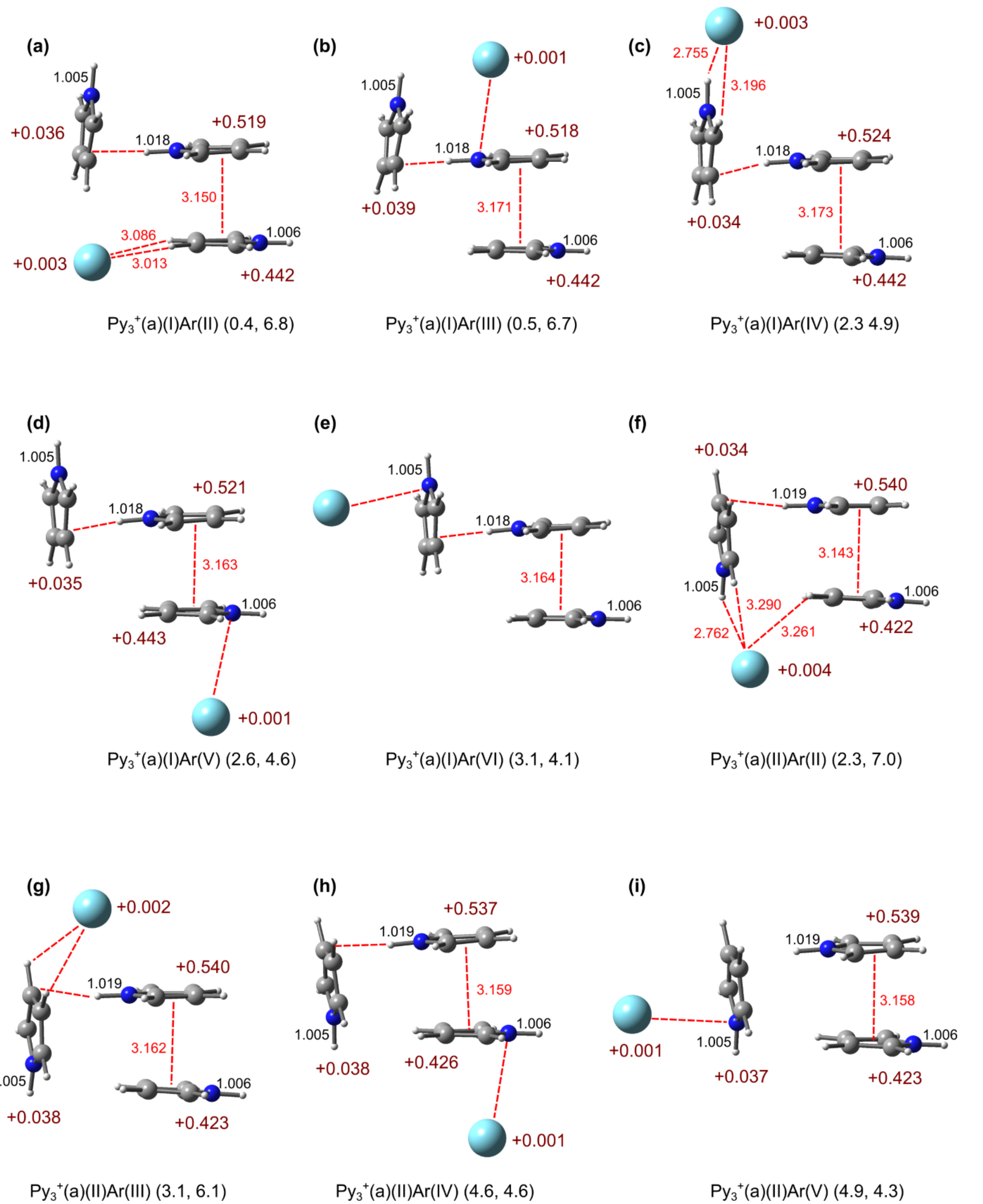

Figure S6. Structures of (a-e) Py_3^+(a)(I)Ar(II-VI) and (f-i) Py_3^+(a)(II)Ar(I-V) obtained at the B3LYP-D3/aug-cc-pVTZ level. Selected intra- and intermolecular bond lengths (in Å) are given in black and red, respectively. Values in dark red indicate NBO charges (in units of *e*). Energies in parentheses are the relative energy and the dissociation energy of the most weakly bonded ligand (E_0 and D_0 in kJ mol^{-1}).

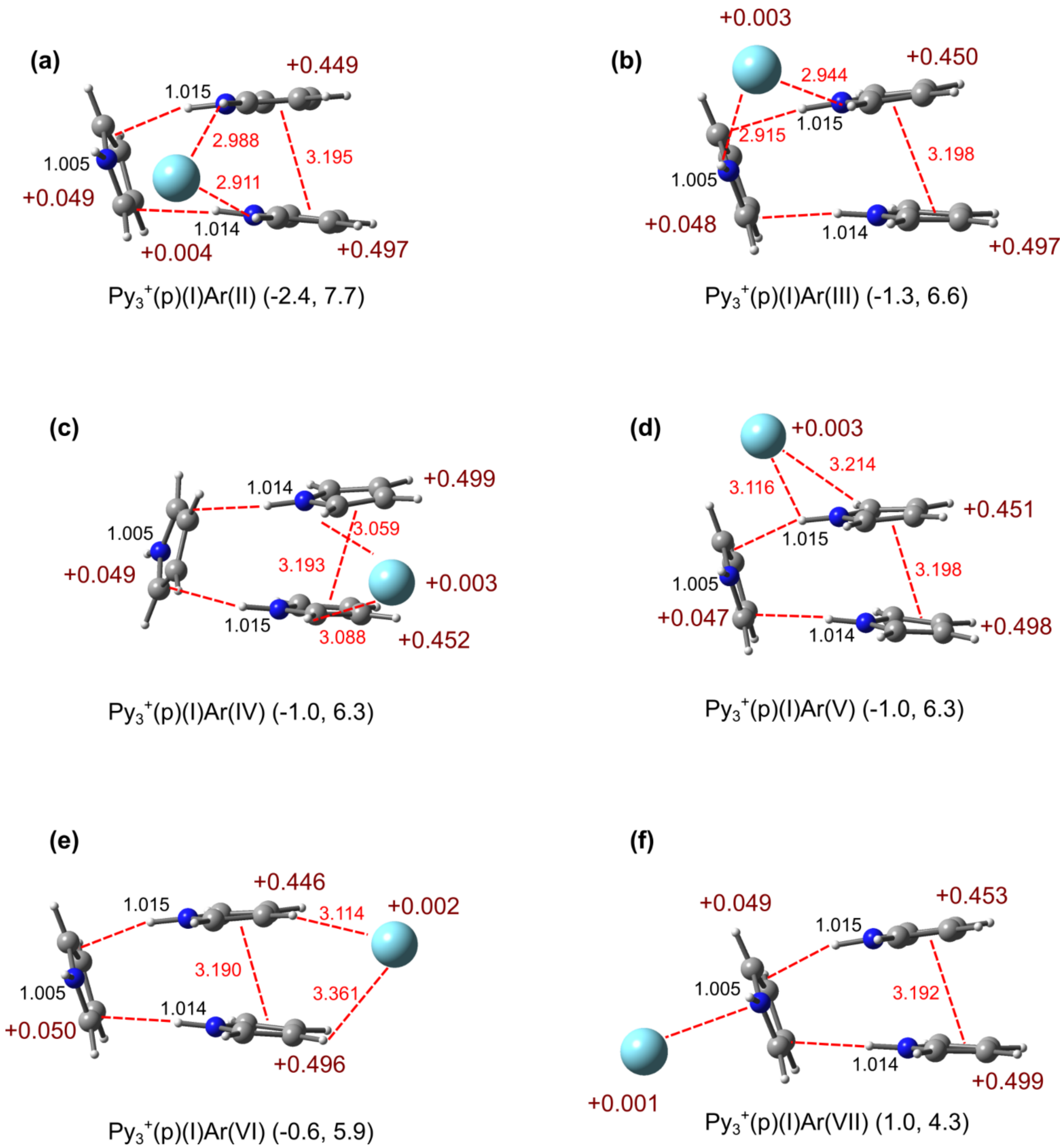

Figure S7. Structures of Py_3^+(p)(I)Ar(I-VII) obtained at the B3LYP-D3/aug-cc-pVTZ level. Selected intra- and intermolecular bond lengths (in Å) are given in black and red, respectively. Values in dark red indicate NBO charges (in units of *e*). Energies in parentheses are the relative energy and the dissociation energy of the most weakly bonded ligand (E_0 and D_0 in kJ mol^{-1}).

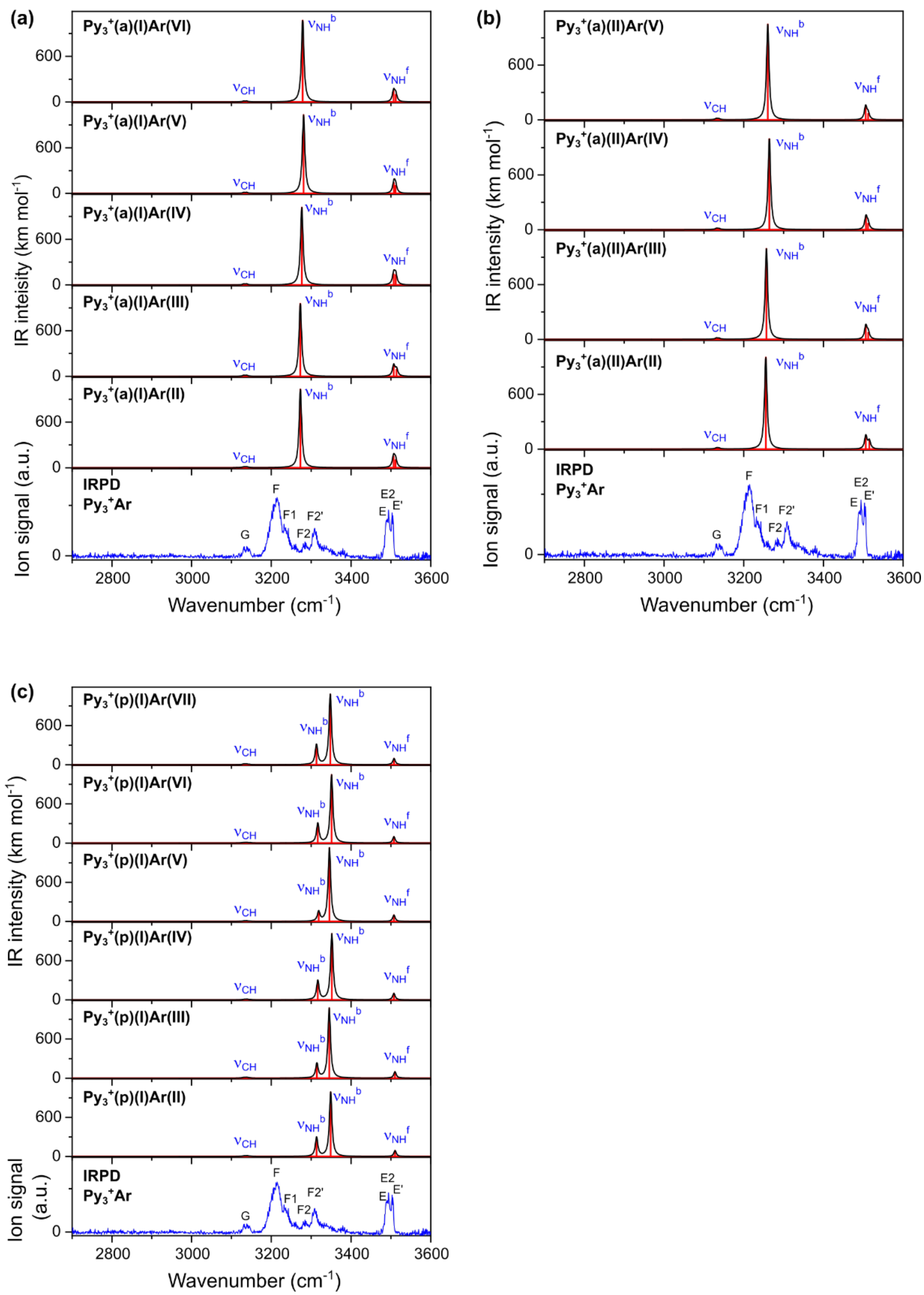

Figure S8. IRPD spectrum of Py_3^+Ar recorded in the CH and NH stretch range compared to the linear IR absorption spectra calculated for (a) Py_3^+(a)(I)Ar(II-VI), (b) Py_3^+(a)(II)Ar(I-V), and (c) Py_3^+(p)Ar(I-VII), at the B3LYP-D3/aug-cc-pVTZ level (Tables S1 and S4).

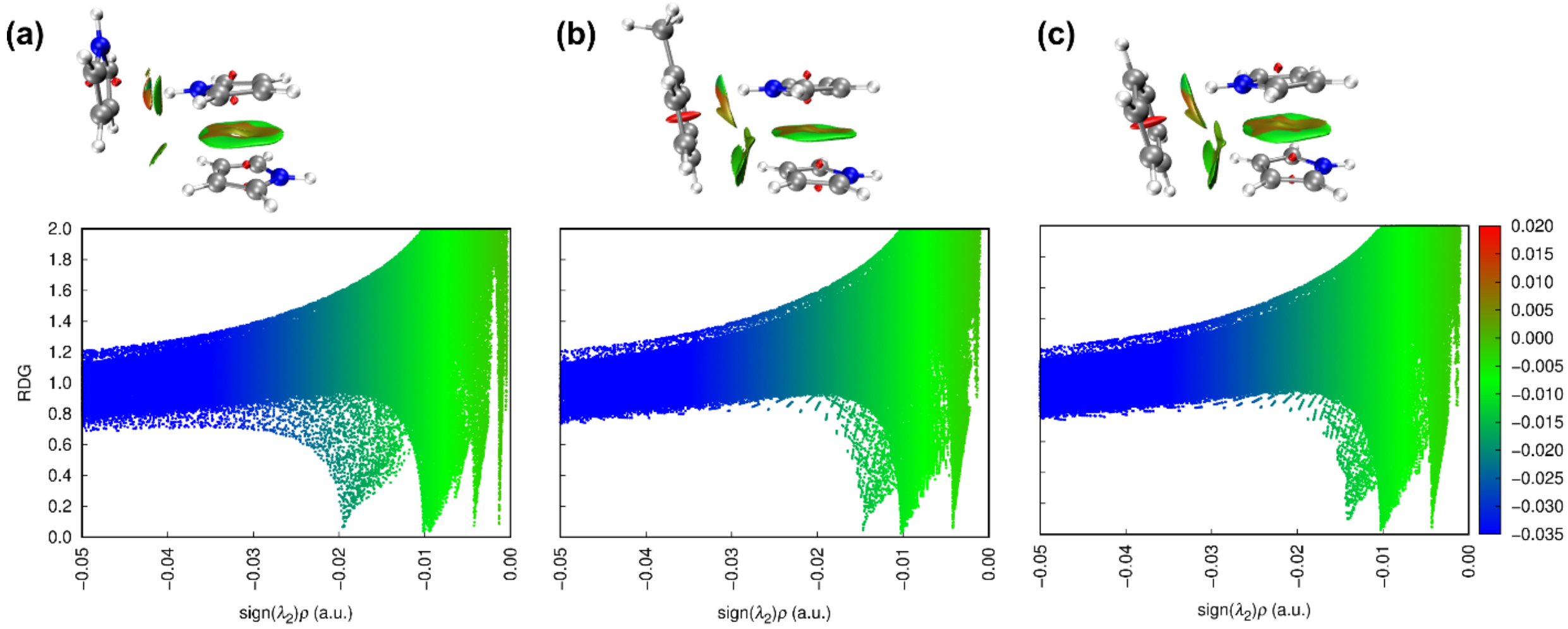

Figure S9. Noncovalent (NCI) interactions in (a) Py_3^+(a)(I), (b) Py_2^+(a)Tol(I), and (c) Py_2^+(a)Bz(I) visualized via the reduced electron density gradient (RDG), $s(\rho) \sim |\nabla(\rho)|/\rho^{4/3}$ as a function of the oriented electron density ρ^*=sign(λ_2)ρ. The plotted NCI surfaces cover the range -0.05 < ρ^* < 0.0 a.u., with an isosurface value of 0.3 a.u.. The NCI color code uses blue for attractive interactions (negative λ_2), green for weak van der Waals contacts ($\lambda_2 \leq 0$), and red for repulsive interactions (positive λ_2).

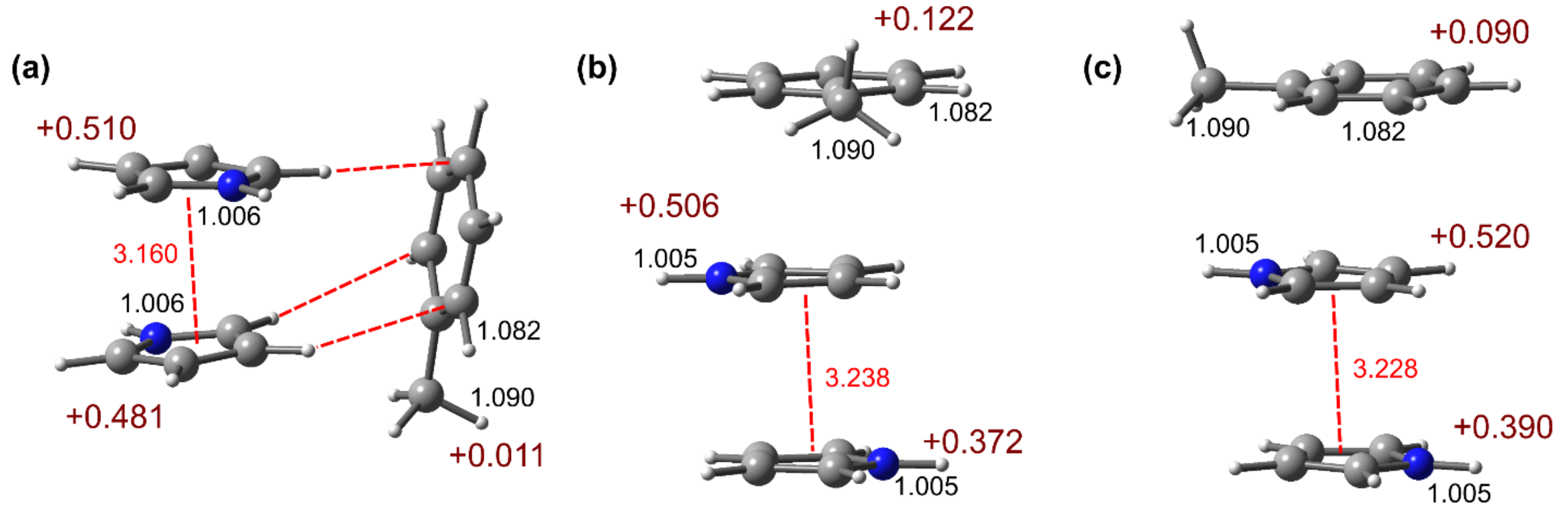

Py_2^+(a)Tol(II) (13.8, 38.5, 13.7) Py_2^+(a)Tol(III) (18.9, 33.4, 18.4) Py_2^+(a)Tol(IV) (19.8, 32.6, 20.3)

Figure S10. Structures of Py_2^+(a)Tol(II-IV) obtained at the B3LYP-D3/aug-cc-pVTZ level. Selected intra- and intermolecular bond lengths (in Å) are given in black and red, respectively. Values in dark red indicate NBO charges (in units of *e*). Energies in parentheses are the relative energy (E_0), the dissociation energy of the most weakly bonded ligand, and Gibbs free energy at 298 K (E_0, D_0, and G_0 in kJ mol^{-1}).

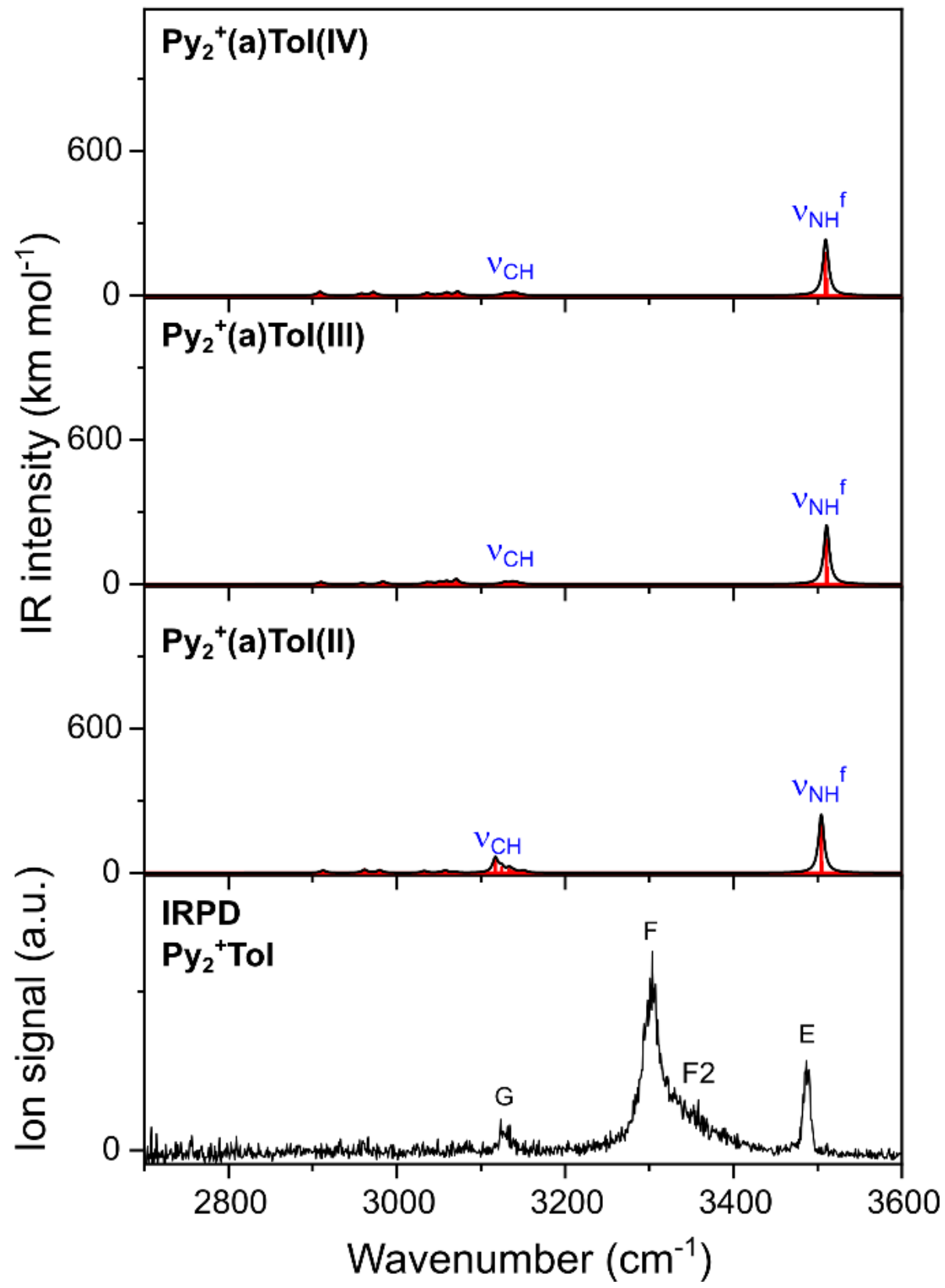

Figure S11. IRPD spectrum of Py_2^+Tol recorded in the CH and NH stretch range compared to the linear IR absorption spectra calculated for Py_2^+(a)Tol(II-IV) at the B3LYP-D3/aug-cc-pVTZ level (Tables S1 and S5).

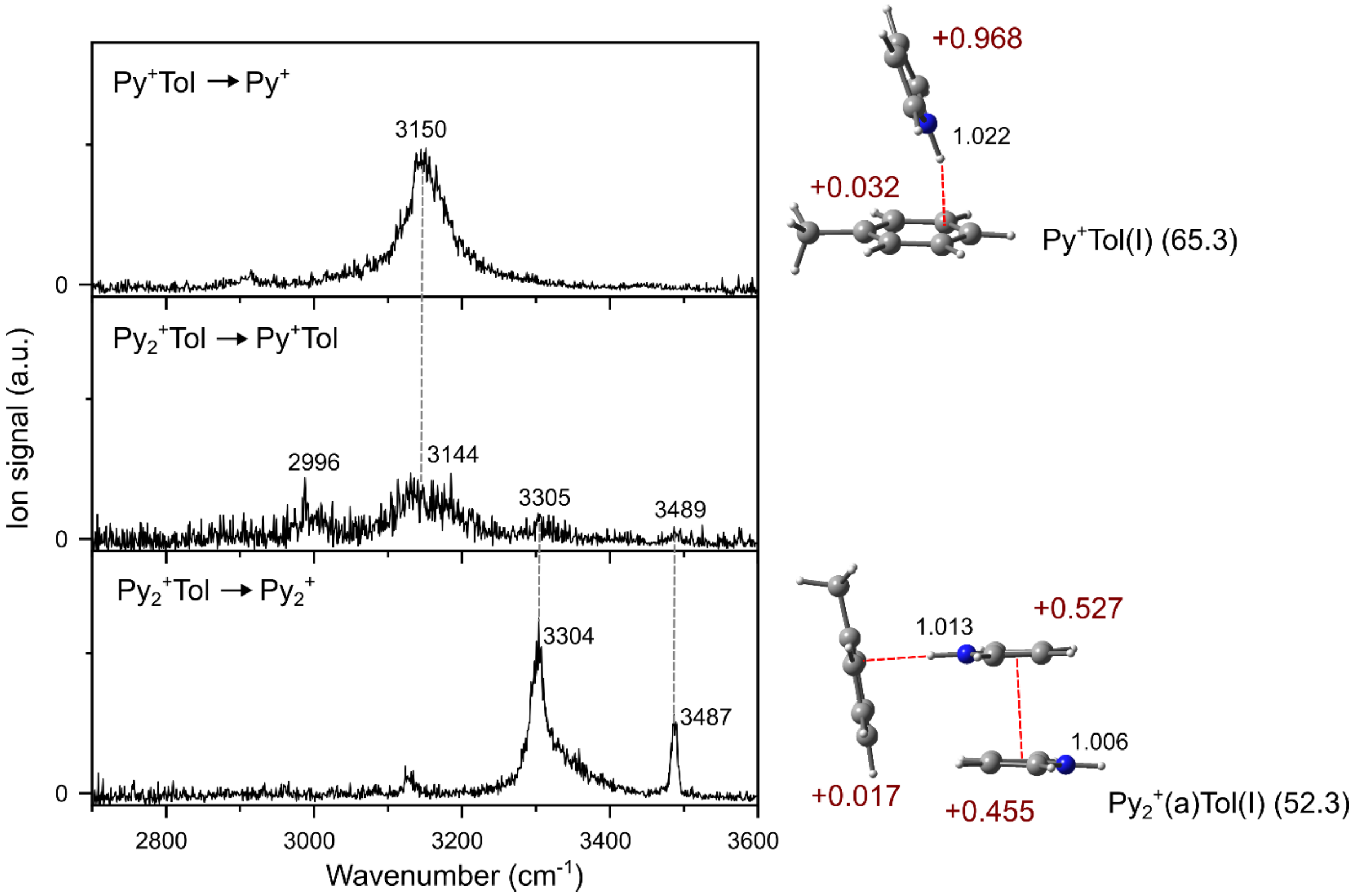

Figure S12. IRPD spectra of Py_2^+Tol recorded in the Py^+Tol fragment channel (middle), compared to IRPD spectra of Py_2^+Tol (bottom) and Py^+Tol (top)[2] measured in the Py_2^+ and Py^+ channels, respectively. Structures of the main contributors to the corresponding spectrum are shown on the right side. $Py^+Tol(I)$ is computed at the CAM-B3LYP-D3/aug-cc-pVTZ,[2] and $Py_2^+(a)Tol(I)$ is obtained at the B3LYP-D3/aug-cc-pVTZ level. Selected intramolecular bond lengths (in Å) are given in black. Values in dark red indicate NBO charges (in units of *e*). Energy in parentheses is the dissociation energy of the most weakly bonded ligand (in kJ mol^{-1}).

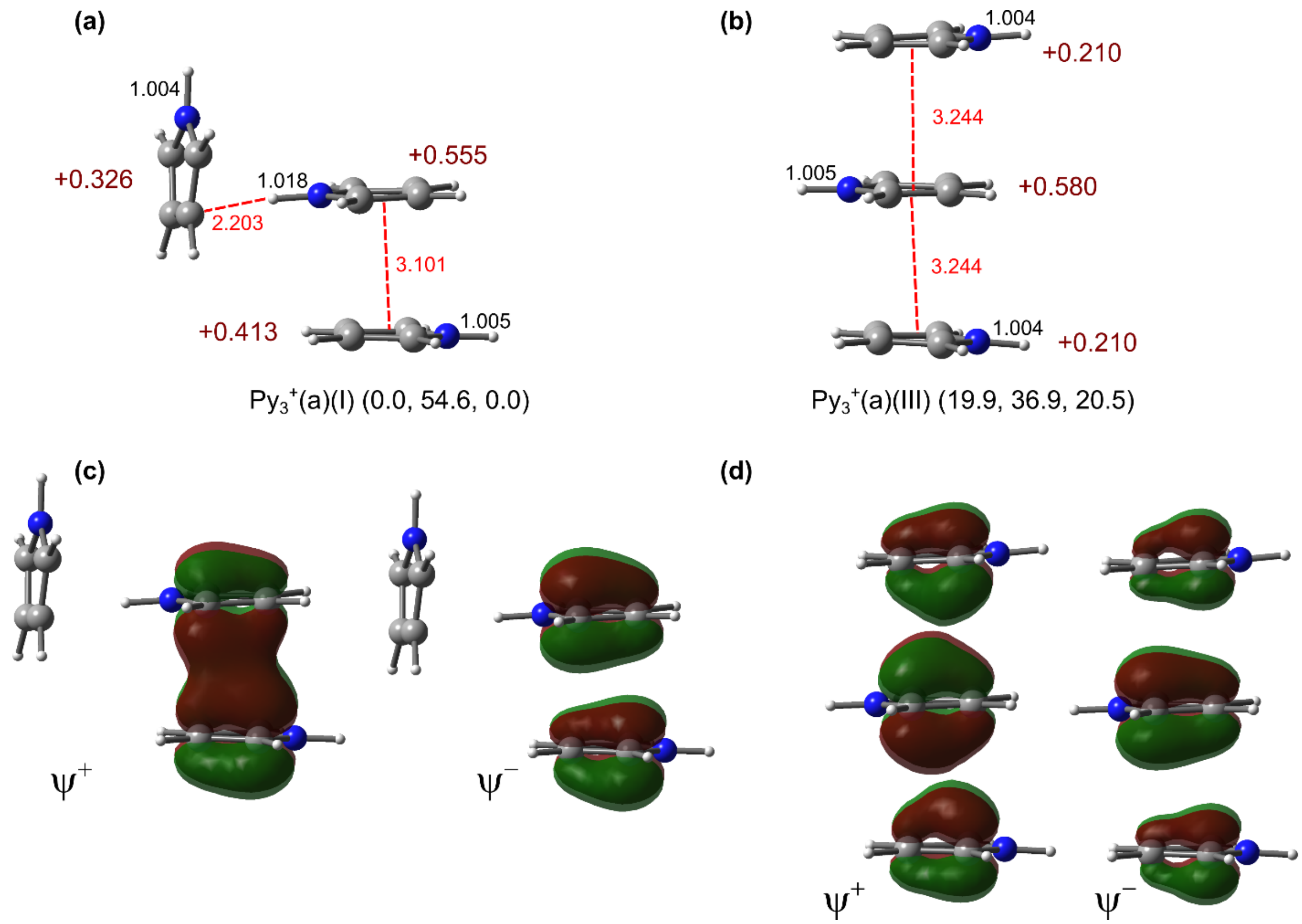

Figure S13. Structures and molecular orbitals of SOMO-1 and SOMO of Py_3^+(a)(I) and Py_3^+(a)(III) obtained at the CAM-B3LYP-D3/aug-cc-pVTZ level. Selected intra- and intermolecular bond lengths (in Å) are given in black and red, respectively. Values in dark red indicate NBO charges (in units of *e*). Energies in parentheses are the relative energy (E_0), the dissociation energy of the most weakly bonded ligand, and Gibbs free energy at 298 K (E_0, D_0, and G_0 in kJ mol^{-1}).

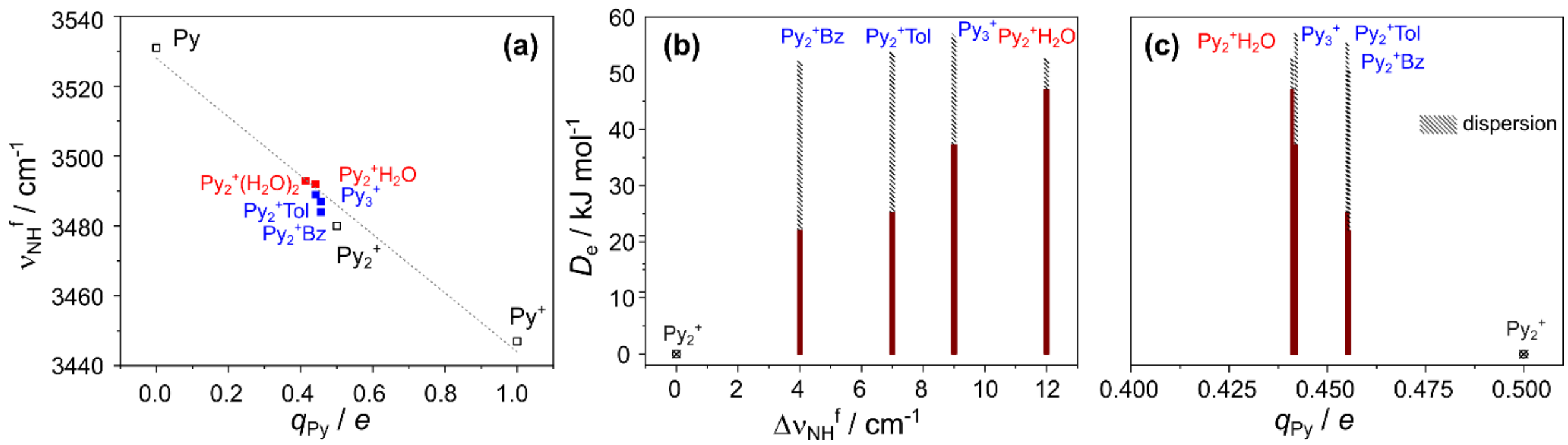

Figure S14. (a) Observed ν_{NH}^{f} values for Py, Py^+, and Py_2^+ (open symbols) and for $Py_2^+(H_2O)_{1,2}$, Py_3^+, Py_2^+Tol, and Py_2^+Bz (filled symbols) plotted as a function of the NBO charge on the free Py unit (q_{Py}) computed at the B3LYP-D3/aug-cc-pVTZ. The dashed line represents a linear fit based on ν_{NH}^{f} for Py, Py^+, and Py_2^+. Correlation between the computed dissociation energies (D_e) of the corresponding H-bonds uncorrected for zero-point vibrational energy, including the visualization of dispersion contributions, and (b) the observed $\Delta\nu_{NH}^{f}$ and (c) the computed NBO charges on the free Py unit for Py_2^+Bz, Py_2^+Tol, Py_3^+, and $Py_2^+H_2O$ trimers.

Table S1. Positions (in cm^{-1}) and suggested vibrational assignments of the CH and NH stretch transitions observed in the IRPD Spectra of $Py_3^+(Ar)$, Py_2^+Tol, and Py_2^+Bz compared to frequencies of the most stable isomers calculated at the B3LYP-D3/aug-cc-pVTZ Level.

	**exp**[a]	**isomer**	**calc**[b]	**mode**
Py_3^+	G 3130 (36)	Py_3^+(a)(I)	3108 (1), 3118 (0.2), 3120 (0.4), 3122 (0.6), 3130 (4), 3131 (8), 3134 (1), 3138 (0.4), 3138 (11), 3139 (0.3), 3145 (0.2), 3147 (1)	ν_{CH}
	F 3214 (54)	Py_3^+(a)(I)<br>Py_3^+(a)(II)	3276 (1026)<br>3262 (1002)	ν_{NH}^{b}
	F2 3328 (nr)	Py_3^+(p)(I)	3315 (270), 3349 (1050)	ν_{NH}^{b}
	E 3496 (19)	Py_3^+(a)(I)<br>Py_3^+(a)(II)<br>Py_3^+(a)(III)<br>Py_3^+(p)(I)	3506 (142), 3512 (100),<br>3506 (144), 3512 (70),<br>3496 (54), 3512 (150), 3515 (103)<br>3507 (98)	ν_{NH}^{f}
Py_3^+Ar	G 3137 (20)	Py_3^+(a)(II)Ar(I)	3108 (1), 3118 (1), 3123 (1), 3124 (1), 3131 (3), 3132 (4), 3132 (6), 3137 (0.1), 3137 (3), 3139 (7), 3145 (1), 3147 (1)	ν_{CH}
	F 3214 (27)	Py_3^+(a)(II)Ar(I)	3264 (1023)	ν_{NH}^{b}
	F1 3234 (15)	Py_3^+(a)(I)Ar(I)	3282 (1048)	ν_{NH}^{b}
	F2′ 3286 (8)	Py_3^+(p)(I)Ar(I)	3315 (330)	ν_{NH}^{b}
	F2 3309 (19)	Py_3^+(p)(I)Ar(I)	3351 (947)	ν_{NH}^{b}
	E 3489 (9)	Py_3^+(a)(I)Ar(I)<br>Py_3^+(a)(I)Ar(II)	3505 (176)<br>3504 (171)	ν_{NH}^{f}
	E2 3494 (3)	Py_3^+(p)(I)Ar(I)	3508 (100)	ν_{NH}^{f}
	E′ 3504 (7)	Py_3^+(a)(I)Ar(I)<br>Py_3^+(a)(I)Ar(II)	3512 (100)<br>3512 (69)	ν_{NH}^{f}
Py_2^+Tol	G 3128 (15)	Py_2^+(a)Tol(I)	2915 (8), 2967 (7), 2989 (9), 3038 (2), 3041 (1), 3051 (2), 3057 (9), 3068 (3), 3121 (0.4), 3127 (3), 3131 (5), 3133 (8), 3138 (4), 3139 (6), 3145 (0.7), 3148 (2)	ν_{CH}
	F 3304 (29)	Py_2^+(a)Tol(I)	3379 (711)	ν_{NH}^{b}
	E 3487 (9)	Py_2^+(a)Tol(I)	3505 (147)	ν_{NH}^{f}
Py_2^+Bz	G 3131 (10)	Py_2^+(a)Bz(I)	3042 (0.1), 3050 (0.4), 3052 (0.2), 3065 (6), 3074 (0.1), 3121 (0.4), 3127 (3), 3131 (5), 3133 (8), 3138 (4), 3140 (5), 3145 (1), 3148 (2)	ν_{CH}
	F 3321 (23)	Py_2^+(a)Bz(I)	3386 (651)	ν_{NH}^{b}
	E 3484 (10)	Py_2^+(a)Bz(I)	3505 (146)	ν_{NH}^{f}

[a] Peak positions and widths (in parentheses) are obtained from deconvolution. nr=not resolved, broad. [b] IR intensities in parentheses are given in km mol^{-1}

Table S2. Calculated NH and CH stretch frequencies of Py_3^+(a)(III-IV) and Py_3^+(p)(II) obtained at the B3LYP-D3/aug-cc-pVTZ level.

isomer	calc (IR intensity in km mol^{-1})	mode
Py_3^+(a)(III)	3134 (0.5), 3139 (0.7), 3121 (0.2), 3124 (3), 3128 (10), 3132 (2), 3137 (3), 3138 (3), 3143 (1), 3144 (2), 3144 (5), 3153 (2)	ν_{CH}
	3496 (54), 3512 (150), 3515 (103)	ν_{NH}^{f}
Py_3^+(a)(IV)	3118 (0.8), 3119 (0.8), 3123 (0.1), 3126 (8), 3128 (9), 3135 (1), 3138 (3), 3143 (3), 3144 (0.2), 3146 (6), 3152 (0.3)	ν_{CH}
	3509 (141), 3512 (144), 3520 (72)	ν_{NH}^{f}
Py_3^+(p)(I)	3104 (0.5), 3116 (1), 3122 (0), 3123 (0.5), 3128 (3), 3130 (3), 3132 (4), 3135 (1), 3136 (3), 3145 (0.7), 3146 (4)	ν_{CH}
	3315 (270), 3349 (1050)	ν_{NH}^{b}
	3507 (98)	ν_{NH}^{f}
Py_3^+(p)(II)	3103 (0.6), 3114 (1), 3123 (0), 3124 (0.6), 3131 (2), 3132 (6), 3135 (3), 3137 (3), 3139 (0.2), 3139 (8), 3146 (3)	ν_{CH}
	3373 (389), 3430 (570)	ν_{NH}^{b}
	3496 (93)	ν_{NH}^{f}

Table S3. The computed relative and dissociation energies for Py_3^+, Py_2^+Tol, and Py_2^+Bz isomers at various computational levels (in kJ mol^{-1}). Single-point energies at the DLPNO-CCSD(T) are calculated for the geometries optimized using the B3LYP-D3 and CAM-B3LYP-D3 functionals.

	isomers	B3LYP-D3[a]		CAM-B3LYP-D3[a]		CBS-QB3		DLPNO-CCSD(T)[a]			
		E_0	D_0	E_0	D_0	E_0	D_0	E_e		D_e	
								B3LYP	CAM-B3LYP	B3LYP	CAM-B3LYP
Py_3^+	Py_3^+(a)(I)	0.0	54.0	0.0	54.6	0.0	42.9	0.0	0.0	57.4	60.3
	Py_3^+(a)(II)	2.1	51.9	2.4	52.2	3.3	39.6	1.9	2.0	55.5	58.4
	Py_3^+(a)(III)	5.0	49.0	19.9	34.7	--	--	23.2	27.8	34.2	32.5
	Py_3^+(p)(I)	-1.9	63.3	0.5	62.7	1.4	48.4	1.4	0.4	71.3	74.1
Py_2^+Tol	Py_2^+(a)Tol(I)	0.0	52.3	0.0	51.9	0.0	31.4	0.0	0.0	58.9	59.8
	Py_2^+(p)Tol(I)	-1.3	61.1	0.2	60.2	0.1	38.2	2.2	1.7	72.0	72.3
Py_2^+Bz	Py_2^+(a)Bz(I)	0.0	47.3	0.0	47.1	0.0	28.3	0.0	0.0	53.6	54.9
	Py_2^+(p)Bz(I)	-0.2	55.0	1.3	54.4	0.6	34.6	0.0	2.5	69.0	66.5

[a]The basis set is aug-cc-pVTZ.

Table S4. Calculated NH and CH stretch frequencies of Py_3^+(a)(I)Ar(II-VI), Py_3^+(a)(II)Ar(II-V), Py_3^+(p)(I)Ar(II-VII) obtained at the B3LYP-D3/aug-cc-pVTZ level.

isomer	calc (IR intensity in km mol^{-1})	mode
Py_3^+(a)(I)Ar(II)	3113 (1), 3120 (0.4), 3122 (0.3), 3125 (2), 3130 (4), 3133 (7), 3135 (1), 3138 (4) 3139 (7), 3140 (0.3), 3145 (1), 3147 (0.3)	ν_{CH}
	3273 (1030)	$\nu_{NH}{}^{b}$
	3507 (145), 3512 (102)	$\nu_{NH}{}^{f}$
Py_3^+(a)(I)Ar(III)	3109 (1), 3119 (0.3), 3120 (0.4), 3122 (0.5), 3130 (3), 3131 (9), 3134 (1), 3138 (0.8), 3138 (11), 3139 (0.2), 3145 (0.2), 3146 (1)	ν_{CH}
	3273 (954)	$\nu_{NH}{}^{b}$
	3507 (143), 3514 (92)	$\nu_{NH}{}^{f}$
Py_3^+(a)(I)Ar(IV)	3108 (1), 3118 (0.1), 3120 (0.4), 3122 (0.6), 3130 (4), 3131 (8), 3135 (1), 3138 (0.4), 3138 (11), 3145 (0.1), 3147 (1)	ν_{CH}
	3276 (1017)	$\nu_{NH}{}^{b}$
	3507 (143), 3512 (132)	$\nu_{NH}{}^{f}$
Py_3^+(a)(I)Ar(IV)	3108 (1), 3118 (0.1), 3120 (0.4), 3122 (0.6), 3130 (4), 3131 (7), 3134 (1), 3138 (2), 3139 (9), 3139 (0.4), 3145 (0.2), 3147 (1)	ν_{CH}
	3281 (1031)	$\nu_{NH}{}^{b}$
	3507 (138), 3512 (100)	$\nu_{NH}{}^{f}$
Py_3^+(a)(I)Ar(VI)	3108 (1), 3118 (0.1), 3120 (0.4), 3122 (0.6), 3130 (4), 3131 (8), 3135 (2), 3138 (0.5), 3138 (11), 3139 (0.2), 3145 (0.2), 3147 (1)	ν_{CH}
	3279 (1071)	$\nu_{NH}{}^{b}$
	3507 (143), 3512 (97)	$\nu_{NH}{}^{f}$
Py_3^+(a)(II)Ar(II)	3109 (0.6), 3119 (0.5), 3121 (0.4), 3126 (3), 3130 (5), 3133 (3), 3137 (5), 3138 (0.1), 3139 (5), 3144 (1)	ν_{CH}
	3255 (1004)	$\nu_{NH}{}^{b}$
	3506 (144), 3515 (87)	$\nu_{NH}{}^{f}$
Py_3^+(a)(II)Ar(III)	3110 (0.7), 3120 (0.4), 3121 (0.4), 3124 (2), 3132 (9), 3138 (2), 3138 (0.5), 3138 (8), 3145 (1), 3146 (0.6)	ν_{CH}
	3257 (992)	$\nu_{NH}{}^{b}$
	3506 (144), 3512 (74)	$\nu_{NH}{}^{f}$
Py_3^+(a)(II)Ar(IV)	3108 (0.6), 3118 (0.5), 3121 (0.5), 3125 (2), 3131 (5), 3132 (1), 3133 (8), 3137 (5), 3137 (0.1), 3139 (6), 3144 (1), 3147 (1)	ν_{CH}
	3264 (993)	$\nu_{NH}{}^{b}$

	3507 (139), 3512 (68)	ν_{NH}^{f}
Py_3^+(a)(II)Ar(V)	3108 (0.6), 3118 (0.6), 3118 (0.6), 3121 (0.5), 3124 (2), 3130 (5), 3132 (8), 3133 (2), 3137 (4), 3137 (0.1), 3139 (6), 3144 (1), 3147 (0.7)	ν_{CH}
	3260 (1045)	ν_{NH}^{b}
	3506 (145), 3512 (68)	ν_{NH}^{f}
Py_3^+(p)(I)Ar(II)	3104 (0.5), 3117 (1), 3123 (0.1), 3124 (0.7), 3129 (3), 3131 (2), 3133 (6), 3135 (1), 3138 (4), 3140 (9), 3146 (2), 3148 (2)	ν_{CH}
	3314 (290), 3349 (985)	ν_{NH}^{b}
	3511 (91)	ν_{NH}^{f}
Py_3^+(p)(I)Ar(III)	3104 (0.5), 3117 (0.8), 3122 (0.1), 3124 (0.6), 3128 (2),3131 (2), 3132 (6), 3136 (1), 3138 (2), 3139 (10), 3145 (0.5), 3146 (2)	ν_{CH}
	3314 (220), 3345 (1071)	ν_{NH}^{b}
	3510 (98)	ν_{NH}^{f}
Py_3^+(p)(I)Ar(IV)	3104 (0.5), 3116 (1), 3123 (0.05), 3124 (0.8), 3128 (3), 3132 (1), 3133 (4), 3135 (1), 3137 (3), 3140 (7), 3146 (1), 3148 (3)	ν_{CH}
	3317 (294), 3352 (1012)	ν_{NH}^{b}
	3508 (99)	ν_{NH}^{f}
Py_3^+(p)(I)Ar(V)	3104 (0.6), 3119 (1), 3122 (0.02), 3124 (2), 3131 (2), 3132 (5), 3136 (0.8), 3137 (2), 3138 (8), 3145 (0.0), 3146 (5)	ν_{CH}
	3319 (146), 3346 (1126)	ν_{NH}^{b}
	3508 (97)	ν_{NH}^{f}
Py_3^+(p)(I)Ar(VI)	3104 (0.5), 3116 (1), 3124 (0.1), 3125 (1), 3128 (3), 3131 (2), 3133 (3), 3135 (1), 3137 (1), 3139 (7), 3146 (0.2), 3147 (5)	ν_{CH}
	3317 (301), 3351 (1046)	ν_{NH}^{b}
	3508 (98)	ν_{NH}^{f}
Py_3^+(p)(I)Ar(VII)	3104 (0.5), 3122 (0.0), 3123 (0.6), 3129 (3), 3130 (3), 3132 (4), 3135 (2), 3136 (2), 3138 (7), 3145 (0.7), 3147 (4)	ν_{CH}
	3313 (305), 3348 (1077)	ν_{NH}^{b}
	3508 (95)	ν_{NH}^{f}

Table S5. Calculated NH and CH stretch frequencies of Py_2^+(a)Tol(II)-(IV) obtained at the B3LYP-D3/aug-cc-pVTZ level.

isomer	calc (IR intensity in km mol^{-1})	mode
Py_2^+(a)Tol(II)	2912 (10), 2962 (13), 2980 (11), 3032 (4), 3033 (4), 3049 (0.8), 3057 (10), 3069 (3), 3116 (60), 3123 (1), 3124 (21), 3133 (13), 3135 (7), 3134 (5), 3142 (0.2), 3151 (10)	ν_{CH}
	3504 (202)	ν_{NH}^f
Py_2^+(a)Tol(III)	2910 (9), 2959 (4), 2984 (11), 3035 (6), 3041 (4), 3051 (11), 3071 (20), 3119 (0.5), 3128 (8), 3129 (0.6), 3136 (5), 3138 (7), 3142 (7), 3144 (0.1), 3151 (1)	ν_{CH}
	3510 (182)	ν_{NH}^f
Py_2^+(a)Tol(IV)	2909 (15), 2958 (7), 2972 (14), 3036 (7), 3037 (3), 3052 (3), 3059 (11), 3072 (16), 3119 (0.5), 3128 (0.5), 3129 (8), 3136 (5), 3138 (4), 3142 (8), 3144 (0.05), 3150 (1)	ν_{CH}
	3510 (173)	ν_{NH}^f

Table S6. Computed D_e (in kJ mol^{-1}) of Py_2^+X (X=Bz, Tol, Py, and H_2O) without and with the empirical dispersion correction (D3) at the CAM-B3LYP and B3LYP levels with aug-cc-pVTZ as a basis set.

	D_e **(CAM-B3LYP)**	D_e **(CAM-B3LYP-D3)**	D_e **(B3LYP)**	D_e **(B3LYP-D3)**
Py_2^+Bz	29.4	49.9	22.0	50.4
Py_2^+Tol	32.5	54.7	25.2	55.4
Py_3^+	44.7	57.4	37.3	57.0
$Py_2^+H_2O$	51.5	55.5	47.1	52.6

References

[1]. C. Wei, X. Chen, Y. Wang, et al., “Single-Photon Ionization Induced C-C or C-N Bond Formation in Pyrrole Clusters,” *Physical Chemistry Chemical Physics* 25 (2023): 11368–11374.
[2]. D. Arildii, and O. Dopfer, “Competition between π-π and NH···π Interactions in $Pyrrole^+$-Benzene and $Pyrrole^+$-Toluene Radical Cations Revealed by IR Spectroscopy,” *The Journal of Physical Chemistry A* 129 (2025): 10161–10171.

Cartesian coordinates

Center number	Atomic number	Coordinates (Angstroms)		
		X	Y	Z
		**at B3LYP-D3/aug-cc-pVTZ**		

Ar

1	18	0	0	0

Sum of electronic and zero-point Energies= -527.560002
Sum of electronic and thermal Energies= -527.558586
Sum of electronic and thermal Enthalpies= -527.557642
Sum of electronic and thermal Free Energies= -527.575214

Py

1	6	0	-1.12221	0.33055
2	6	0	-0.7108	-0.98012
3	6	0	0.7108	-0.98012
4	6	0	1.12221	0.33055
5	7	0	0	1.11899
6	1	0	0	2.12208
7	1	0	-2.10766	0.76139
8	1	0	-1.35633	-1.84151
9	1	0	1.35633	-1.84151
10	1	0	2.10766	0.76139

Sum of electronic and zero-point Energies= -210.173493
Sum of electronic and thermal Energies= -210.169551
Sum of electronic and thermal Enthalpies= -210.168607
Sum of electronic and thermal Free Energies= -210.199212

Py^+

1	6	0	-1.10622	0.35168
2	6	0	-0.6841	-1.01351
3	6	0	0.6841	-1.01351
4	6	0	1.10622	0.35168
5	7	0	0	1.13966
6	1	0	0	2.14989
7	1	0	-2.10118	0.76883
8	1	0	-1.34782	-1.86156
9	1	0	1.34782	-1.86156
10	1	0	2.10118	0.76883

Sum of electronic and zero-point Energies= -209.876187
Sum of electronic and thermal Energies= -209.872202
Sum of electronic and thermal Enthalpies= -209.871258
Sum of electronic and thermal Free Energies= -209.902599

Py_2^+(a)

1	6	1.56263	0.60234	1.11273
2	6	1.56263	-0.73394	0.69654
3	6	1.56263	-0.73394	-0.69654
4	6	1.56263	0.60234	-1.11273
5	7	1.58039	1.38897	0
6	1	1.59458	2.39502	0
7	1	1.59127	1.02447	2.10213
8	1	1.59163	-1.58615	1.35251
9	1	1.59163	-1.58615	-1.35251
10	1	1.59127	1.02447	-2.10213
11	6	-1.56263	-0.60234	-1.11273
12	6	-1.56263	0.73394	-0.69654
13	6	-1.56263	0.73394	0.69654
14	6	-1.56263	-0.60234	1.11273
15	7	-1.58039	-1.38897	0
16	1	-1.59458	-2.39502	0
17	1	-1.59127	-1.02447	-2.10213
18	1	-1.59163	1.58615	-1.35251
19	1	-1.59163	1.58615	1.35251
20	1	-1.59127	-1.02447	2.10213

Sum of electronic and zero-point Energies= -420.090494
Sum of electronic and thermal Energies= -420.081137
Sum of electronic and thermal Enthalpies= -420.080193
Sum of electronic and thermal Free Energies= -420.126263

Py_2^+(p)

1	6	1.44906	1.11081	-0.53758
2	6	2.04218	0.69853	0.66615
3	6	2.04564	-0.68926	0.67216
4	6	1.45455	-1.11478	-0.52775
5	7	1.12222	-0.00583	-1.24542
6	1	0.71694	-0.0109	-2.16543
7	1	1.3012	2.09776	-0.93954
8	1	2.42025	1.35744	1.42817
9	1	2.42675	-1.33962	1.43998
10	1	1.31081	-2.10587	-0.92094
11	6	-1.74304	1.11374	-0.08667
12	6	-1.20324	0.70151	1.13277
13	6	-1.20289	-0.69428	1.13781
14	6	-1.74241	-1.11547	-0.07851
15	7	-2.06201	-0.00352	-0.80696
16	1	-2.54561	-0.00684	-1.68938
17	1	-1.95076	2.10169	-0.4602
18	1	-0.8757	1.35735	1.91951
19	1	-0.8749	-1.34422	1.92924
20	1	-1.94945	-2.10617	-0.44505

Sum of electronic and zero-point Energies= -420.087601
Sum of electronic and thermal Energies= -420.078127
Sum of electronic and thermal Enthalpies= -420.077182
Sum of electronic and thermal Free Energies= -420.124899

Py_3^+(a)(I)

1	6	0.15738	1.04385	1.02274
2	6	1.31231	1.83825	0.91774
3	6	1.52968	2.06669	-0.43503
4	6	0.50166	1.41112	-1.13452
5	7	-0.32179	0.8245	-0.22869
6	1	-1.12392	0.23799	-0.44981
7	1	-0.35617	0.6612	1.8866
8	1	1.88921	2.2083	1.74713
9	1	2.31142	2.65289	-0.88547
10	1	0.30156	1.36241	-2.19073
11	6	3.11436	-0.65605	-1.02764
12	6	1.9248	-1.38145	-0.93897
13	6	1.67227	-1.60199	0.4162
14	6	2.71136	-1.00722	1.13471
15	7	3.58041	-0.4578	0.23838
16	1	4.42604	0.03207	0.47538
17	1	3.66026	-0.31282	-1.88886
18	1	1.33951	-1.71876	-1.77612
19	1	0.84799	-2.14209	0.84726
20	1	2.90043	-0.97699	2.19337
21	6	-3.39911	-0.09358	1.12057
22	6	-2.72566	-1.23114	0.74159
23	6	-2.73093	-1.27249	-0.68292
24	6	-3.41247	-0.15781	-1.12308
25	7	-3.8061	0.54939	-0.01887
26	1	-4.3508	1.39375	-0.04016
27	1	-3.6392	0.29261	2.09533
28	1	-2.30991	-1.96732	1.408
29	1	-2.32922	-2.05038	-1.30969
30	1	-3.66882	0.16533	-2.11646

Sum of electronic and zero-point Energies= -630.284574
Sum of electronic and thermal Energies= -630.269328
Sum of electronic and thermal Enthalpies= -630.268384
Sum of electronic and thermal Free Energies= -630.33358

Py_3^+(a)(II)

1	6	0.52401	1.52419	1.07467
2	6	1.8721	1.81848	0.79943
3	6	2.00169	1.88465	-0.58107
4	6	0.73162	1.63078	-1.12762
5	7	-0.14102	1.43809	-0.10491
6	1	-1.12696	1.20075	-0.20274
7	1	0.0117	1.4078	2.01353
8	1	2.63444	1.98559	1.53998
9	1	2.88701	2.11333	-1.14802

10	1	0.40695	1.61217	-2.1534
11	6	2.4307	-1.24259	-1.0941
12	6	1.08138	-1.43418	-0.79661
13	6	0.9631	-1.47382	0.59465
14	6	2.24324	-1.30627	1.12514
15	7	3.11589	-1.18438	0.0843
16	1	4.10995	-1.05942	0.17186
17	1	2.93552	-1.17994	-2.04214
18	1	0.2937	-1.53877	-1.52115
19	1	0.06348	-1.60954	1.1676
20	1	2.58094	-1.30067	2.14661
21	6	-2.96527	-0.41682	-1.16213
22	6	-3.24602	0.74683	-0.48031
23	6	-3.13808	0.4593	0.91216
24	6	-2.79557	-0.86669	1.03096
25	7	-2.68723	-1.38506	-0.23427
26	1	-2.52262	-2.3534	-0.44634
27	1	-2.97623	-0.63616	-2.21519
28	1	-3.54555	1.68031	-0.92613
29	1	-3.32643	1.13729	1.72697
30	1	-2.65681	-1.48306	1.90163

Sum of electronic and zero-point Energies= -630.283784
Sum of electronic and thermal Energies= -630.268602
Sum of electronic and thermal Enthalpies= -630.267658
Sum of electronic and thermal Free Energies= -630.332052

Py$_3^+$(a)(III)

1	6	3.34089	0.84462	-0.78692
2	6	2.83261	1.38072	0.39159
3	6	2.89195	0.37637	1.36754
4	6	3.43621	-0.75364	0.76706
5	7	3.70939	-0.44568	-0.53469
6	1	4.13075	-1.06876	-1.20136
7	1	3.49277	1.29418	-1.75221
8	1	2.48832	2.39223	0.5167
9	1	2.60239	0.45297	2.40083
10	1	3.67062	-1.72011	1.17701
11	6	-0.01791	-0.9162	0.75248
12	6	0.49302	-1.42772	-0.44371
13	6	0.43786	-0.40241	-1.38791
14	6	-0.11057	0.71899	-0.75144
15	7	-0.36598	0.38176	0.53876
16	1	-0.87394	0.95548	1.19073
17	1	-0.15559	-1.38409	1.71066
18	1	0.85496	-2.43061	-0.58642
19	1	0.74596	-0.44266	-2.41777
20	1	-0.31496	1.70478	-1.12672
21	6	-3.17281	0.43937	-1.07288
22	6	-3.49347	1.25081	0.00669
23	6	-3.59042	0.42114	1.14231
24	6	-3.3287	-0.8755	0.72917
25	7	-3.08253	-0.8431	-0.61505
26	1	-2.87217	-1.64488	-1.18288
27	1	-3.05205	0.67273	-2.11549
28	1	-3.6653	2.3125	-0.03828
29	1	-3.85427	0.71813	2.14279
30	1	-3.33567	-1.80204	1.27536

Sum of electronic and zero-point Energies= -630.282672
Sum of electronic and thermal Energies= -630.267401
Sum of electronic and thermal Enthalpies= -630.266457
Sum of electronic and thermal Free Energies= -630.330606

Py$_3^+$(a)(IV)

1	6	-3.39151	1.11543	0.0587
2	6	-2.95243	0.7033	-1.19387
3	6	-2.95218	-0.6996	-1.1964
4	6	-3.39105	-1.11636	0.05466
5	7	-3.66143	-0.00183	0.79554
6	1	-4.01389	-0.00359	1.73674
7	1	-3.55712	2.10229	0.45311
8	1	-2.69389	1.35723	-2.00817
9	1	-2.69337	-1.35048	-2.01304
10	1	-3.55601	-2.1047	0.44564
11	6	0.05864	-1.11254	-0.05419
12	6	-0.39527	-0.6958	1.20156
13	6	-0.3952	0.69659	1.20073
14	6	0.05895	1.11179	-0.05554
15	7	0.31882	-7.83E-4	-0.79415
16	1	0.69899	-0.00139	-1.7238
17	1	0.2034	-2.10087	-0.45092
18	1	-0.66959	-1.35046	2.00957
19	1	-0.66948	1.35232	2.00789
20	1	0.20373	2.09964	-0.45348
21	6	3.42125	-1.1196	-0.32521
22	6	3.0927	-0.70335	0.95225
23	6	3.09317	0.70613	0.95036
24	6	3.42212	1.11874	-0.32822
25	7	3.6071	-0.0015	-1.09656
26	1	3.95831	-0.0029	-2.03838
27	1	3.56369	-2.10698	-0.72817
28	1	2.8961	-1.3502	1.78876
29	1	2.89714	1.35536	1.78517
30	1	3.56551	2.10495	-0.73369

Sum of electronic and zero-point Energies= -630.280920
Sum of electronic and thermal Energies= -630.265552
Sum of electronic and thermal Enthalpies= -630.264608
Sum of electronic and thermal Free Energies= -630.328750

Py$_3^+$(p)(I)

1	6	-1.66168	-1.24619	1.1447
2	6	-2.97561	-1.30411	0.64807
3	6	-2.88954	-1.39354	-0.73425
4	6	-1.52618	-1.38903	-1.06657
5	7	-0.81013	-1.32594	0.08897
6	1	0.20089	-1.28621	0.14995
7	1	-1.30123	-1.21654	2.15774
8	1	-3.86807	-1.28474	1.24877
9	1	-3.70111	-1.45681	-1.43774
10	1	-1.04255	-1.47717	-2.02334
11	6	-0.77365	1.70323	1.08824
12	6	-2.03983	1.86937	0.51618
13	6	-1.87903	1.80914	-0.86636
14	6	-0.51687	1.61054	-1.11593
15	7	0.13312	1.56387	0.08046
16	1	1.12381	1.38584	0.21137
17	1	-0.4672	1.71501	2.11974
18	1	-2.95334	2.02666	1.06159
19	1	-2.64177	1.91019	-1.61792
20	1	0.0171	1.53626	-2.04716
21	6	3.34065	0.71218	-0.05765
22	6	2.92271	0.15285	1.12906
23	6	2.4701	-1.16603	0.84048
24	6	2.62625	-1.36643	-0.51367
25	7	3.1417	-0.21561	-1.04846
26	1	3.42727	-0.10372	-2.00591
27	1	3.79135	1.66665	-0.26692
28	1	2.98163	0.61843	2.09817
29	1	2.13913	-1.90364	1.55266
30	1	2.45724	-2.23829	-1.12159

Sum of electronic and zero-point Energies= -630.285280
Sum of electronic and thermal Energies= -630.270237
Sum of electronic and thermal Enthalpies= -630.269293
Sum of electronic and thermal Free Energies= -630.332609

Py$_3^+$(p)(II)

1	6	-1.65413	-1.28457	1.11551
2	6	-2.98953	-1.28201	0.6783
3	6	-2.96765	-1.27716	-0.70861
4	6	-1.61833	-1.27682	-1.10397
5	7	-0.85219	-1.30954	0.01776
6	1	0.15939	-1.28002	0.03327
7	1	-1.24654	-1.32379	2.1102

8	1	-3.85343	-1.28651	1.3196
9	1	-3.81082	-1.27756	-1.37699
10	1	-1.17978	-1.31234	-2.08548
11	6	-0.55772	1.65161	1.11159
12	6	-1.87397	1.87725	0.69997
13	6	-1.87767	1.87282	-0.69473
14	6	-0.5636	1.64375	-1.11212
15	7	0.21938	1.52692	-0.00185
16	1	1.20851	1.32582	-0.00407
17	1	-0.13269	1.60466	2.09893
18	1	-2.71213	2.03739	1.35461
19	1	-2.71928	2.02864	-1.34597
20	1	-0.14352	1.59145	-2.10126
21	6	3.00519	-0.13385	1.1286
22	6	2.45691	-1.3191	0.69948
23	6	2.46076	-1.30081	-0.72632
24	6	3.01143	-0.10535	-1.1219
25	7	3.32334	0.60501	0.0137
26	1	3.86081	1.45568	0.02616
27	1	3.22305	0.22664	2.11834
28	1	2.14246	-2.12915	1.33634
29	1	2.14583	-2.09287	-1.38498
30	1	3.23432	0.28073	-2.10082

Sum of electronic and zero-point Energies= -630.284297
Sum of electronic and thermal Energies= -630.269195
Sum of electronic and thermal Enthalpies= -630.268251
Sum of electronic and thermal Free Energies= -630.332464

Py_3^+(a)(I)Ar(I)

1	6	-0.29588	-0.93234	-0.7701
2	6	1.00717	-1.37069	-0.47916
3	6	1.16532	-1.29675	0.89909
4	6	-0.04511	-0.81758	1.42814
5	7	-0.91317	-0.63019	0.40116
6	1	-1.84813	-0.23619	0.48079
7	1	-0.81692	-0.85053	-1.70724
8	1	1.7245	-1.71441	-1.20336
9	1	2.03122	-1.57067	1.47559
10	1	-0.33482	-0.63858	2.44891
11	6	1.94991	1.79519	1.02637
12	6	0.63603	2.18502	0.75639
13	6	0.45124	2.09409	-0.62381
14	6	1.65538	1.64969	-1.17562
15	7	2.55164	1.49792	-0.15978
16	1	3.49444	1.16347	-0.2635
17	1	2.48742	1.74941	1.95718
18	1	-0.07794	2.5132	1.49108
19	1	-0.43741	2.33294	-1.1808
20	1	1.93177	1.47622	-2.20074
21	6	-4.01346	-0.72736	-1.19486
22	6	-3.65253	0.58575	-1.00339
23	6	-3.77653	0.86102	0.38938
24	6	-4.21336	-0.29581	0.99882
25	7	-4.34695	-1.25066	0.02676
26	1	-4.68107	-2.18592	0.18068
27	1	-4.08191	-1.31676	-2.09199
28	1	-3.36818	1.27883	-1.77648
29	1	-3.61451	1.80582	0.87973
30	1	-4.46293	-0.50145	2.02467
31	18	4.76693	-1.27813	-0.21661

Sum of electronic and zero-point Energies= -1157.847307
Sum of electronic and thermal Energies= -1157.829481
Sum of electronic and thermal Enthalpies= -1157.828537
Sum of electronic and thermal Free Energies= -1157.903044

Py_3^+(a)(I)Ar(II)

1	6	0.62071	-1.46361	-1.03529
2	6	1.90483	-2.02781	-0.94206
3	6	2.16553	-2.22985	0.40725
4	6	1.0347	-1.78916	1.11641
5	7	0.11274	-1.35561	0.21934
6	1	-0.78474	-0.93365	0.44909
7	1	0.04163	-1.1726	-1.89342
8	1	2.53846	-2.27122	-1.77686
9	1	3.04502	-2.66457	0.849
10	1	0.8323	-1.79352	2.1733
11	6	3.21135	0.73408	1.03743
12	6	1.90626	1.22378	0.95957
13	6	1.61251	1.41147	-0.39224
14	6	2.74271	1.03284	-1.11956
15	7	3.70242	0.64453	-0.23148
16	1	4.62438	0.32588	-0.47577
17	1	3.81478	0.48791	1.89347
18	1	1.27057	1.43334	1.80168
19	1	0.69996	1.79265	-0.81523
20	1	2.93078	1.0533	-2.17863
21	6	-3.08658	-1.01466	-1.11537
22	6	-2.63805	0.22401	-0.72071
23	6	-2.64656	0.24402	0.70424
24	6	-3.10477	-0.98497	1.12897
25	7	-3.36173	-1.73835	0.01509
26	1	-3.73773	-2.67035	0.02473
27	1	-3.25275	-1.42568	-2.09536
28	1	-2.37036	1.03437	-1.37688
29	1	-2.39645	1.07491	1.34173
30	1	-3.29263	-1.36421	2.11782
31	18	-1.63739	2.89326	-0.01284

Sum of electronic and zero-point Energies= -1157.847159
Sum of electronic and thermal Energies= -1157.829409
Sum of electronic and thermal Enthalpies= -1157.828464
Sum of electronic and thermal Free Energies= -1157.901712

Py_3^+(a)(I)Ar(III)

1	6	-0.29673	0.55038	-0.99281
2	6	-1.1542	1.65214	-0.82844
3	6	-1.28984	1.86395	0.53754
4	6	-0.5105	0.88987	1.18529
5	7	0.08977	0.12683	0.23723
6	1	0.7021	-0.66704	0.41411
7	1	0.07849	0.07943	-1.88373
8	1	-1.59109	2.22391	-1.62826
9	1	-1.85445	2.63591	1.03031
10	1	-0.32926	0.72913	2.23371
11	6	-3.6524	-0.23316	1.0202
12	6	-2.75323	-1.29009	0.86811
13	6	-2.5892	-1.50256	-0.50194
14	6	-3.3904	-0.57148	-1.16576
15	7	-4.03748	0.17266	-0.22348
16	1	-4.68663	0.91604	-0.41624
17	1	-4.0585	0.21554	1.90972
18	1	-2.30115	-1.84127	1.6737
19	1	-1.98152	-2.25114	-0.9788
20	1	-3.56483	-0.42401	-2.21707
21	6	2.91198	-1.29864	-1.10044
22	6	2.03095	-2.31374	-0.80753
23	6	1.99615	-2.44883	0.61078
24	6	2.86266	-1.51183	1.13307
25	7	3.40616	-0.82101	0.08424
26	1	4.08479	-0.08493	0.16959
27	1	3.2395	-0.89835	-2.04347
28	1	1.50061	-2.91421	-1.5266
29	1	1.44384	-3.1783	1.1783
30	1	3.15085	-1.30688	2.14887
31	18	2.49815	2.65004	-0.02769

Sum of electronic and zero-point Energies= -1157.847117
Sum of electronic and thermal Energies= -1157.829304
Sum of electronic and thermal Enthalpies= -1157.828360
Sum of electronic and thermal Free Energies= -1157.90235

Py_3^+(a)(I)Ar(IV)

1	6	0.54139	-1.08877	-0.45708
2	6	1.43689	-2.1154	-0.10973

3	6	1.82529	-1.89149	1.20491
4	6	1.15778	-0.73371	1.6407
5	7	0.37436	-0.28733	0.62596
6	1	-0.18345	0.56411	0.63938
7	1	0.00369	-0.90402	-1.3698
8	1	1.73198	-2.9262	-0.75252
9	1	2.48715	-2.49014	1.80592
10	1	1.17276	-0.24177	2.59766
11	6	4.15944	0.21575	0.55377
12	6	3.21287	1.1946	0.24633
13	6	2.79024	0.96721	-1.06469
14	6	3.48515	-0.14777	-1.5378
15	7	4.32061	-0.57284	-0.54708
16	1	4.94666	-1.35703	-0.6125
17	1	4.73815	0.06026	1.44732
18	1	2.89898	1.98391	0.90634
19	1	2.07707	1.54107	-1.6295
20	1	3.46625	-0.62498	-2.50181
21	6	-2.479	1.026	-0.83715
22	6	-1.47352	1.95823	-0.9464
23	6	-1.24658	2.49159	0.35554
24	6	-2.12706	1.86688	1.21348
25	7	-2.86246	0.97812	0.47689
26	1	-3.60261	0.40034	0.83539
27	1	-2.96356	0.41814	-1.58023
28	1	-0.98325	2.25085	-1.85904
29	1	-0.55805	3.27477	0.62366
30	1	-2.30339	2.00431	2.2656
31	18	-5.19185	-1.51322	-0.34876

Sum of electronic and zero-point Energies= -1157.846423
Sum of electronic and thermal Energies= -1157.828600
Sum of electronic and thermal Enthalpies= -1157.827656
Sum of electronic and thermal Free Energies= -1157.902887

Py_3^+(a)(I)Ar(V)

1	6	-1.03518	1.34018	1.01509
2	6	-0.21015	2.47275	0.90468
3	6	-0.07502	2.74967	-0.4499
4	6	-0.82272	1.78381	-1.14499
5	7	-1.40884	0.96423	-0.23509
6	1	-1.96397	0.13941	-0.45256
7	1	-1.39565	0.81526	1.88164
8	1	0.20719	3.02027	1.73152
9	1	0.46937	3.5589	-0.90424
10	1	-0.98976	1.66223	-2.20106
11	6	2.32049	0.68844	-1.0184
12	6	1.43622	-0.38741	-0.91995
13	6	1.26661	-0.66305	0.43766
14	6	2.04982	0.24926	1.14787
15	7	2.68973	1.045	0.24442
16	1	3.33311	1.78274	0.47424
17	1	2.72764	1.18046	-1.88413
18	1	0.99775	-0.90888	-1.75235
19	1	0.66578	-1.44004	0.876
20	1	2.21814	0.35007	2.20558
21	6	-3.97334	-0.97587	1.12056
22	6	-2.94216	-1.8042	0.74412
23	6	-2.93105	-1.84733	-0.68028
24	6	-3.96082	-1.04472	-1.12299
25	7	-4.57947	-0.51939	-0.02032
26	1	-5.38637	0.07933	-0.04355
27	1	-4.3349	-0.69708	2.09446
28	1	-2.29458	-2.34554	1.4123
29	1	-2.28039	-2.43491	-1.30536
30	1	-4.31339	-0.83422	-2.11714
31	18	4.92744	-1.65145	-0.00774

Sum of electronic and zero-point Energies= -1157.846325
Sum of electronic and thermal Energies= -1157.828437
Sum of electronic and thermal Enthalpies= -1157.827493
Sum of electronic and thermal Free Energies= -1157.902755

Py_3^+(a)(I)Ar(VI)

1	6	-1.18717	0.89838	-1.14941
2	6	-2.44064	1.51354	-1.31509
3	6	-2.83642	1.97779	-0.06725
4	6	-1.81626	1.64337	0.8397
5	7	-0.82733	1.01427	0.15448
6	1	0.01934	0.62241	0.56183
7	1	-0.53411	0.42923	-1.86339
8	1	-2.96436	1.61409	-2.24956
9	1	-3.73353	2.51842	0.17882
10	1	-1.73005	1.84513	1.89323
11	6	-4.08116	-0.80748	0.96064
12	6	-2.80626	-1.35565	1.10534
13	6	-2.38967	-1.77884	-0.15821
14	6	-3.41878	-1.48353	-1.05566
15	7	-4.43794	-0.91405	-0.35188
16	1	-5.31075	-0.60343	-0.74286
17	1	-4.75207	-0.39649	1.69444
18	1	-2.27055	-1.44541	2.03384
19	1	-1.46323	-2.26263	-0.4123
20	1	-3.50104	-1.6754	-2.11103
21	6	2.44706	0.16548	-0.75516
22	6	1.87365	-0.88722	-0.08166
23	6	1.77638	-0.51014	1.29005
24	6	2.29993	0.76014	1.40377
25	7	2.69585	1.15794	0.15527
26	1	3.15863	2.02523	-0.05305
27	1	2.72488	0.2744	-1.7885
28	1	1.59134	-1.83189	-0.51352
29	1	1.41658	-1.11488	2.10506
30	1	2.44456	1.38817	2.26492
31	18	6.02035	-0.61162	-0.37808

Sum of electronic and zero-point Energies= -1157.846130
Sum of electronic and thermal Energies= -1157.828300
Sum of electronic and thermal Enthalpies= -1157.827356
Sum of electronic and thermal Free Energies= -1157.901778

Py_3^+(a)(II)Ar(I)

1	6	-0.041	-1.39354	-0.94688
2	6	1.32131	-1.48365	-0.60835
3	6	1.39899	-1.47421	0.77785
4	6	0.08404	-1.37923	1.26441
5	7	-0.762	-1.35295	0.20193
6	1	-1.7744	-1.25212	0.25162
7	1	-0.52351	-1.38822	-1.90843
8	1	2.13214	-1.56855	-1.30999
9	1	2.28319	-1.54927	1.38581
10	1	-0.28423	-1.36383	2.27539
11	6	1.36104	1.68307	1.16448
12	6	0.01355	1.69678	0.80128
13	6	-0.04165	1.66205	-0.59346
14	6	1.2742	1.62841	-1.06013
15	7	2.1035	1.66186	0.02087
16	1	3.10781	1.62324	-0.0179
17	1	1.82386	1.72025	2.13497
18	1	-0.81464	1.73531	1.48611
19	1	-0.92219	1.66216	-1.21045
20	1	1.65915	1.61705	-2.06466
21	6	-3.86828	0.12232	1.05045
22	6	-3.94965	-1.09491	0.41057
23	6	-3.81461	-0.84921	-0.98752
24	6	-3.65545	0.50627	-1.15139
25	7	-3.68311	1.08349	0.09251
26	1	-3.66594	2.07285	0.26737
27	1	-3.96128	0.37858	2.09104
28	1	-4.13694	-2.04348	0.88491
29	1	-3.86562	-1.5782	-1.77809
30	1	-3.56108	1.10177	-2.04228
31	18	4.91111	-0.48107	-0.1878

Sum of electronic and zero-point Energies= -1157.846534

Sum of electronic and thermal Energies= -1157.828751
Sum of electronic and thermal Enthalpies= -1157.827807
Sum of electronic and thermal Free Energies= -1157.901990

Py$_3^+$(a)(II)Ar(II)

1	6	-1.98186	-1.43971	0.88665
2	6	-3.24602	-0.91783	0.55612
3	6	-3.27557	-0.76275	-0.82303
4	6	-2.03028	-1.19269	-1.31361
5	7	-1.28095	-1.61375	-0.26205
6	1	-0.31957	-1.94694	-0.31401
7	1	-1.56829	-1.71829	1.83998
8	1	-4.03573	-0.71354	1.25764
9	1	-4.09286	-0.41053	-1.42758
10	1	-1.66341	-1.2518	-2.32351
11	6	-1.89771	2.12108	-0.93207
12	6	-0.69001	1.511	-0.59277
13	6	-0.68519	1.339	0.79343
14	6	-1.8908	1.84775	1.27915
15	7	-2.60027	2.32944	0.2188
16	1	-3.50672	2.76145	0.27504
17	1	-2.27514	2.4411	-1.8874
18	1	0.0857	1.2393	-1.28616
19	1	0.09322	0.90114	1.39208
20	1	-2.26117	1.92524	2.28627
21	6	2.17405	-1.51408	-1.02874
22	6	1.72187	-2.70296	-0.4997
23	6	1.67165	-2.54528	0.91661
24	6	2.09359	-1.26838	1.20151
25	7	2.3889	-0.6511	0.0128
26	1	2.7934	0.26464	-0.07417
27	1	2.38953	-1.23187	-2.04416
28	1	1.50377	-3.5974	-1.05851
29	1	1.39422	-3.29359	1.63918
30	1	2.23909	-0.76816	2.1427
31	18	3.77155	2.11645	-0.15921

Sum of electronic and zero-point Energies= -1157.846436
Sum of electronic and thermal Energies= -1157.828706
Sum of electronic and thermal Enthalpies= -1157.827762
Sum of electronic and thermal Free Energies= -1157.901192

Py$_3^+$(a)(II)Ar(III)

1	6	0.17197	-1.35572	-0.67852
2	6	1.24207	-2.22184	-0.38946
3	6	1.50272	-2.10907	0.96942
4	6	0.58689	-1.17716	1.48931
5	7	-0.21256	-0.75619	0.4764
6	1	-0.95596	-0.06372	0.55235
7	1	-0.34483	-1.16704	-1.60279
8	1	1.73135	-2.86288	-1.10161
9	1	2.23925	-2.6425	1.54427
10	1	0.44549	-0.82952	2.49786
11	6	3.43977	0.43119	0.77676
12	6	2.31724	1.21798	0.51704
13	6	2.02485	1.09074	-0.84288
14	6	2.974	0.22785	-1.39251
15	7	3.82844	-0.14522	-0.3966
16	1	4.61738	-0.75876	-0.50842
17	1	3.98902	0.27704	1.68894
18	1	1.79512	1.81387	1.24424
19	1	1.22466	1.56375	-1.38338
20	1	3.11082	-0.10619	-2.40598
21	6	-1.81592	2.30296	1.18278
22	6	-2.62614	1.32381	0.65057
23	6	-2.46022	1.35909	-0.76488
24	6	-1.55697	2.35591	-1.04656
25	7	-1.16901	2.91687	0.14355
26	1	-0.56754	3.71682	0.23287
27	1	-1.68026	2.62918	2.19883
28	1	-3.29597	0.69022	1.20673
29	1	-2.96995	0.74661	-1.4883
30	1	-1.19253	2.73079	-1.98675
31	18	-3.33754	-2.25113	-0.28215

Sum of electronic and zero-point Energies= -1157.846115
Sum of electronic and thermal Energies= -1157.828316
Sum of electronic and thermal Enthalpies= -1157.827372
Sum of electronic and thermal Free Energies= -1157.901078

Py$_3^+$(a)(II)Ar(IV)

1	6	-1.17539	1.87492	1.06947
2	6	-0.2406	2.88747	0.78664
3	6	-0.16987	3.00327	-0.595
4	6	-1.06231	2.06087	-1.13454
5	7	-1.6682	1.41162	-0.10686
6	1	-2.33687	0.64875	-0.19932
7	1	-1.52931	1.49388	2.01128
8	1	0.28637	3.4688	1.52276
9	1	0.42491	3.69351	-1.16712
10	1	-1.31528	1.84935	-2.15885
11	6	1.97471	0.68251	-1.08625
12	6	0.97895	-0.24529	-0.78024
13	6	0.90496	-0.33288	0.61153
14	6	1.85802	0.54301	1.13382
15	7	2.50136	1.13488	0.08774
16	1	3.25009	1.80124	0.16895
17	1	2.35444	1.01223	-2.03728
18	1	0.3941	-0.78972	-1.4998
19	1	0.24665	-0.95548	1.1903
20	1	2.13362	0.74836	2.15323
21	6	-2.89507	-1.74289	-1.16085
22	6	-3.8002	-0.95209	-0.48781
23	6	-3.55353	-1.11759	0.90684
24	6	-2.5086	-2.00146	1.03563
25	7	-2.11396	-2.36819	-0.22566
26	1	-1.41827	-3.06372	-0.43056
27	1	-2.77138	-1.93328	-2.21233
28	1	-4.58121	-0.365	-0.94083
29	1	-4.10323	-0.66893	1.71653
30	1	-2.04382	-2.41989	1.91091
31	18	4.33936	-1.87374	-0.00611

Sum of electronic and zero-point Energies= -1157.84554
Sum of electronic and thermal Energies= -1157.827720
Sum of electronic and thermal Enthalpies= -1157.82677
Sum of electronic and thermal Free Energies= -1157.901077

Py$_3^+$(a)(II)Ar(V)

1	6	-1.66758	1.45845	-1.12158
2	6	-3.04041	1.57452	-0.83228
3	6	-3.16094	1.66901	0.54737
4	6	-1.86165	1.61649	1.07915
5	7	-0.98289	1.51232	0.04909
6	1	0.02766	1.41833	0.14114
7	1	-1.15477	1.38172	-2.06423
8	1	-3.82822	1.6088	-1.56421
9	1	-4.0623	1.79163	1.12189
10	1	-1.52615	1.68184	2.09963
11	6	-3.19908	-1.48438	1.08822
12	6	-1.82641	-1.51017	0.84327
13	6	-1.65144	-1.56544	-0.5417
14	6	-2.92165	-1.56907	-1.12068
15	7	-3.84145	-1.53391	-0.1151
16	1	-4.83932	-1.53213	-0.24008
17	1	-3.74401	-1.46346	2.01567
18	1	-1.05928	-1.4983	1.59717
19	1	-0.72234	-1.60875	-1.08051
20	1	-3.21774	-1.62401	-2.15355
21	6	2.15073	0.27408	1.23738
22	6	2.19433	1.37275	0.40599
23	6	2.10174	0.89319	-0.93444
24	6	2.00266	-0.47534	-0.87141
25	7	2.02003	-0.83934	0.45009
26	1	2.05319	-1.78639	0.78464

27	1	2.23111	0.19824	2.30731
28	1	2.33794	2.39258	0.72035
29	1	2.1552	1.47945	-1.83613
30	1	1.96663	-1.21152	-1.65453
31	18	5.4606	-0.48479	-0.08661

Sum of electronic and zero-point Energies= -1157.845428
Sum of electronic and thermal Energies= -1157.827637
Sum of electronic and thermal Enthalpies= -1157.826692
Sum of electronic and thermal Free Energies= -1157.900822

Py_3^+(p)(I)Ar(I)

1	6	1.64198	1.04136	-1.10361
2	6	3.01368	0.76005	-1.22845
3	6	3.13341	-0.59637	-1.49734
4	6	1.83548	-1.12827	-1.53402
5	7	0.95639	-0.1118	-1.31998
6	1	-0.05068	-0.21308	-1.26876
7	1	1.13363	1.97429	-0.93797
8	1	3.80665	1.48089	-1.13199
9	1	4.03947	-1.15526	-1.65308
10	1	1.4982	-2.12774	-1.74466
11	6	0.82064	0.48041	1.80277
12	6	2.16375	0.09896	1.9011
13	6	2.22099	-1.27387	1.67083
14	6	0.91202	-1.70879	1.43886
15	7	0.0814	-0.63306	1.53824
16	1	-0.92	-0.64268	1.37156
17	1	0.35664	1.44207	1.93413
18	1	2.98279	0.75853	2.12663
19	1	3.09388	-1.90219	1.68024
20	1	0.52904	-2.69612	1.24838
21	6	-3.05881	-1.34913	0.64463
22	6	-2.88659	-0.03214	0.28213
23	6	-2.41086	-0.02018	-1.06015
24	6	-2.30822	-1.33098	-1.47067
25	7	-2.69054	-2.12686	-0.42317
26	1	-2.78107	-3.12729	-0.4636
27	1	-3.44024	-1.78664	1.55068
28	1	-3.12115	0.82368	0.89158
29	1	-2.23355	0.84848	-1.67181
30	1	-2.04046	-1.75251	-2.42401
31	18	-1.20826	3.25174	0.15837

Sum of electronic and zero-point Energies= -1157.848280
Sum of electronic and thermal Energies= -1157.830710
Sum of electronic and thermal Enthalpies= -1157.829766
Sum of electronic and thermal Free Energies= -1157.900985

Py_3^+(p)(I)Ar(II)

1	6	-2.13706	-0.78045	-1.567
2	6	-3.26613	0.04325	-1.41728
3	6	-2.80969	1.30896	-1.07722
4	6	-1.40844	1.24482	-1.02239
5	7	-1.03143	-0.02053	-1.35067
6	1	-0.07542	-0.35716	-1.36809
7	1	-2.06217	-1.81143	-1.86449
8	1	-4.2864	-0.27116	-1.55097
9	1	-3.39981	2.18854	-0.88833
10	1	-0.68206	2.01317	-0.82626
11	6	-1.37423	-1.85773	1.23574
12	6	-2.45284	-1.05099	1.61377
13	6	-1.9349	0.19596	1.95885
14	6	-0.54839	0.12894	1.78701
15	7	-0.23147	-1.12674	1.36412
16	1	0.69963	-1.4461	1.11729
17	1	-1.34851	-2.88903	0.9294
18	1	-3.4829	-1.35918	1.63921
19	1	-2.47907	1.05539	2.30861
20	1	0.21361	0.86686	1.96272
21	6	2.94747	-1.49297	0.53119
22	6	2.31916	-2.30677	-0.38453
23	6	1.99795	-1.50362	-1.51634
24	6	2.4388	-0.22549	-1.25087
25	7	3.00061	-0.22997	-0.00236
26	1	3.46247	0.55581	0.42191
27	1	3.39193	-1.71504	1.48561
28	1	2.15371	-3.36511	-0.27556
29	1	1.56107	-1.84149	-2.44168
30	1	2.44116	0.66212	-1.85909

Sum of electronic and zero-point Energies= -1157.848225
Sum of electronic and thermal Energies= -1157.830638
Sum of electronic and thermal Enthalpies= -1157.829694
Sum of electronic and thermal Free Energies= -1157.901184

Py_3^+(p)(I)Ar(III)

1	6	2.88612	1.04575	0.23215
2	6	3.90112	0.26793	-0.35118
3	6	3.38703	-0.26555	-1.52459
4	6	2.06481	0.19202	-1.64479
5	7	1.8019	1.00386	-0.58587
6	1	0.91168	1.45299	-0.40069
7	1	2.89714	1.65047	1.12171
8	1	4.88803	0.12869	0.05424
9	1	3.8885	-0.90914	-2.22598
10	1	1.34234	0.03539	-2.42605
11	6	1.04117	-0.80434	1.91687
12	6	1.95209	-1.74605	1.42658
13	6	1.41048	-2.27173	0.25486
14	6	0.17734	-1.6438	0.05058
15	7	-0.03069	-0.77009	1.07607
16	1	-0.82259	-0.14107	1.16979
17	1	1.07408	-0.2002	2.80692
18	1	2.88357	-2.01281	1.89326
19	1	1.83504	-3.03124	-0.37728
20	1	-0.55997	-1.78791	-0.71928
21	6	-2.62628	1.25445	0.65665
22	6	-1.74372	2.04125	1.3629
23	6	-0.92164	2.71307	0.41499
24	6	-1.33012	2.31293	-0.83966
25	7	-2.35449	1.4174	-0.67694
26	1	-2.8902	1.01059	-1.42404
27	1	-3.43782	0.62974	0.98629
28	1	-1.72347	2.15412	2.43349
29	1	-0.16926	3.45403	0.62873
30	1	-1.00853	2.62179	-1.81923
31	18	-3.67867	-1.95496	-0.67371

Sum of electronic and zero-point Energies= -1157.847805
Sum of electronic and thermal Energies= -1157.830207
Sum of electronic and thermal Enthalpies= -1157.829263
Sum of electronic and thermal Free Energies= -1157.901703

Py_3^+(p)(I)Ar(IV)

1	6	-1.30525	-0.05721	-1.1906
2	6	-2.31569	0.91987	-1.28205
3	6	-1.68729	2.1438	-1.46343
4	6	-0.3069	1.90336	-1.47776
5	7	-0.10768	0.56233	-1.33837
6	1	0.7962	0.10479	-1.29477
7	1	-1.38517	-1.1267	-1.10296
8	1	-3.37204	0.71964	-1.21953
9	1	-2.15006	3.10818	-1.57857
10	1	0.51607	2.58055	-1.62328
11	6	-0.49083	-0.28662	1.6986
12	6	-1.39116	0.775	1.85271
13	6	-0.65049	1.95361	1.78924
14	6	0.68638	1.59421	1.59787
15	7	0.75988	0.23238	1.56155
16	1	1.60074	-0.30931	1.3906
17	1	-0.65954	-1.34918	1.7194
18	1	-2.45086	0.67347	2.00512
19	1	-1.01904	2.95994	1.88059
20	1	1.56965	2.20379	1.51953
21	6	3.72938	-0.99559	0.72653

22	6	2.85904	-1.90423	0.1674
23	6	2.57417	-1.46149	-1.15538
24	6	3.28137	-0.29687	-1.35943
25	7	3.96623	-0.0179	-0.20642
26	1	4.62187	0.73605	-0.09482
27	1	4.2211	-0.98747	1.68369
28	1	2.50602	-2.8065	0.63717
29	1	1.98027	-1.97544	-1.89268
30	1	3.38377	0.32138	-2.23445
31	18	-4.02135	-2.001	0.03697

Sum of electronic and zero-point Energies= -1157.847676
Sum of electronic and thermal Energies= -1157.830047
Sum of electronic and thermal Enthalpies= -1157.829103
Sum of electronic and thermal Free Energies= -1157.901539

Py$_3^+$(p)(I)Ar(V)

1	6	-2.55049	0.36174	-1.27166
2	6	-3.69869	-0.30354	-0.80817
3	6	-3.79982	-0.05033	0.55359
4	6	-2.71057	0.75819	0.90724
5	7	-1.98965	1.01296	-0.21934
6	1	-1.13134	1.54998	-0.2563
7	1	-2.15352	0.45298	-2.26716
8	1	-4.36488	-0.88991	-1.41649
9	1	-4.5611	-0.39962	1.22887
10	1	-2.44469	1.1936	1.85428
11	6	-0.15434	-1.53106	-0.86544
12	6	-1.14021	-2.35211	-0.30332
13	6	-1.1511	-2.10172	1.06724
14	6	-0.17636	-1.13341	1.31923
15	7	0.41766	-0.8095	0.1354
16	1	1.14455	-0.11293	0.00523
17	1	0.19017	-1.45235	-1.88212
18	1	-1.7608	-3.04813	-0.83869
19	1	-1.77846	-2.56169	1.81012
20	1	0.14362	-0.69045	2.24608
21	6	2.59118	1.66179	0.20096
22	6	2.02616	1.78939	-1.04796
23	6	0.89241	2.64073	-0.91625
24	6	0.80285	3.00228	0.41007
25	7	1.83014	2.39112	1.07915
26	1	2.05498	2.53992	2.0477
27	1	3.47745	1.14386	0.52351
28	1	2.41017	1.35906	-1.95714
29	1	0.26123	3.00103	-1.71167
30	1	0.1276	3.67183	0.9141
31	18	3.38844	-2.24283	-0.15222

Sum of electronic and zero-point Energies= -1157.847671
Sum of electronic and thermal Energies= -1157.830033
Sum of electronic and thermal Enthalpies= -1157.829080
Sum of electronic and thermal Free Energies= -1157.901599

Py$_3^+$(p)(I)Ar(VI)

1	6	0.50841	1.7077	1.18297
2	6	1.83561	1.93659	0.78432
3	6	1.86703	1.86756	-0.60156
4	6	0.55738	1.60326	-1.03425
5	7	-0.24269	1.53478	0.06225
6	1	-1.23429	1.33052	0.05663
7	1	0.06376	1.71402	2.16242
8	1	2.66074	2.12378	1.44873
9	1	2.72106	1.99084	-1.24363
10	1	0.15912	1.50933	-2.02901
11	6	0.0608	-1.34641	1.3444
12	6	1.39073	-1.35873	0.90814
13	6	1.36797	-1.46755	-0.48049
14	6	0.02451	-1.52387	-0.86852
15	7	-0.74638	-1.45861	0.25254
16	1	-1.7615	-1.44256	0.27053
17	1	-0.34591	-1.29746	2.33947
18	1	2.25547	-1.30289	1.54448
19	1	2.20468	-1.5329	-1.15232
20	1	-0.41365	-1.63183	-1.84542
21	6	-4.02616	-1.16267	-0.20385
22	6	-3.80478	-0.47516	0.96922
23	6	-3.54032	0.87943	0.62252
24	6	-3.60913	0.97638	-0.74984
25	7	-3.89203	-0.27236	-1.23951
26	1	-4.07368	-0.4848	-2.20529
27	1	-4.30674	-2.18652	-0.37996
28	1	-3.87171	-0.88781	1.96136
29	1	-3.39162	1.69586	1.30849
30	1	-3.52394	1.82671	-1.40371
31	18	5.06854	-0.54835	-0.31101

Sum of electronic and zero-point Energies= -1157.847530
Sum of electronic and thermal Energies= -1157.829884
Sum of electronic and thermal Enthalpies= -1157.828939
Sum of electronic and thermal Free Energies= -1157.901457

Py$_3^+$(p)(I)Ar(VII)

1	6	-2.35434	-1.41339	1.14529
2	6	-3.64587	-1.67022	0.65253
3	6	-3.55215	-1.74143	-0.73035
4	6	-2.20685	-1.52731	-1.06686
5	7	-1.50459	-1.35914	0.08674
6	1	-0.51174	-1.16307	0.14391
7	1	-1.99905	-1.332	2.1573
8	1	-4.52856	-1.78952	1.25614
9	1	-4.34706	-1.92611	-1.43147
10	1	-1.71882	-1.53697	-2.02542
11	6	-1.93002	1.63503	1.10269
12	6	-3.20425	1.6086	0.52517
13	6	-3.03033	1.57846	-0.8568
14	6	-1.65267	1.59047	-1.1004
15	7	-1.00827	1.63898	0.09874
16	1	-0.00232	1.61334	0.23291
17	1	-1.63331	1.68983	2.13566
18	1	-4.13337	1.62296	1.06668
19	1	-3.79638	1.56508	-1.61167
20	1	-1.10934	1.60076	-2.02909
21	6	2.2817	1.30235	-0.05675
22	6	1.97192	0.65262	1.11684
23	6	1.72224	-0.71132	0.79353
24	6	1.88685	-0.8471	-0.56749
25	7	2.21003	0.38381	-1.07297
26	1	2.47111	0.56255	-2.02719
27	1	2.58072	2.31927	-0.24163
28	1	1.97439	1.09423	2.09882
29	1	1.52066	-1.51104	1.4868
30	1	1.8465	-1.71741	-1.19917
31	18	5.43272	-0.471	0.02531

Sum of electronic and zero-point Energies= -1157.846935
Sum of electronic and thermal Energies= -1157.829302
Sum of electronic and thermal Enthalpies= -1157.828358
Sum of electronic and thermal Free Energies= -1157.901167

Py$_2^+$(a)Tol(I)

1	6	1.50244	-0.70138	1.09597
2	6	2.07395	-1.92121	0.69783
3	6	2.07395	-1.92121	-0.69783
4	6	1.50244	-0.70138	-1.09597
5	7	1.18979	0.00408	0
6	1	0.75209	0.91718	0
7	1	1.33695	-0.29528	2.07838
8	1	2.45694	-2.67377	1.36443
9	1	2.45694	-2.67377	-1.36443
10	1	1.33695	-0.29528	-2.07838
11	6	-0.84433	-3.08814	-1.10456
12	6	-1.34523	-1.84997	-0.69658
13	6	-1.34523	-1.84997	0.69658
14	6	-0.84433	-3.08814	1.10456
15	7	-0.56207	-3.81648	0

16	1	-0.19393	-4.75238	0
17	1	-0.70864	-3.49629	-2.09079
18	1	-1.67692	-1.06349	-1.35021
19	1	-1.67692	-1.06349	1.35021
20	1	-0.70864	-3.49629	2.09079
21	6	-0.31483	2.82325	-1.19683
22	6	-1.47398	2.05238	-1.2103
23	6	-2.06573	1.65703	0
24	6	-1.47398	2.05238	1.2103
25	6	-0.31483	2.82325	1.19683
26	6	0.27469	3.21956	0
27	1	0.12905	3.13901	-2.13247
28	1	-1.92319	1.77773	-2.15553
29	1	-2.97744	1.07547	0
30	1	-1.92319	1.77773	2.15553
31	1	0.12905	3.13901	2.13247
32	6	1.49336	4.1023	0
33	1	1.19798	5.15367	0
34	1	2.10437	3.93222	0.90765
35	1	2.10437	3.93222	-0.90765

Sum of electronic and zero-point Energies= -691.657604
Sum of electronic and thermal Energies= -691.640044
Sum of electronic and thermal Enthalpies= -691.639100
Sum of electronic and thermal Free Energies= -691.709309

Py_2^+(a)Tol(II)

1	6	-0.75288	-1.63541	-0.16357
2	6	-1.42556	-1.48107	1.06102
3	6	-2.78122	-1.38622	0.77357
4	6	-2.91512	-1.48793	-0.622
5	7	-1.67918	-1.66094	-1.1561
6	1	-1.46537	-1.71409	-2.14996
7	1	0.29323	-1.75874	-0.38002
8	1	-0.95716	-1.46679	2.02954
9	1	-3.59516	-1.28274	1.46958
10	1	-3.79778	-1.4808	-1.23751
11	6	-2.81345	1.75575	0.13896
12	6	-2.1697	1.52831	-1.07882
13	6	-0.80518	1.40453	-0.81109
14	6	-0.63643	1.55766	0.56651
15	7	-1.86332	1.78741	1.11648
16	1	-2.04159	1.94036	2.09426
17	1	-3.85242	1.92959	0.35767
18	1	-2.65398	1.48453	-2.03836
19	1	-0.01303	1.24048	-1.52
20	1	0.25766	1.55717	1.16486
21	6	2.46901	0.37019	1.16874
22	6	2.39043	-0.99762	1.43216
23	6	2.46228	-1.91507	0.38414
24	6	2.61158	-1.46477	-0.92817
25	6	2.68966	-0.09726	-1.1915
26	6	2.61877	0.8203	-0.14295
27	1	2.4128	1.09334	1.9952
28	1	2.27315	-1.35218	2.46646
29	1	2.40097	-2.99323	0.59176
30	1	2.66814	-2.18833	-1.75434
31	1	2.80732	0.25794	-2.22565
32	6	2.70528	2.33007	-0.43399
33	1	3.31548	2.49239	-1.29783
34	1	1.72338	2.71557	-0.61337
35	1	3.13708	2.83126	0.40699

Sum of electronic and zero-point Energies= -691.652323
Sum of electronic and thermal Energies= -691.634772
Sum of electronic and thermal Enthalpies= -691.633827
Sum of electronic and thermal Free Energies= -691.704108

Py_2^+(a)Tol(III)

1	6	-0.3935	0.77839	-0.95775
2	6	-0.30976	0.57854	0.42462
3	6	-0.47016	-0.78326	0.6566
4	6	-0.65028	-1.39898	-0.58629
5	7	-0.59178	-0.43394	-1.54626
6	1	-0.68305	-0.59181	-2.53472
7	1	-0.30199	1.67919	-1.53796
8	1	-0.12314	1.34899	1.15042
9	1	-0.434	-1.29537	1.6009
10	1	-0.78582	-2.43603	-0.8368
11	6	-3.69182	-0.81851	0.77732
12	6	-3.76018	-0.61376	-0.59818
13	6	-3.59643	0.7584	-0.82439
14	6	-3.43121	1.36677	0.41724
15	7	-3.50585	0.39534	1.37264
16	1	-3.43509	0.55004	2.36337
17	1	-3.79716	-1.71886	1.35643
18	1	-3.93844	-1.3802	-1.33192
19	1	-3.62239	1.2725	-1.76917
20	1	-3.30629	2.40309	0.67713
21	6	3.05132	0.31739	1.29986
22	6	2.92172	-1.05575	1.42955
23	6	2.74666	-1.85309	0.29755
24	6	2.71294	-1.26051	-0.96133
25	6	2.84062	0.11605	-1.0861
26	6	3.01109	0.92892	0.04039
27	1	3.19513	0.92914	2.1814
28	1	2.96533	-1.51188	2.40926
29	1	2.66119	-2.92641	0.39727
30	1	2.60326	-1.87479	-1.84509
31	1	2.8317	0.56984	-2.06921
32	6	3.19788	2.41336	-0.09668
33	1	2.7876	2.78807	-1.03393
34	1	2.73244	2.95427	0.72767
35	1	4.26074	2.66654	-0.08421

Sum of electronic and zero-point Energies= -691.650388
Sum of electronic and thermal Energies= -691.632603
Sum of electronic and thermal Enthalpies= -691.631659
Sum of electronic and thermal Free Energies= -691.702318

Py_2^+(a)Tol(IV)

1	6	0.59997	0.41706	1.11153
2	6	-0.73603	0.45801	0.69484
3	6	-0.73603	0.45801	-0.69484
4	6	0.59997	0.41706	-1.11153
5	7	1.38635	0.3861	0
6	1	2.39005	0.33151	0
7	1	1.02009	0.38092	2.10082
8	1	-1.58819	0.452	1.34998
9	1	-1.58819	0.452	-1.34998
10	1	1.02009	0.38092	-2.10082
11	6	-0.46097	3.62149	-1.11459
12	6	0.86831	3.5716	-0.69998
13	6	0.86831	3.5716	0.69998
14	6	-0.46097	3.62149	1.11459
15	7	-1.24608	3.66575	0
16	1	-2.24998	3.71971	0
17	1	-0.88384	3.66549	-2.10274
18	1	1.72263	3.5673	-1.35392
19	1	1.72263	3.5673	1.35392
20	1	-0.88384	3.66549	2.10274
21	6	0.09729	-2.8881	1.19615
22	6	-1.29744	-2.9097	1.19887
23	6	-1.99971	-2.92302	0
24	6	-1.29744	-2.9097	-1.19887
25	6	0.09729	-2.8881	-1.19615
26	6	0.81488	-2.88601	0
27	1	0.63136	-2.89795	2.13822
28	1	-1.83034	-2.92865	2.14002
29	1	-3.08035	-2.95296	0
30	1	-1.83034	-2.92865	-2.14002
31	1	0.63136	-2.89795	-2.13822
32	6	2.31986	-2.91854	0
33	1	2.73311	-2.43543	-0.88629

34	1	2.73311	-2.43543	0.88629
35	1	2.68509	-3.94756	0

Sum of electronic and zero-point Energies= -691.650071
Sum of electronic and thermal Energies= -691.632352
Sum of electronic and thermal Enthalpies= -691.631408
Sum of electronic and thermal Free Energies= -691.701568

Py_2^+(p)Tol(I)

1	6	2.33073	-1.0582	-1.17646
2	6	3.62722	-0.78212	-0.70818
3	6	3.59317	-0.87461	0.67619
4	6	2.27833	-1.2054	1.03805
5	7	1.54662	-1.33526	-0.102
6	1	0.55649	-1.54877	-0.14121
7	1	1.95448	-1.13046	-2.18158
8	1	4.47489	-0.54826	-1.32825
9	1	4.40858	-0.72625	1.36224
10	1	1.85064	-1.40024	2.00563
11	6	0.73822	1.57836	-1.11843
12	6	1.93422	2.06042	-0.57505
13	6	1.82054	1.97765	0.811
14	6	0.55575	1.4495	1.09132
15	7	-0.08548	1.2293	-0.09035
16	1	-1.00311	0.80914	-0.19861
17	1	0.41834	1.502	-2.14307
18	1	2.76904	2.43371	-1.14113
19	1	2.54873	2.27355	1.54517
20	1	0.07537	1.25529	2.03438
21	6	-2.39551	-1.65201	-1.13122
22	6	-2.06997	-2.24159	0.09062
23	6	-2.12869	-1.49125	1.26482
24	6	-2.51194	-0.15032	1.21737
25	6	-2.83693	0.43918	-0.00427
26	6	-2.7791	-0.3118	-1.17862
27	1	-2.34958	-2.24389	-2.05681
28	1	-1.76834	-3.29842	0.12775
29	1	-1.87272	-1.95607	2.228
30	1	-2.55811	0.44111	2.14331
31	1	-3.03553	0.1531	-2.14156
32	6	-3.25993	1.91901	-0.05711
33	1	-2.95658	2.34354	-0.99127
34	1	-4.32334	1.98961	0.03818
35	1	-2.79378	2.45207	0.74505

Sum of electronic and zero-point Energies= -691.658088
Sum of electronic and thermal Energies= -691.640615
Sum of electronic and thermal Enthalpies= -691.639671
Sum of electronic and thermal Free Energies= -691.709535

Py_2^+(a)Bz(I)

1	6	1.55102	-0.6236	1.09546
2	6	1.92332	-1.92948	0.73582
3	6	1.92332	-1.92948	-0.73582
4	6	1.55102	-0.6236	-1.09546
5	7	1.37614	0.09505	0
6	1	1.09843	1.06743	0
7	1	1.43723	-0.17184	2.06518
8	1	2.16753	-2.71716	1.42669
9	1	2.16753	-2.71716	-1.42669
10	1	1.43723	-0.17184	-2.06518
11	6	-1.11156	-2.64759	-1.11219
12	6	-1.41723	-1.34321	-0.71834
13	6	-1.41723	-1.34321	0.71834
14	6	-1.11156	-2.64759	1.11219
15	7	-0.96203	-3.40261	0
16	1	-0.74673	-4.38492	0
17	1	-1.02902	-3.0798	-2.09404
18	1	-1.61313	-0.52022	-1.38179
19	1	-1.61313	-0.52022	1.38179
20	1	-1.02902	-3.0798	2.09404
21	6	0.22523	3.09086	1.24221
22	6	-1.03522	2.50275	1.17264
23	6	-1.61634	2.2445	0
24	6	-1.03522	2.50275	-1.17264
25	6	0.22523	3.09086	-1.24221
26	6	0.90503	3.42056	0
27	1	0.66908	3.31057	2.20368
28	1	-1.57202	2.26925	2.08241
29	1	-2.60486	1.80834	0
30	1	-1.57202	2.26925	-2.08241
31	1	0.66908	3.31057	-2.20368
32	1	1.87617	3.89333	0

Sum of electronic and zero-point Energies= -652.348842
Sum of electronic and thermal Energies= -652.333067
Sum of electronic and thermal Enthalpies= -652.332123
Sum of electronic and thermal Free Energies= -652.397782

Py_2^+(p)Bz(I)

1	6	1.25829	-1.85635	1.11093
2	6	1.22251	-3.19668	0.69408
3	6	1.22251	-3.19668	-0.69408
4	6	1.25829	-1.85635	-1.11093
5	7	1.3062	-1.07305	0
6	1	1.31794	-0.06318	0
7	1	1.30845	-1.43477	2.09923
8	1	1.20221	-4.05047	1.3485
9	1	1.20221	-4.05047	-1.3485
10	1	1.30845	-1.43477	-2.09923
11	6	-1.66029	-0.71388	1.11125
12	6	-1.89101	-2.03001	0.69692
13	6	-1.89101	-2.03001	-0.69692
14	6	-1.66029	-0.71388	-1.11125
15	7	-1.5333	0.06459	0
16	1	-1.35937	1.06087	0
17	1	-1.61451	-0.29	2.09929
18	1	-2.05191	-2.86889	1.3504
19	1	-2.05191	-2.86889	-1.3504
20	1	-1.61451	-0.29	-2.09929
21	6	0.39542	2.77569	1.39459
22	6	1.50647	2.30494	0.69813
23	6	1.50647	2.30494	-0.69813
24	6	0.39542	2.77569	-1.39459
25	6	-0.7075	3.25842	-0.69861
26	6	-0.7075	3.25842	0.69861
27	1	0.40303	2.79647	2.4758
28	1	2.3823	1.97272	1.24062
29	1	2.3823	1.97272	-1.24062
30	1	0.40303	2.79647	-2.4758
31	1	-1.55728	3.65287	-1.2391
32	1	-1.55728	3.65287	1.2391

Sum of electronic and zero-point Energies= -652.348911
Sum of electronic and thermal Energies= -652.333158
Sum of electronic and thermal Enthalpies= -652.332214
Sum of electronic and thermal Free Energies= -652.399355

at CAM-B3LYP-D3/aug-cc-pVTZ

H_2O

1	8	0	0	0.11081
2	1	0	0.78398	-0.44325
3	1	0	-0.78398	-0.44325

Sum of electronic and zero-point Energies= -76.416663
Sum of electronic and thermal Energies= -76.413828
Sum of electronic and thermal Enthalpies= -76.412884
Sum of electronic and thermal Free Energies= -76.434298

$(H_2O)_2$

1	8	-1.51026	0.00103	-0.12134
2	1	-1.92685	-0.00652	0.74435
3	1	-0.55632	-2.33E-4	0.05209
4	8	1.38889	-8.25E-4	0.11231
5	1	1.72719	0.76871	-0.35658
6	1	1.727	-0.76361	-0.36761

Sum of electronic and zero-point Energies= -152.839079
Sum of electronic and thermal Energies= -152.833280

Sum of electronic and thermal Enthalpies= -152.832336
Sum of electronic and thermal Free Energies= -152.865296

Py

1	6	0	1.10113	0.35062
2	6	0	0.68085	-1.00977
3	6	0	-0.68085	-1.00977
4	6	0	-1.10113	0.35062
5	7	0	0	1.13511
6	1	0	0	2.14483
7	1	0	2.09622	0.76713
8	1	0	1.34448	-1.85751
9	1	0	-1.34448	-1.85751
10	1	0	-2.09622	0.76713

Sum of electronic and zero-point Energies= -210.054113
Sum of electronic and thermal Energies= -210.050238
Sum of electronic and thermal Enthalpies= -210.049294
Sum of electronic and thermal Free Energies= -210.079795

Py^+

1	6	0	1.10622	0.35168
2	6	0	0.6841	-1.01351
3	6	0	-0.6841	-1.01351
4	6	0	-1.10622	0.35168
5	7	0	0	1.13966
6	1	0	0	2.14989
7	1	0	2.10118	0.76883
8	1	0	1.34782	-1.86156
9	1	0	-1.34782	-1.86156
10	1	0	-2.10118	0.76883

Sum of electronic and zero-point Energies= -209.756594
Sum of electronic and thermal Energies= -209.752659
Sum of electronic and thermal Enthalpies= -209.751715
Sum of electronic and thermal Free Energies= -209.782975

$Py_2^+(a)$

1	6	1.53112	0.6022	1.10692
2	6	1.53112	-0.72852	0.6934
3	6	1.53112	-0.72852	-0.6934
4	6	1.53112	0.6022	-1.10692
5	7	1.5532	1.38552	0
6	1	1.56719	2.39093	0
7	1	1.56015	1.02335	2.09658
8	1	1.56267	-1.58022	1.34942
9	1	1.56267	-1.58022	-1.34942
10	1	1.56015	1.02335	-2.09658
11	6	-1.53112	-0.6022	-1.10692
12	6	-1.53112	0.72852	-0.6934
13	6	-1.53112	0.72852	0.6934
14	6	-1.53112	-0.6022	1.10692
15	7	-1.5532	-1.38552	0
16	1	-1.56719	-2.39093	0
17	1	-1.56015	-1.02335	-2.09658
18	1	-1.56267	1.58022	-1.34942
19	1	-1.56267	1.58022	1.34942
20	1	-1.56015	-1.02335	2.09658

Sum of electronic and zero-point Energies= -419.844048
Sum of electronic and thermal Energies= -419.834871
Sum of electronic and thermal Enthalpies= -419.833926
Sum of electronic and thermal Free Energies= -419.879501

$Py_2^+(a)H_2O$

1	7	2.62766	-0.15662	-3.12E-4
2	1	3.41329	0.47123	-6.49E-4
3	6	2.00645	-0.63907	1.11361
4	6	2.00659	-0.64047	-1.11367
5	6	0.97393	-1.48229	0.69847
6	6	0.97401	-1.48316	-0.69764
7	1	2.35107	-0.39189	2.10238
8	1	2.35119	-0.3944	-2.10273
9	1	0.3284	-2.03995	1.35379
10	1	0.32854	-2.04158	-1.35235
11	7	-1.53869	0.49356	4.15E-4
12	1	-2.33301	-0.14935	7.13E-4
13	6	-0.9212	0.98469	1.10676
14	6	-0.9223	0.98462	-1.10664
15	6	0.11087	1.84466	0.69379
16	6	0.11021	1.84453	-0.6948
17	1	-1.26023	0.7335	2.09686
18	1	-1.26226	0.73316	-2.09635
19	1	0.74821	2.40949	1.35118
20	1	0.74697	2.40922	-1.35286
21	8	-3.8077	-1.19725	-3.3E-5
22	1	-4.70438	-0.84557	4.41E-4
23	1	-3.9004	-2.15543	-9.6E-5

Sum of electronic and zero-point Energies= -496.279568
Sum of electronic and thermal Energies= -496.266603
Sum of electronic and thermal Enthalpies= -496.265658
Sum of electronic and thermal Free Energies= -496.321650

$Py_2^+(a)(H_2O)_2$

1	7	-0.84251	0.54042	-4.2E-5
2	1	-1.64704	-0.10405	-8.9E-5
3	6	-0.21681	1.02016	1.10435
4	6	-0.21684	1.02041	-1.10434
5	6	0.83298	1.86366	0.69344
6	6	0.83296	1.86382	-0.69327
7	1	-0.55927	0.7725	2.09418
8	1	-0.55936	0.77303	-2.09423
9	1	1.48074	2.41524	1.35189
10	1	1.48067	2.4156	-1.35161
11	7	3.29832	-0.21026	-1.2E-4
12	1	4.09877	0.39816	-2.12E-4
13	6	2.66446	-0.67678	1.11398
14	6	2.66417	-0.67674	-1.11407
15	6	1.61113	-1.49132	0.69881
16	6	1.61095	-1.49131	-0.69866
17	1	3.01532	-0.43837	2.10262
18	1	3.01481	-0.43838	-2.10279
19	1	0.94863	-2.03023	1.35283
20	1	0.94836	-2.03027	-1.35253
21	8	-3.02714	-1.13113	-2.28E-4
22	1	-3.94422	-0.7891	-7.4E-5
23	1	-3.09183	-2.08973	-2.66E-4
24	8	-5.57069	-0.0823	1.52E-4
25	1	-6.14597	-0.02518	-0.7694
26	1	-6.14567	-0.02436	0.76987

Sum of electronic and zero-point Energies= -572.712369
Sum of electronic and thermal Energies= -572.696149
Sum of electronic and thermal Enthalpies= -572.695205
Sum of electronic and thermal Free Energies= -572.760794

$Py_3^+(a)(I)$

1	6	-0.15738	1.04385	-1.02274
2	6	-1.31231	1.83825	-0.91774
3	6	-1.52968	2.06669	0.43503
4	6	-0.50166	1.41112	1.13452
5	7	0.32179	0.8245	0.22869
6	1	1.12392	0.23799	0.44981
7	1	0.35617	0.6612	-1.8866
8	1	-1.88921	2.2083	-1.74713
9	1	-2.31142	2.65289	0.88547
10	1	-0.30156	1.36241	2.19073
11	6	-3.11436	-0.65605	1.02764
12	6	-1.9248	-1.38145	0.93897
13	6	-1.67227	-1.60199	-0.4162
14	6	-2.71136	-1.00722	-1.13471
15	7	-3.58041	-0.4578	-0.23838
16	1	-4.42604	0.03207	-0.47538
17	1	-3.66026	-0.31282	1.88886
18	1	-1.33951	-1.71876	1.77612
19	1	-0.84799	-2.14209	-0.84726
20	1	-2.90043	-0.97699	-2.19337
21	6	3.39911	-0.09358	-1.12057

22	6	2.72566	-1.23114	-0.74159
23	6	2.73093	-1.27249	0.68292
24	6	3.41247	-0.15781	1.12308
25	7	3.8061	0.54939	0.01887
26	1	4.3508	1.39375	0.04016
27	1	3.6392	0.29261	-2.09533
28	1	2.30991	-1.96732	-1.408
29	1	2.32922	-2.05038	1.30969
30	1	3.66882	0.16533	2.11646

Sum of electronic and zero-point Energies= -629.918945
Sum of electronic and thermal Energies= -629.903980
Sum of electronic and thermal Enthalpies= -629.903036
Sum of electronic and thermal Free Energies= -629.96704

Py_3^+(a)(III)

1	6	-3.21254	-0.51312	1.10435
2	6	-3.15468	0.81336	0.73684
3	6	-3.15483	0.85875	-0.66823
4	6	-3.21242	-0.44127	-1.12059
5	7	-3.25306	-1.26036	-0.03358
6	1	-3.34644	-2.25942	-0.06579
7	1	-3.26926	-0.96859	2.07689
8	1	-3.15052	1.64893	1.41457
9	1	-3.15023	1.73638	-1.29052
10	1	-3.26625	-0.83304	-2.12064
11	6	-4.89E-4	0.68671	-1.09768
12	6	-0.01909	-0.6632	-0.7335
13	6	-0.01128	-0.71515	0.64629
14	6	0.01365	0.60371	1.11074
15	7	0.01469	1.42668	0.03594
16	1	0.04326	2.43039	0.07331
17	1	-0.00319	1.14255	-2.07147
18	1	-0.04076	-1.48682	-1.42372
19	1	-0.02004	-1.5882	1.27305
20	1	0.03265	0.98446	2.11601
21	6	3.18568	-0.52788	1.10571
22	6	3.18048	0.79967	0.73682
23	6	3.19835	0.84379	-0.66837
24	6	3.21468	-0.45786	-1.11908
25	7	3.21392	-1.27676	-0.0313
26	1	3.2656	-2.27883	-0.06223
27	1	3.21525	-0.98405	2.0791
28	1	3.20153	1.63507	1.41457
29	1	3.23473	1.72017	-1.29145
30	1	3.25971	-0.85105	-2.11898

Sum of electronic and zero-point Energies= -629.911389
Sum of electronic and thermal Energies= -629.896228
Sum of electronic and thermal Enthalpies= -629.895284
Sum of electronic and thermal Free Energies= -629.958480

Py_2^+(a)Tol(I)

1	6	1.50221	-0.71444	1.10851
2	6	2.07221	-1.92965	0.69494
3	6	2.07221	-1.92965	-0.69494
4	6	1.50221	-0.71444	-1.10851
5	7	1.18806	0.00459	0
6	1	0.75061	0.91787	0
7	1	1.3391	-0.3199	2.09604
8	1	2.45587	-2.69037	1.35182
9	1	2.45587	-2.69037	-1.35182
10	1	1.3391	-0.3199	-2.09604
11	6	-0.8475	-3.07737	-1.11355
12	6	-1.3491	-1.84205	-0.69753
13	6	-1.3491	-1.84205	0.69753
14	6	-0.8475	-3.07737	1.11355
15	7	-0.56619	-3.81324	0
16	1	-0.19776	-4.74904	0
17	1	-0.71077	-3.47863	-2.10246
18	1	-1.68088	-1.05166	-1.34634
19	1	-1.68088	-1.05166	1.34634
20	1	-0.71077	-3.47863	2.10246
21	6	-0.31367	2.81938	-1.19962
22	6	-1.4755	2.05262	-1.20122
23	6	-2.06256	1.66844	0
24	6	-1.4755	2.05262	1.20122
25	6	-0.31367	2.81938	1.19962
26	6	0.28334	3.22287	0
27	1	0.12634	3.12655	-2.13993
28	1	-1.93072	1.77257	-2.14196
29	1	-2.97657	1.0905	0
30	1	-1.93072	1.77257	2.14196
31	1	0.12634	3.12655	2.13993
32	6	1.50508	4.10147	0
33	1	1.21337	5.15388	0
34	1	2.12015	3.93586	0.88388
35	1	2.12015	3.93586	-0.88388

Sum of electronic and zero-point Energies= -691.238613
Sum of electronic and thermal Energies= -691.221314
Sum of electronic and thermal Enthalpies= -691.220370
Sum of electronic and thermal Free Energies= -691.289345

Py_2^+(p)Tol(I)

1	6	-2.46174	-1.19399	0.87632
2	6	-3.69677	-0.88236	0.29626
3	6	-3.48047	-0.70543	-1.05607
4	6	-2.11379	-0.91046	-1.28853
5	7	-1.53513	-1.23711	-0.1102
6	1	-0.55015	-1.41698	0.01996
7	1	-2.21441	-1.44311	1.89298
8	1	-4.62946	-0.80193	0.8259
9	1	-4.20819	-0.45832	-1.80864
10	1	-1.55804	-0.91391	-2.20917
11	6	-0.90137	1.41807	1.40204
12	6	-2.09472	1.90785	0.88004
13	6	-1.91167	2.0617	-0.48879
14	6	-0.60915	1.66133	-0.77788
15	7	-0.01339	1.28906	0.3828
16	1	0.93549	0.95024	0.46968
17	1	-0.62368	1.19741	2.41799
18	1	-2.97678	2.13639	1.45076
19	1	-2.62107	2.43595	-1.20481
20	1	-0.0699	1.65824	-1.70881
21	6	2.29399	-1.16217	1.47958
22	6	1.72161	-2.01832	0.54965
23	6	1.82567	-1.72351	-0.80179
24	6	2.49857	-0.58477	-1.21865
25	6	3.09517	0.27216	-0.29907
26	6	2.97465	-0.03146	1.05692
27	1	2.23459	-1.38996	2.53469
28	1	1.23312	-2.92695	0.87558
29	1	1.40763	-2.39783	-1.53759
30	1	2.59061	-0.37494	-2.27616
31	1	3.43973	0.61726	1.78767
32	6	3.88406	1.46605	-0.7499
33	1	3.78842	2.29699	-0.05325
34	1	4.94354	1.21507	-0.80944
35	1	3.57391	1.80449	-1.73633

Sum of electronic and zero-point Energies= -691.238516
Sum of electronic and thermal Energies= -691.221240
Sum of electronic and thermal Enthalpies= -691.220296
Sum of electronic and thermal Free Energies= -691.290355
